# The global, regional, and national burdens of dementia in 204 countries and territories from 1990 to 2021: A trend analysis based on the Global Burden of Disease Study 2021

**DOI:** 10.1097/MD.0000000000041836

**Published:** 2025-03-14

**Authors:** Songxin Zhong, Chao Xiao, Rida Li, Yining Lan, Chi Gong, Changqiang Feng, Hengchang Qi, Yanni Lin, Chao Qin

**Affiliations:** a Department of Neurology, The First People’s Hospital of Yulin Affiliated to Guangxi Medical University, Yulin, People’s Republic of China; b Department of Neurology, The First Affiliated Hospital of Guangxi Medical University, Nanning, People’s Republic of China.

**Keywords:** Alzheimer’s disease, dementia, Global Burden of Disease, public health resources

## Abstract

The global population is aging, and as a consequence, the prevalence of dementia is increasing rapidly. This study aims to analyze trends in the Global Burden of Disease (GBD) and health inequalities for dementia over the period 1990 to 2021. The incidence, prevalence, and disability-adjusted life year rates of dementia in the GBD 2021 database were analyzed at the global, national, and regional levels for the period 1990 to 2021 using Joinpoint 4.9.1.0 software. The trends over the period were assessed using a combination of age-standardized rates, average annual percentage changes (AAPCs), and a sociodemographic index. The analysis revealed that, from 1990 to 2021, the global AAPC in dementia incidence, prevalence, and disability-adjusted life years amounted to 0.06 (95% confidence interval [CI]: 0.05–0.09), 0.09 (95% CI: 0.08–0.10), and 0.03 (95% CI: 0.01–0.05), respectively. Conversely, the mean AAPC in age-standardized mortality rate remained stable at 0 (95% CI: −0.02 to 0.03). The age-standardized incidence rate and age-standardized prevalence rate of dementia exhibited a positive association with sociodemographic index during the study period. The 3 regions with the highest mean AAPC in age-standardized mortality rate among the 21 GBD regions were South Africa, Central Sub-Saharan Africa, and Eastern Sub-Saharan Africa. The findings of the study indicate that the burden of dementia increases with age and is projected to remain on an upward trend until 2040. The GBD has increased significantly from 1990 to 2021, and the prevention and control of dementia represents a long-term and formidable challenge.

## 1. Introduction

Dementia is a syndrome characterized by chronic or progressive dysfunction of the cerebral cortex and subcortical functions, leading to complex cognitive decline, and is often accompanied by disorders of mood, personality, and behavior. It is common to distinguish between primary degenerative dementias, such as Alzheimer disease, dementia with Lewy bodies, frontotemporal dementia, and dementias secondary to another disease process, such as vascular dementia and HIV dementia.^[1]^ Alzheimer disease is the predominant cause of dementia, accounting for approximately 60% to 80% of cases. Neurological alterations include the accumulation of aberrant Aβ amyloid and phosphorylated tau proteins, as well as neuronal degeneration.^[2]^ Dementia primarily emerges from progressive degenerative processes that transpire over an extended period. Various diseases demonstrate a predilection for specific brain regions, giving rise to distinct clinical patterns of dementia. As the disease progresses, there is a propensity for more cortical areas to be impacted, resulting in a global deterioration in cognitive function. Previous epigenetic evidence indicates that dementia is not characterized by sudden and well-defined states but rather by gradual alterations in crucial cellular signaling pathways, which ultimately transform an otherwise healthy state into a dysfunctional one due to neurodegeneration.^[3]^

Alzheimer disease and other forms of dementia present substantial global public health challenges. Over the past decade, both domestic and international scholars have achieved remarkable progress in dementia research via population cohorts, fundamental research, and clinical trials. Currently, approximately 55 million individuals worldwide are afflicted with dementia, with approximately 35 million solely affected by Alzheimer disease. This condition ranks as the 7th leading cause of death globally and constitutes a major public health concern that profoundly influences the well-being of populations worldwide. The treatment process of dementia is complex, featuring limited efficacy and high costs. Family members encounter significant psychological stress and mental burden during the caregiving process. Globally, a new case of dementia emerges every 4 seconds, and it is projected that the number of people with dementia worldwide will amount to 153 million by 2050. In 2019, the global societal cost attributed to dementia was estimated at US $1313.4 billion, equivalent to US $23,796 per person affected by this condition. This encompasses direct medical costs reaching US $213.2 billion (16%), direct social sector costs (including long-term care) totaling US $448.7 billion (34%), and informal care costs amounting to US $651.4 billion (50%).^[4]^ According to the China Alzheimer’s Disease Report 2024, in 2019 alone, a total of 25,276,989 person-years were burdened by Alzheimer disease and other dementias (ADOD) globally, among which China accounted for approximately 23.6%. The average annual cost for individuals with dementia reached as high as US $3350. For patients weighing approximately 60 kg, monthly expenses could soar to US $2330, resulting in an annual expenditure of US $27,960 if treated with lenkanizumab.^[5]^

In their deliberations, the members of the 2020 Lancet committee examined each of the 12 risk factors associated with dementia. These factors included lower levels of education, traumatic brain injury, a sedentary lifestyle, tobacco use, excessive alcohol consumption, hypertension, obesity, diabetes mellitus, hearing impairment, depression, social isolation, and air pollution. A reduction in these aforementioned factors was found to have prevented 40% of dementia cases. The subsequent 2024 Lancet committee once again identified visual impairment and hypercholesterolemia as 2 potentially modifiable risk factors.^[6]^ Collectively, by proactively alleviating fourteen risk factors, it is theoretically feasible to prevent half of all dementias. Hence, long-term monitoring of dementia risk factors and epidemiological trends is indispensable for global dementia prevention.

The 2021 Global Burden of Disease (GBD) study adopts a distinctive framework that integrates a variety of factors to comprehensively evaluate the burden of disease. Based on the GBD 2021 data, the study offers detailed analyses of the trends in the burden of disease related to dementia, covering multiple dimensions, such as gender, age, nationality, and region. Furthermore, the study presents a comprehensive analysis of the disparities in the risk of developing dementia across countries and regions, thereby guiding targeted prevention and treatment.

## 2. Methods

### 2.1. Data origins and research entities

The data analyzed in this study were obtained from the GBD 2021 database, which offers the most up-to-date epidemiological estimates of the burden of 371 diseases and injuries across 21 GBD regions and 204 countries and territories from 1990 to 2021.^[7]^ The data can be accessed for free through the global health data exchange center (https://ghdx.healthdata.org/gbd-2021/sources). Dementia is largely age-dependent; accordingly, the study population encompassed solely patients with dementia aged 40 years and above, excluding those diagnosed with dementia prior to the age of 40. We analyzed the trends in incidence, prevalence, mortality, and disability-adjusted life year rates of dementia patients globally, by continent, and by region in the 2021 GBD Study. This study conformed to the guidelines for Accurate and Transparent Health Estimates Reporting.^[8]^

### 2.2. Indicators

Four measures were utilized to evaluate the burden of disease in basic services: incidence, prevalence, mortality, and disability-adjusted life year rates.^[9]^ We also examined differences in sex and age, utilizing age-standardized rates to control for the influence of age, resulting in a unified metric for comparison that ensures consistency across most rates. Disability-adjusted life years refer to the years of healthy life lost due to both premature death and disability. It is a comprehensive measure of the quantity and quality of life over time. In burden of disease studies, sociodemographic index (SDI) is a comprehensive indicator utilized to evaluate the developmental status of a country or region. These indexes typically encompass various factors, such as the overall fertility rate among women under 25 years old, the average educational attainment level among individuals aged 15 years and above, and per capita income. Ranging from 0 to 1, SDI values reflect higher levels of social development as they increase.^[7,9]^

### 2.3. Grouped by age and region

In this study, we conducted an analysis of dementia data from 204 countries and 21 GBD regions encompassing geographically adjacent countries with similar epidemiological characteristics. The analysis included multiple age groups ranging from 40–44 to ≥ 95 years. Additionally, SDI was calculated for each country to evaluate the association between dementia burden and socioeconomic development. All 204 countries and regions were categorized into 5 SDI groups: high SDI (>0.81), high-middle SDI (0.7–0.81), middle SDI (0.61–0.70), low-middle SDI (0.46–0.61), and low SDI (<0.46). This index serves as a reflection of a country or region’s socioeconomic development level, population structure, education level, and other key factors that are crucial in comprehending the distribution and changes in disease burden.

### 2.4. Statistical analysis

The age-standardized prevalence, age-standardized incidence, age-standardized mortality, and age-standardized disability-adjusted life year rates of dementia were compared across different countries, regions, age groups, and genders. Age-standardized rates are calculated per 100,000 people with the following formula: $Age\text{-}standardised\;\;\;rates\;\;\;=\;\;\;\frac{\sum_{i=1}^{A}a_{i}w_{i}}{\sum_{i=1}^{A}w_{i}}\;\;\;\times 100,000$, where a_i_ and w_i_ denote age-specific rates and the number of persons (or weight) in the same age subgroup of the chosen reference standard population (where i denotes the ith age class), respectively. The average annual percentage change (AAPC) was estimated using generalized linear regression models to calculate the mean annual change in age-standardized rates. The equation Y = α + βX + ε was employed to establish the relationship between the natural logarithm (ln) of age-standardized rates and time, thereby capturing the temporal evolution pattern of age-standardized rates. An increasing or decreasing trend was identified if both AAPC and 95% confidence interval (CI) were greater than or less than zero, respectively. Statistical analysis was conducted using version 4.3.0 of the R statistical package.^[10]^

## 3. Results

### 3.1. Global level

**Figure 1.**
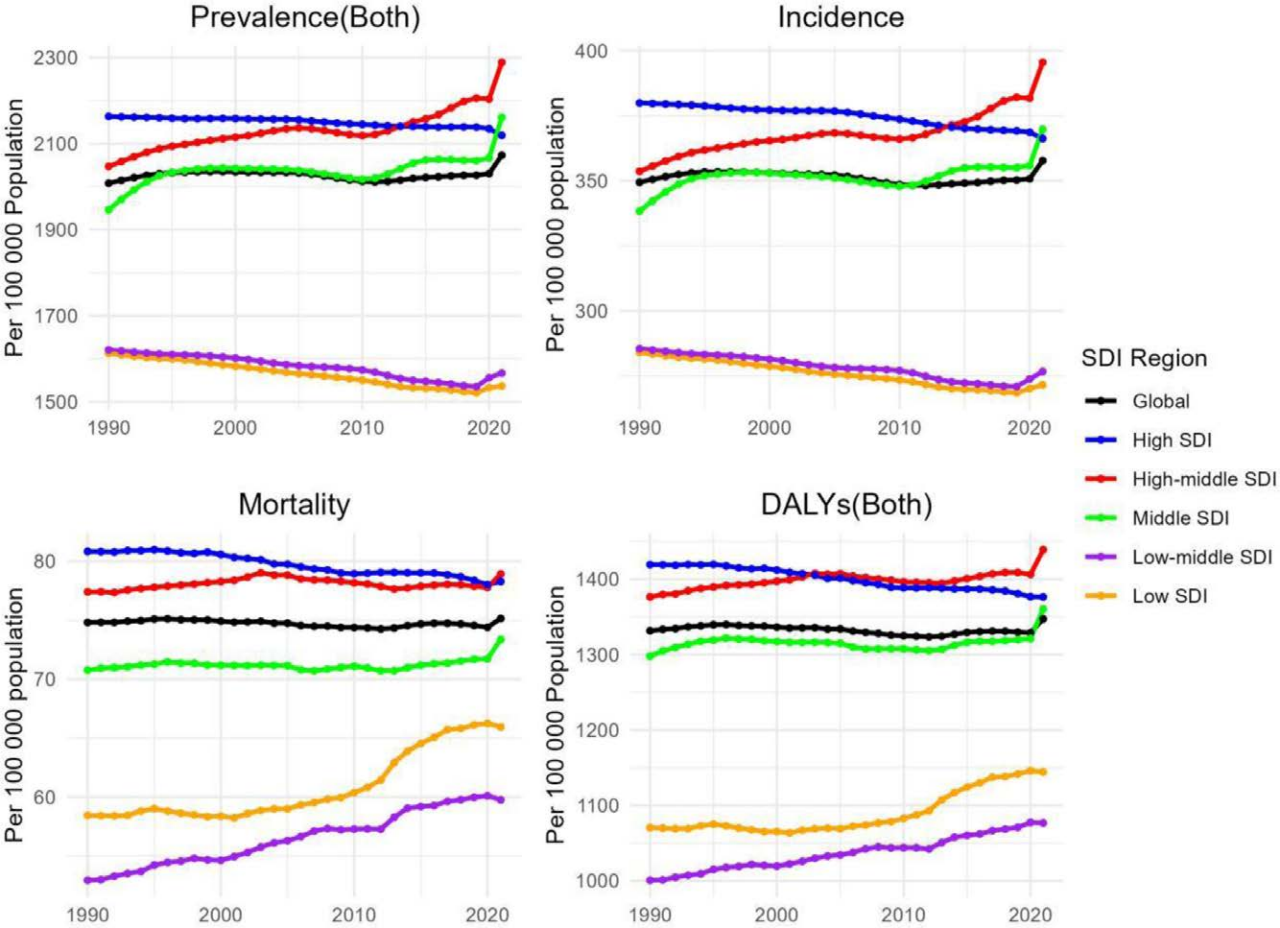
Trends in dementias prevalence, incidence, mortality, and disability-adjusted life-years from 1990 to 2021. DALY = disability-adjusted life year rate, SDI = sociodemographic index.

In recent years, the situation of dementia patients worldwide has displayed a notable changing trend, as shown in Figure 1. As indicated in Table 1, the overall prevalence of dementia in 2019 reached 218.00 × 10^6^ (95% uncertainty interval [UI]: 171.93 × 10^6^–272.74 × 10^6^). By 2021, the prevalence increased by 160.81%, and the number of patients amounted to 568.57 × 10^6^ (95% UI: 445.43 × 10^6^–713.3 × 10^6^). Simultaneously, the age-standardized prevalence rate also presented an increasing trend, from 2008.15/100,000 in 1990 (95% UI: 1588.09–2505.26) to 2073.23/100,000 in 2021 (95% UI: 1625.49–2599.49), with an AAPC of 0.09 (95% CI: 0.08–0.1). Globally, the incidence of dementia is steadily escalating, rising from 38.35 × 10^6^ (95% UI: 26.42 × 10^6^–52.05 × 10^6^) cases in 1990 to 98.37 × 10^6^ cases in 2021 (95% UI: 67.13 × 10^6^–134.25 × 10^6^), and the AAPC was 0.06 (95% CI: 0.05–0.07). However, although the number of deaths attributed to dementia increased from 6.63 × 10^6^ in 1990 (95% UI: 1.58 × 10^6^–18.70 × 10^6^) to 19.53 × 10^6^ in 2021 (95% UI: 4.86 × 10^6^–52.00 × 10^6^), the age-standardized mortality rate did not undergo a significant alteration (the AAPC = 0; 95% CI: −0.02 to 0.03). Additionally, the disability-adjusted life year rates globally ascended from 135.72 × 10^6^ (95% UI: 62.18 × 10^6^–303.65 × 10^6^) in 1990 to 363.33 × 10^6^ (95% UI: 166.04 × 10^6^–800.36 × 10^6^), but the AAPC was merely 0.03 (95% CI: −0.01 to 0.05), suggesting that the change was insignificant.

**Table 1:** Global prevalence, incidence, mortality, and disability-adjusted life year rates of dementias from 1990 to 2021

Year	Both	Male	Female
1990			
Prevalence cases (10^6^) (95% UI)	218.00 (171.93–272.74)	76.56 (59.64–96.60)	141.43 (112.09–176.28)
Incidence cases (10^6^) (95% UI)	38.35 (26.42–52.05)	13.52 (9.18–18.56)	24.82 (17.24–33.53)
Mortality cases (10^6^) (95% UI)	6.63 (1.58–18.70)	2.00 (0.46–5.74)	4.63 (1.12–12.54)
DALYs (10^6^) (95% UI)	135.72 (62.18–303.65)	44.66 (20.16–103.48)	91.07 (41.67–200.87)
ASPR (per 100,000) (95% UI)	2008.15 (1588.09–2505.26)	1707.16 (1336.75–2144.39)	2199.11 (1743.96–2737.67)
ASIR (per 100,000) (95% UI)	349.44 (242.31–472.85)	300.8 (206.19–410.36	381.84 (265.9–515.41)
ASMR (per 100,000) (95% UI)	74.81 (17.98–203.21)	60.28 (14.03–169.85)	82.45 (20.04–220.95
ASDR (per 100,000) (95% UI)	1331.59 (601.07–2974.82)	1084.37 (481.56–2518.91)	1478.87 (669.65–3257.91)
2021			
Prevalence cases (10^6^) (95% UI)	568.57 (445.43–713.34)	207.53 (160.69–262.04)	361.03 (284.43–451.93)
Incidence cases (10^6^) (95% UI)	98.37 (67.13–134.25)	36.45 (24.65–50.08)	61.92 (42.50–84.30.
Mortality cases (95% UI)	19.53 (4.86–52.00)	6.27 (1.48–17.73)	13.26 (3.37–34.32)
DALYs (95% UI)	363.33 (166.04–800.36)	125.24 (56.69–288.18)	238.09 (109.39–511.26)
ASPR/100,000 persons (95% UI)	2073.23 (1625.49–2599.49)	1760.95 (1366.12–2220.37)	2300.05 (1811.7–2879.46)
ASIR/100,000 persons (95% UI)	357.76 (244.41–487.7)	308.88 (209.56–423.4)	395.2 (271.11–538.09)
ASMR/100,000 persons (95% UI)	75.15 (18.73–199.22)	61.85 (14.68–172.99	83.28 (21.15–215.85)
ASDR/100,000 persons (95% UI)	1347.24 (613.41–2963.78)	1112.87 (498.5–2561.53)	1508.22 (693.7–3239.45)
1990-2021			
Prevalence (%)	160.81	171.06	155.27
Incidence (%)	156.54	169.57	149.44
Mortality (%)	194.39	212.84	186.41
DALYs (%)	167.7	180.46	161.44
EAPC of ASPR (95% CI)	0.09 (0.08–0.1)	0.09 (0.05,0.13)	0.13 (0.11,0.15)
EAPC of ASIR (95% CI)	0.06 (0.05–0.07)	0.07 (0.05–0.1)	0.1 (0.08–0.11)
EAPC of ASMR (95% CI)	0 (−0.02 to 0.03)	0.08 (0.05 to 0.11)	0.02 (0 to 0.04)
EAPC of ASDR (95% CI)	0.03 (0.01–0.05)	0.07 (0.04–0.1)	0.05 (0.03–0.07)

AAPC = average annual percentage change, ASDR = age-standardized disability-adjusted life-year rate, ASIR = age-standardized incidence rate, ASMR = age-standardized mortality rate, ASPR = age-standardized prevalence rate, CI = confidence interval, DALYs = disability-adjusted life-years, EAPC = estimated annual percentage change, UI = uncertainty interval.

### 3.2. Regional level

**Figure 2.**
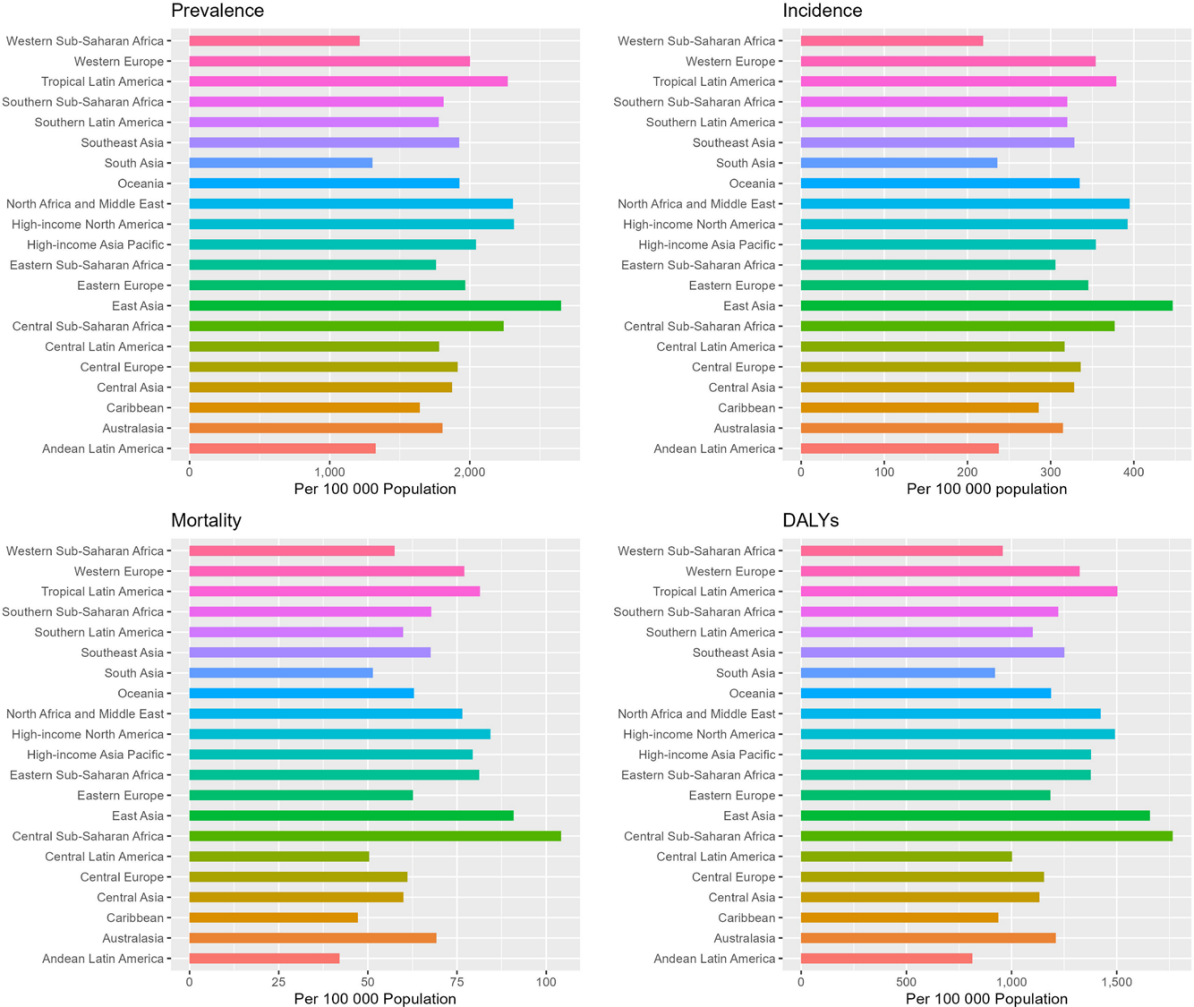
Regional age-standardized prevalence rate, age-standardized incidence rate, age-standardized mortality rate, and age-standardized disability-adjusted life-year rate of dementias in 2021. DALY = disability-adjusted life year rate.

**Figure 3.**
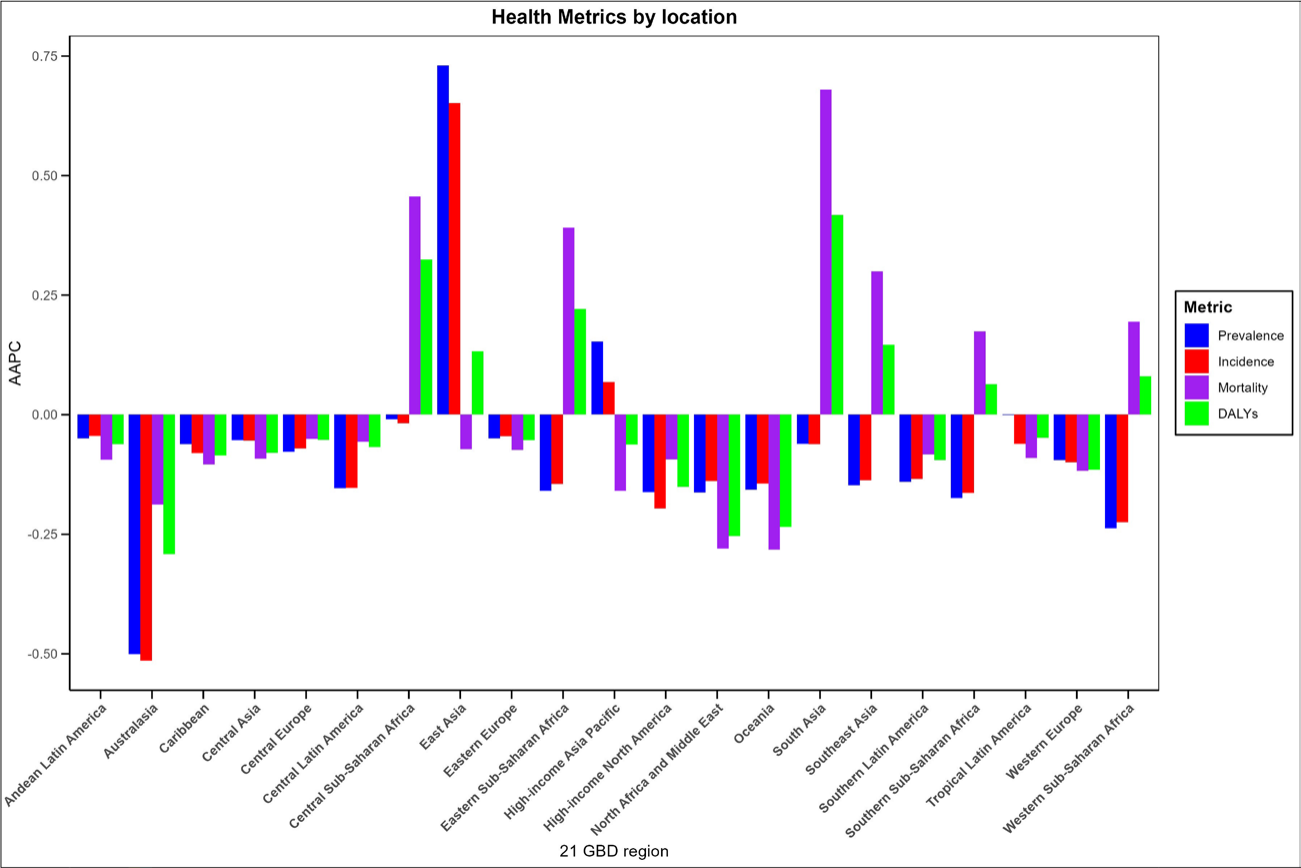
The average annual percentage change (AAPC) of age-standardized prevalence rate, age-standardized incidence rate, age-standardized mortality rate, and age-standardized disability-adjusted life-year rate of dementias in 21 Global Burden of Disease (GBD) regions from 1990 to 2021. DALY = disability-adjusted life year rate.

In 2021, significant regional disparities were observed in the prevalence of dementia across various regions worldwide. The 3 regions with the highest prevalence were East Asia (1.74 million, 95% UI: 1.35–2.20), Western Europe (0.77 million, 95% UI: 0.61–0.96), and high-income North America (0.55 million, 95% UI: 0.43–0.69). Furthermore, the age-standardized prevalence rate was analyzed. The results presented in Figure 2 reveal that East Asia has the highest age-standardized prevalence rate, followed by high-income North America and North Africa and the Middle East. Figure 3 provides a superior visualization of the trends in dementia prevalence by region from 1990 to 2021. The age-standardized prevalence rates of dementia are statistically decreasing (the AAPC < 0) in all regions except for East Asia (the AAPC = 0.73, 95% CI: 0.68–0.78) and high-income Asia-Pacific countries (the AAPC = 0.15, 95% CI: 0.13–0.18), which show an increasing trend. The Oceania region exhibits the most significant decrease, with an AAPC value of −0.5 (95% CI: −0.52 to −0.48). In 2021, the 3 regions with the highest age-standardized prevalence rate worldwide, in descending order of magnitude, were East Asia (446.94/100,000 population, 95% CI: 305.81–612.673), North Africa and the Middle East (394.894/100,000 population, 95% CI: 269.747–539.024), and high-income North America (392.5/100,000 population, 95% CI: 270.532–532.759). From 1990 to 2021, a significant growth trend in the incidence was observed in East Asia, with the greatest age-standardized prevalence rate being 0.65 (95% CI: 0.51–0.79). Meanwhile, a marginal increase was also noted in the incidence in high-income countries within the Asia-Pacific region (the AAPC = 0.07, 95% CI: 0.04–0.09). In contrast, the incidence rates in other regions demonstrated an overall downward trend (the AAPC < 0). In 2021, significant regional differences were manifested in global dementia mortality. Specifically, the 3 regions with the highest mortality rates were Eastern Asia (0.51 million, 95% UI: 0.12–1.39), Western Europe (0.34 million, 95% UI: 0.09–0.86), and high-income North America (0.22 million, 95% UI: 0.06–0.57). Regarding the age-standardized mortality rate, the highest was in Central sub-Saharan Africa, followed by East Asia, while high-income North America ranked third. Upon further analysis of the change trend by region, it was discovered that the most significant increase in age-standardized mortality rate was witnessed in South Asia, with an AAPC of 0.68 (95% CI: 0.5–0.86). Additionally, the age-standardized mortality rate of Central Sub-Saharan Africa, Eastern Sub-Saharan Africa, Southeast Asia, Western Sub-Saharan Africa, and Southern Sub-Saharan Africa also exhibited an upward trend, while the age-standardized mortality rate of all other regions presented a downward trend (the AAPC < 0). In 2021, significant regional variations were evident in the global disability-adjusted life year rates associated with dementia. The highest disability-adjusted life year rates were noted in Central Sub-Saharan Africa (1766.73/100,000, 95% UI: 731.02–4174.95), followed by East Asia, while Tropical Latin America ranked third. Regarding the overall trend, Figure 3 suggests that the disability-adjusted life year rates in the following 6 regions display an increasing trend: South Asia, Central Sub-Saharan Africa, Eastern Sub-Saharan Africa, Southeast Asia, East Asia, and Western and Southern Sub-Saharan Africa. Among them, the growth trend in South Asia was the most pronounced (the AAPC = 0.22, 95% CI: 0.2–0.24). In contrast, the disability-adjusted life year rates in the other 15 regions all show a downward trend, with the most significant decline witnessed in Oceania (the AAPC = −0.29, 95% CI: −0.31 to −0.27). Overall, the disease burden of dementia is substantial in all regions worldwide, with notable geographical disparities.

### 3.3. Burden of dementias based on SDI

**Figure 4.**
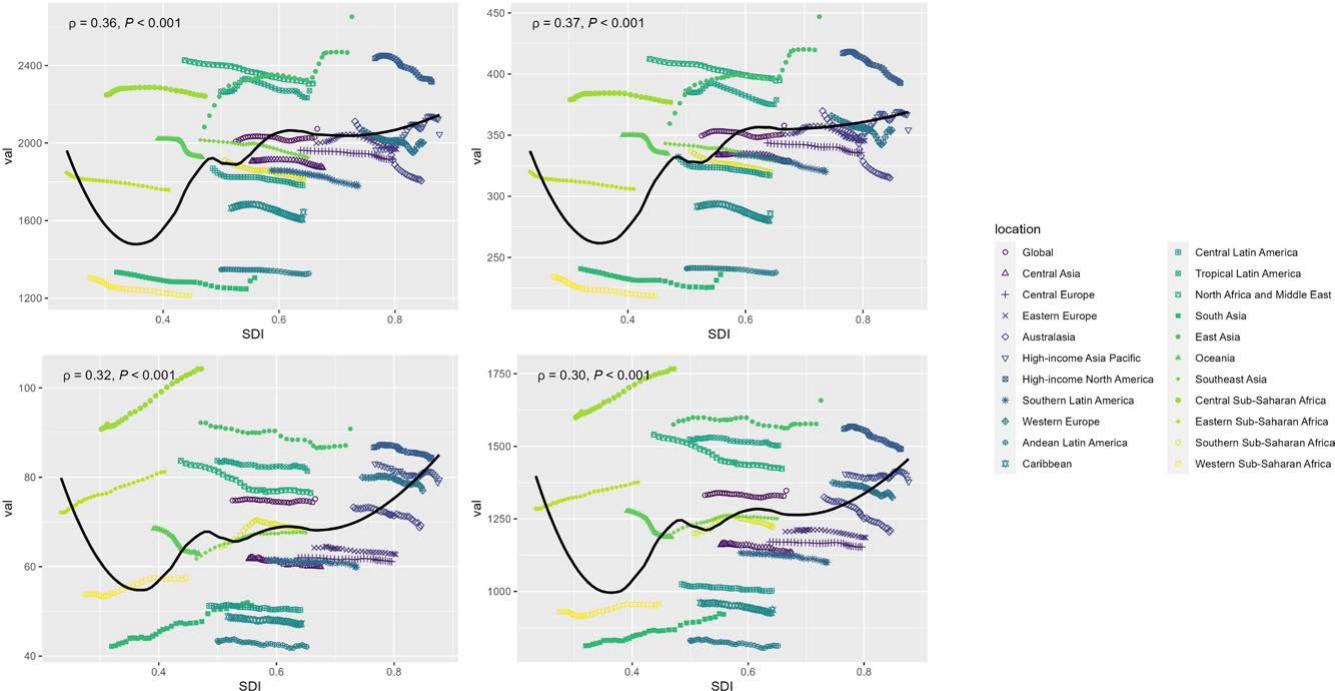
Age-standardized burden rate attributable to dementias across 21 Global Burden of Disease regions ranked by sociodemographic index (SDI) for both sexes, 1990–2019. The black line was an adaptive association fitted with adaptive Loess regression based on all data points. ASDR = age-standardized disability-adjusted life-year rate, ASIR = age-standardized incidence rate, ASMR = age-standardized mortality rate, ASPR = age-standardized prevalence rate.

**Figure 5.**
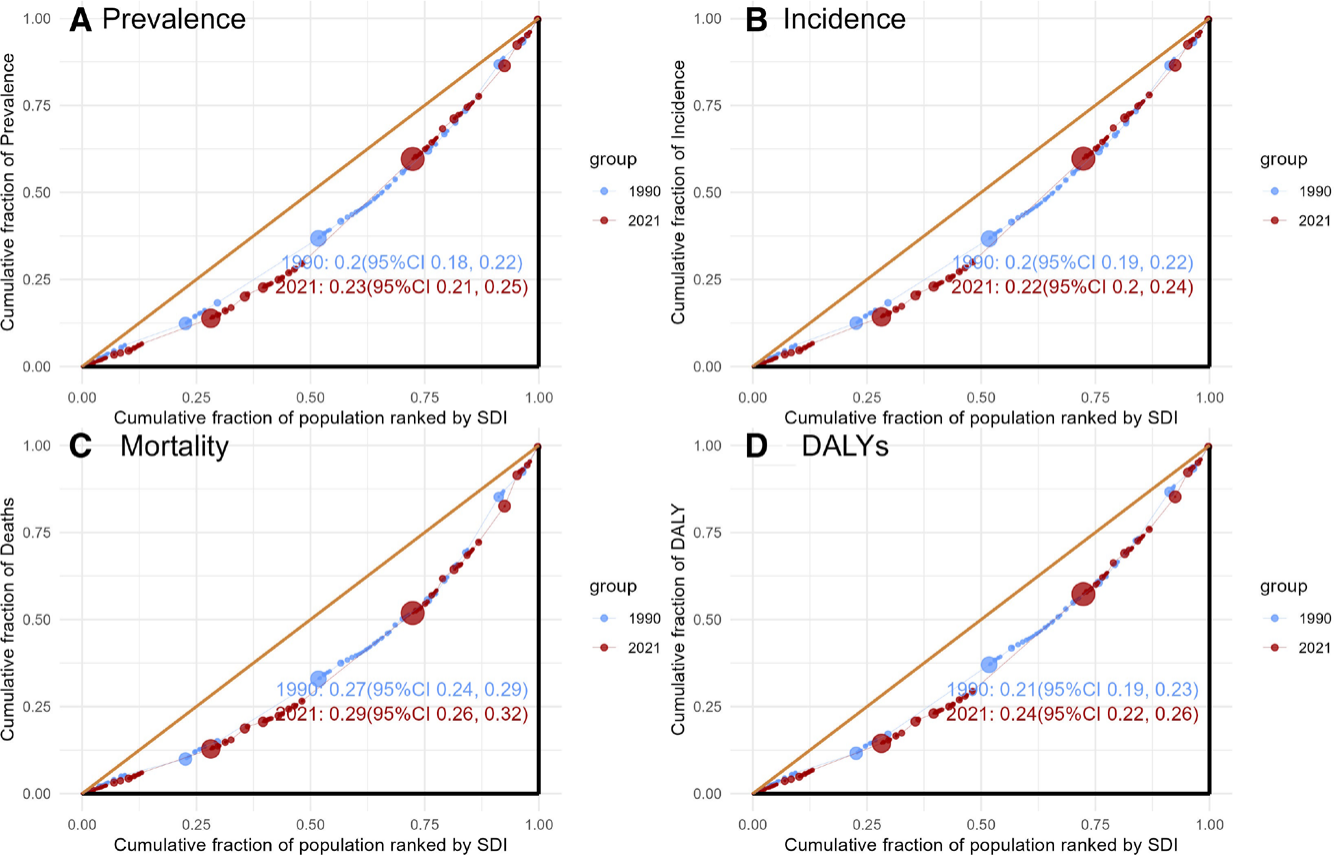
Cumulative fraction of population ranker by sociodemographic index (SDI) in 1990 and 2021. (A) Cumulative fraction of age-standardized prevalence rate. (B) Cumulative fraction of age-standardized incidence rate. (C) Cumulative fraction of age-standardized mortality rate. (D) Cumulative fraction of age-standardized disability-adjusted life-year rate. CI = confidence interval, DALY = disability-adjusted life year rate.

SDI is an important indicator of socioeconomic status and population health. In 2021, the prevalence and incidence of dementia worldwide were mainly concentrated in middle SDI, high-middle SDI, and high SDI areas. As indicated in Table 2, the global age-standardized prevalence rate demonstrated an increasing trend (the AAPC = 0.09, 95% CI: 0.08–0.1). The increase trend was particularly notable in the middle SDI (the AAPC = 0.31, 95% CI: 0.22–0.39) and the high-middle SDI (the AAPC = 0.32, 95% CI: 0.27–0.37) areas. Simultaneously, the global age-standardized incidence rate also rose marginally (the AAPC = 0.06, 95% CI: 0.05–0.07). The increase was also concentrated in the middle SDI (the AAPC = 0.26, 95% CI: 0.19–0.33) and the high-middle SDI (the AAPC = 0.32, 95% CI: 0.27–0.38). However, there was no significant increase in age-standardized mortality rate worldwide according to Table 2, with an AAPC of 0 (95% CI: −0.02 to 0.03). Nevertheless, regions characterized by low and low-middle SDI exhibited an upward trend in the age-standardized mortality rate, with AAPCs of 0.41 (95% CI: 0.32–0.5) and 0.41 (95% CI: 0.31–0.52), respectively. In terms of the disability-adjusted life year rates, the High SDI areas exhibited a declining trend, characterized by an AAPCs of −0.12 (95% CI: −0.13 to −0.11). Conversely, the other regions and the global population as a whole demonstrated an upward trend, with an AAPC of 0.06 (95% CI: 0.05–0.07). Notably, among these regions, the low-middle SDI area displayed the most significant upward trend, with an AAPC of 0.24 (95% CI: 0.19–0.3). The changing trend of each age-standardized indicator in 21 regions of the world from 1990 to 2021, as well as the standard rate prediction curves for regions with different SDI, are illustrated in Figure 4. The concentration index presented in Figure 5 primarily examines the distribution of dementia across various SDI regions. In 1990, the concentration index for each indicator in different SDI regions exceeded 0.2 and further increased by 2021, indicating an escalated concentration of disease burden within specific regions and significant regional or group disparities, thereby highlighting existing health inequalities.

**Table 2:** Average annual percentage change of age-standardized prevalence rate, age-standardized incidence rate, age-standardized mortality rate, and age-standardized disability-adjusted life-year rate for dementias in countries with 5 sociodemographic index levels from 1990 to 2021.

Region	ASPR/(10^6^) (95% UI)	AAPC of ASPR	ASIR/(10^6^) (95% UI)	AAPC of ASIR	ASMR/(10^6^) (95% UI)	AAPC of ASMR	ASDR/(10^6^) (95% UI)	AAPC of ASDR
	(1990/2021)	(95% UI)	(1990/2021)	(95% UI)	(1990/2021)	(95% UI)	(1990/2021)	(95% UI)
Global	2008.15 (1588.09–2505.26)/2073.23 (1625.49–2599.49)	0.09 (0.08 to 0.1)	349.44 (242.31–472.85)/357.76 (244.41–487.7)	0.06 (0.05 to 0.07)	74.81 (17.98–203.21)/75.15 (18.73–199.22)	0 (−0.02 to 0.03)	1331.59 (601.07–2974.82)/1347.24 (613.41–2963.78)	0.03 (0.01 to 0.05)
Low SDI	1612.38 (1252.76–2031.32)/1536.59 (1199.09–1935.47)	−0.15 (−0.17 to −0.13)	284.02 (192.45–390.07)/271.51 (183.78–371.23)	−0.14 (−0.16 to −0.13)	58.44 (13.25–167.58)/65.94 (15.27–185.1)	0.41 (0.32 to 0.5)	1070.41 (466.97–2503.52)/1144.26 (482.67–2713.75)	0.23 (0.19 to 0.26)
Low-middle SDI	1620.55 (1264.12–2042.41)/1566.77 (1220.95–1979.75)	−0.1 (−0.12 to −0.08)	285.45 (193.75–391.22)/276.67 (187.09–379.67)	−0.1 (−0.11 to −0.08)	52.94 (12.16–151.05)/59.76 (14.1–166.08)	0.41 (0.31 to 0.52)	1000.7 (452.99–2300.68)/1076.62 (470.63–2480.12)	0.24 (0.19 to 0.3)
Middle SDI	1946.14 (1516.1–2455.34)/2161.09 (1683.97–2726.46)	0.31 (0.22 to 0.39)	338.36 (229.4–464.12)/369.81 (251.5–508.21)	0.26 (0.19 to 0.33)	70.76 (16.43–196.05)/73.37 (17.83–198.45)	0.1 (0.07 to 0.14)	1297.77 (577.45–2950.41)/1360.28 (621.39–3014.36)	0.13 (0.1 to 0.16)
High-middle SDI	2047.16 (1608.8–2562.79)/2288.89 (1784.01–2883.63)	0.32 (0.27 to 0.37)	353.64 (243.14–481.67)/395.52 (269.04–541.41)	0.32 (0.27 to 0.38)	77.41 (18.47–211.85)/78.93 (19.31–213.62)	0.05 (0.01 to 0.08)	1376.52 (615.92–3100.63)/1439 (661.15–3165.42)	0.1 (0.06 to 0.15)
High SDI	2163.45 (1728.99–2673.95)/2119.4 (1675.22–2637.84)	−0.06 (−0.07 to −0.05)	379.94 (268.04–507.61)/366.27 (254.22–494.63)	−0.12 (−0.13 to −0.11)	80.84 (19.99–215.51)/78.29 (20.2–199.71)	−0.11 (−0.14 to −0.09)	1419.35 (652.11–3104.72)/1376.42 (639.44–2920.57)	−0.1 (−0.12 to −0.09)

AAPC = average annual percentage change, ASDR = age-standardized disability-adjusted life-year rate, ASIR = age-standardized incidence rate, ASMR = age-standardized mortality rate, ASPR = age-standardized prevalence rate, CI = confidence interval, SDI = sociodemographic index, UI = uncertainty interval.

### 3.4. National level

**Figure 6.**
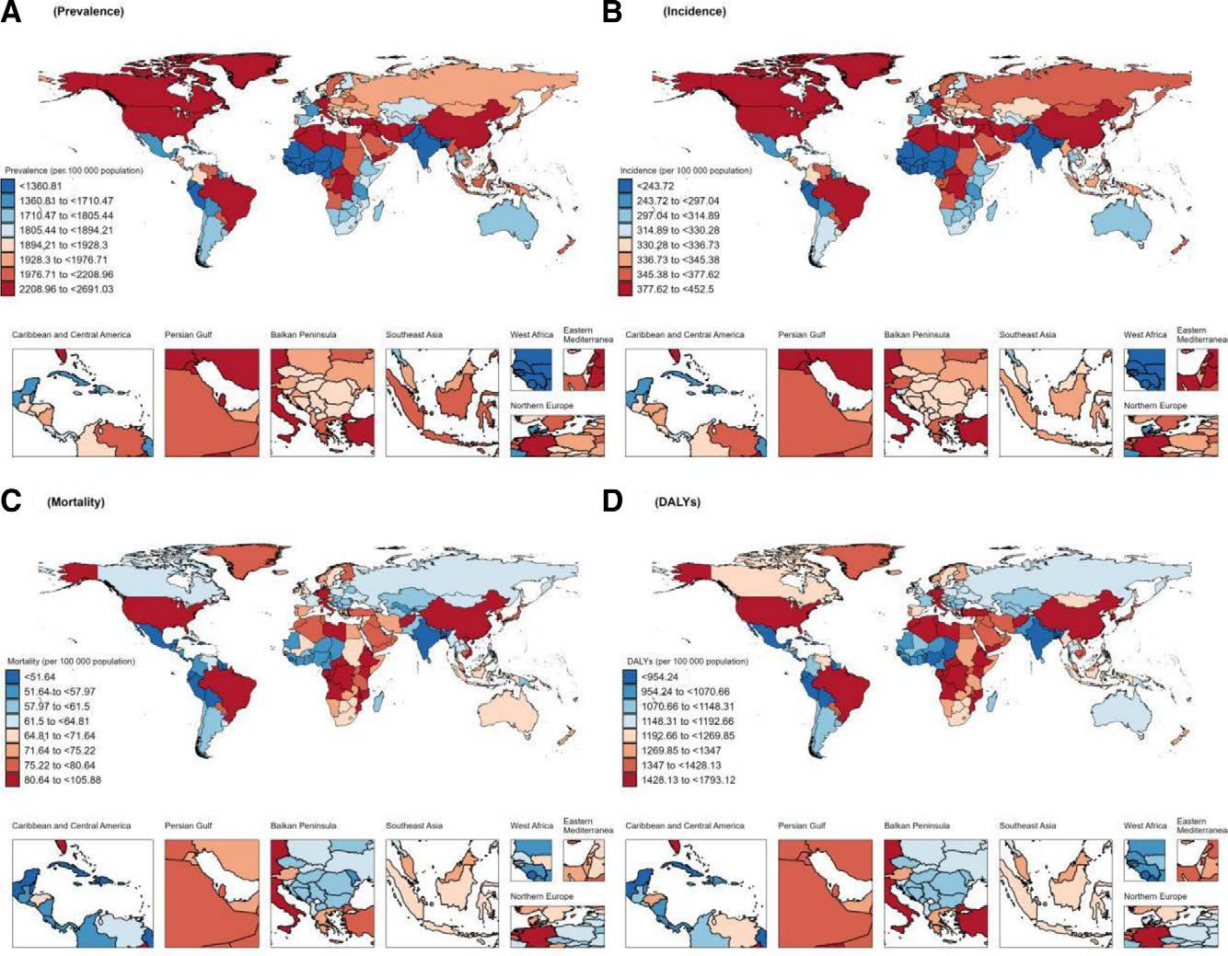
The global disease burden of dementias for both sexes in 204 countries and territories in 2021. (A) Prevalence, (B) incidence, (C) mortality, and (D) disability-adjusted life-years (DALYs).

**Figure 7.**
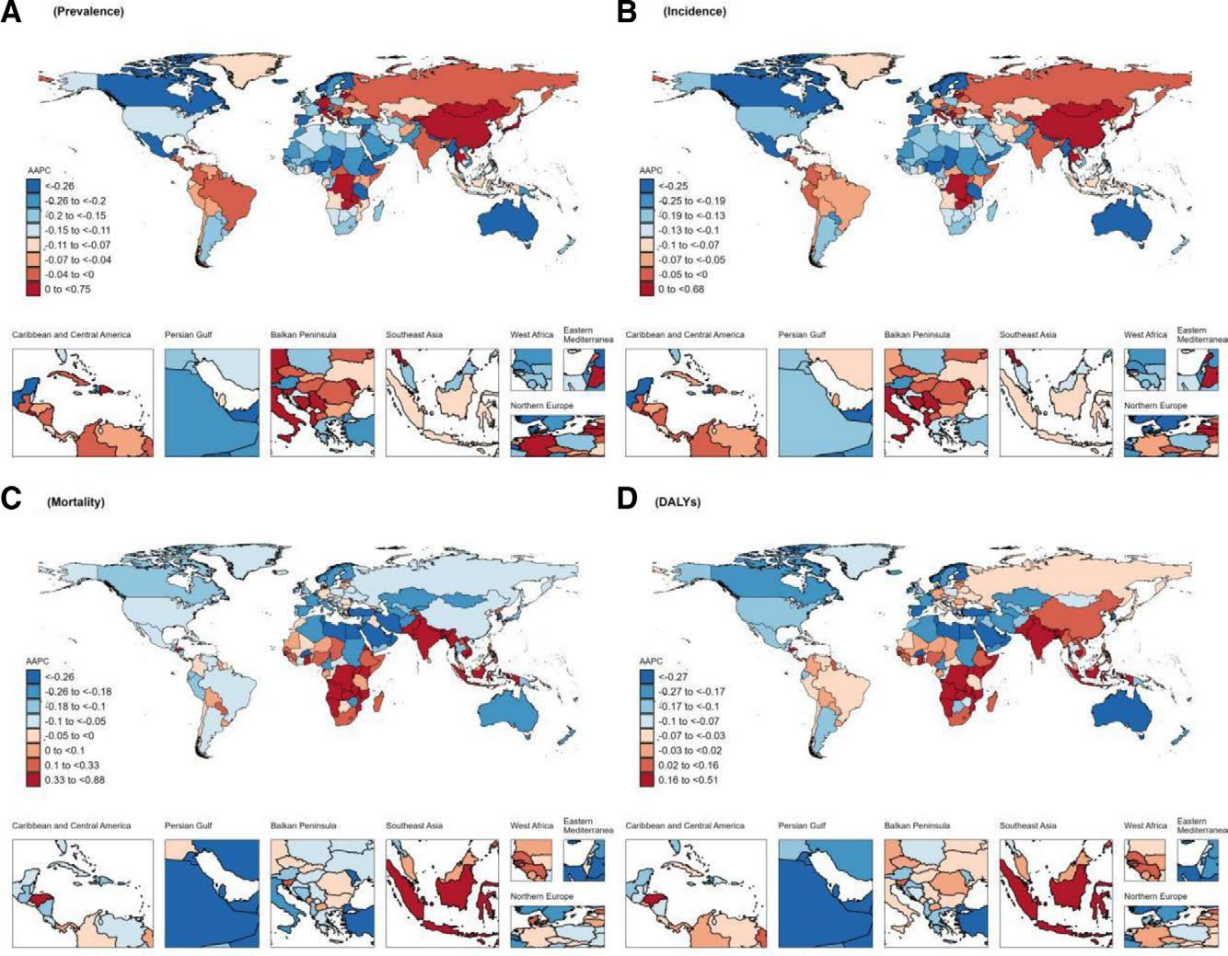
The global disease burden of dementias for both sexes in 204 countries and territories from 1990 to 2021. (A) Average annual percentage change (AAPC) of age-standardized prevalence rate. (B) AAPC of age-standardized incidence rate. (C) AAPC of age-standardized mortality rate. (D) AAPC of age-standardized disability-adjusted life-year rate. DALY = disability-adjusted life-year.

The countries and specific regions with the highest prevalence of dementia worldwide in 2019, as indicated in Tables 1–3, Supplemental Digital Content, http://links.lww.com/MD/O485, encompass China, the United States of America, and India. China accounted for 4.02 million cases (95% UI: 3.1–5.12). In the same year, the 3 countries with the highest age-standardized prevalence rates were Canada, Turkey, and Yemen. As of 2021, as depicted in Figure 6, China’s age-standardized prevalence rate rapidly rose to the top position, attaining 2691.03/100,000 individuals (95% UI: 2087.16–3395.2), and emerged as the country with the highest AAPC in Figure 7 (the AAPC = 0.68, 95% CI: 0.63–0.72). In contrast, countries such as Norway, Australia, and Denmark showed a downward trend in age-standardized prevalence rate. When observing the trends in age-standardized incidence rate from 2019 to 2021, it can be noticed that significant changes occurred in the rankings of several key nations. In 2019, Canada, Turkey, and Germany were the top 3 countries in terms of age-standardized incidence rate, with rates of 582.48/100,000 (95% UI: 324.46–582.48), 301.517/100,000 (95% UI: 301.52–598.5), and 433.52/100,000 (95% UI: 318.18–564.85), respectively. By 2021, however, this ranking underwent significant modifications, with China, Germany, and Lebanon emerging as the 3 nations with the highest incidence rates, at 452.5/100,000 individuals (95% UI: 309.41–620.8), 424.59/100,000 individuals (95% UI: 303.77–563.55), and 419.67/100,000 individuals (95% UI: 286.36–573.64), respectively. Additionally, when closely examining the AAPC, as illustrated in Figure 7, China, Italy, and Taiwan Province of China were ranked among the top 3 in terms of the AAPC growth. Notably, China displayed the most significant increase in both prevalence and incidence, a trend that merits in-depth study and attention. We also observed significant disparities in the number of deaths and age-standardized mortality rate among countries. According to the latest data, the top 3 countries with the highest number of deaths in 2019 were China, the United States of America, and Germany, with estimated deaths of 0.12 million (95% UI: 0.03–0.33), 0.1 million (95% UI: 0.02–0.26), and 0.04 million (95% UI: 0.01–0.1), respectively. The top 3 countries in terms of age-standardized mortality rate in 2019 were South Korea, Gabon, and Afghanistan. In 2021, the Democratic Republic of the Congo, Gabon, and Angola comprised the 3 countries with the highest age-standardized mortality rate. It is notable that Gabon remained among the top 3 countries in both years; however, its AAPC was merely 0.02 (95% CI: −0.04 to 0.07), signifying that the trend of dementia mortality in Gabon was relatively stable. In contrast, a considerable number of countries have witnessed more substantial decrements in age-standardized mortality rate. Among them, the AAPCs of Guam, South Korea, and the United Arab Emirates were −0.79 (95% CI: −0.93 to −0.65), −0.68 (95% CI: −0.73 to −0.64), and −0.59 (95% CI: −0.87 to −0.31), respectively, signifying that these countries have made significant progress in controlling dementia mortality. We observe that the top 3 countries in terms of the disability-adjusted life year rates in 2021 are China, the United States of America, and India, with estimated disability-adjusted life year rates of 10,072,477.5 (95% UI: 4,689,419.14–22,121,858.66), 3,318,642.62 (95% UI: 1,501,280.15–7,191,208.66), and 2,787,339.06 (95% UI: 1,207,336.56–6,538,75). These figures reflect the considerable impact dementia has on global population health. When comparisons of age-standardized disability-adjusted life year rates were made, the Democratic Republic of Congo, Gabon, and Afghanistan had the highest age-standardized disability-adjusted life year rates. Further analyzing the trend of age-standardized disability-adjusted life year rates, we found that India, Indonesia, and Uganda had the most rapidly increasing age-standardized disability-adjusted life year rates, with AAPCs of 0.51 (95% CI: 0.4–0.63), 0.47 (95% CI: 0.44–0.5), and 0.45 (95% CI: 0.41–0.5), respectively. This implies that the health burden of dementia in these countries may increase further in the future. In contrast, the United Arab Emirates, South Korea, and Guam witnessed the most remarkable age-standardized disability-adjusted life year rate declines, with AAPCs of −0.56 (95% CI: −0.76 to −0.37), −0.49 (95% CI: −0.51 to −0.46), and −0.45 (95% CI: −0.54 to −0.36), respectively. The common downward trend of age-standardized disability-adjusted life year rates and age-standardized mortality rates in these 3 countries indicated that these countries have achieved positive outcomes in the prevention and control of dementia.

### 3.5. Burden of dementias based on age and sex

**Figure 8.**
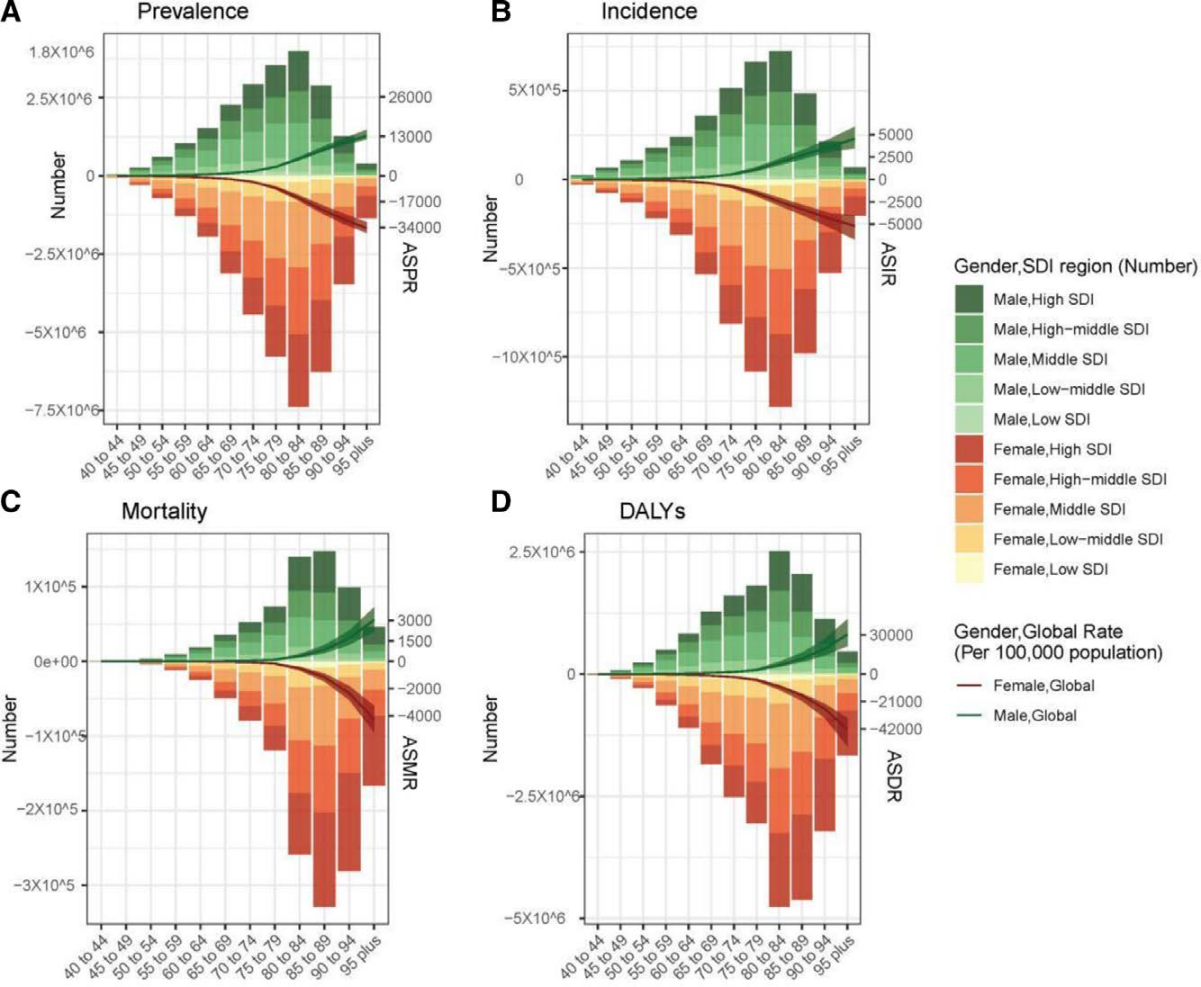
The age-specific numbers and age-standardized prevalence rates (ASPRs) of dementias based by sociodemographic index (SDI) regions in 2021. (A) Prevalence, (B) incidence, (C) mortality, and (D) disability-adjusted life-years (DALYs). ASDR = age-standardized disability-adjusted life-year rate, ASIR = age-standardized incidence rate, ASMR = age-standardized mortality rate.

Figure 8 provides a comprehensive depiction of the disease burden across different age groups globally and in all SDI regions. Globally, there has been an upward trend in the age-standardized prevalence rate from 1990 to 2021, with the highest prevalence observed among individuals aged >95 years. Notably, the age group of 65 to 69 years exhibited the most significant AAPC (0.23, 95% CI: 0.22–0.25). Furthermore, not only did females have a higher age-standardized prevalence rate compared to males, but they also demonstrated a more pronounced annual increase trend with an AAPC of 0.13 (95% CI: 0.11–0.15). In terms of incidence, the age-standardized incidence rate exhibited a consistent increase with advancing age. Notably, the age groups 55 to 59, 60 to 64, and 65 to 69 years demonstrated the highest annual growth rates, with AAPCs of 0.26 (95% CI: 0.22–0.31), 0.25 (95% CI: 0.22–0.28), and 0.26 (95% CI: 0.22–0.31), respectively, indicating a gradual expansion in the incidence of dementia among younger individuals over time. The age-standardized mortality rate and age-standardized disability-adjusted life year rates also exhibited an upward trend with increasing age, peaking in the 95+ years age group. Although the rise in mortality rates across different age groups was relatively gradual, the highest AAPC of 0.08 (95% CI: 0.04–0.11) was observed in the 40 to 44 years age group, potentially attributed to the diverse range of dementia causes within this cohort. Furthermore, there consistently existed a higher burden of disease among women compared to men; however, men demonstrated more pronounced increases in age-standardized mortality rate and age-standardized disability-adjusted life year rates.

### 3.6. Risk factors

**Figure 9.**
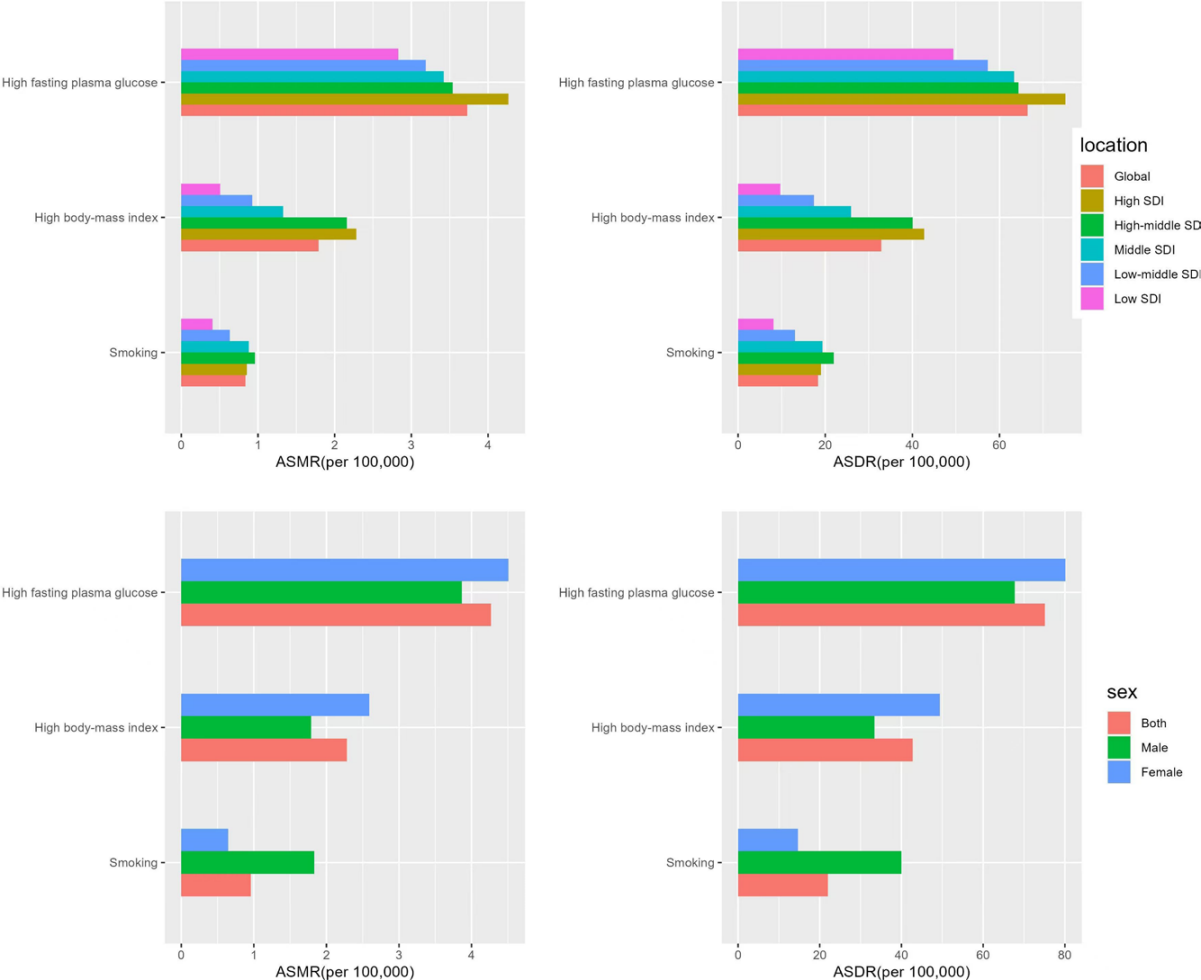
Impact of risk factors on the disease burden of Alzheimer disease by sociodemographic index (SDI) regions and gender in 2021. ASDR = age-standardized disability-adjusted life-year rate, ASMR = age-standardized mortality rate.

Based on the statistical analysis of the GBD2021 database, high fasting blood glucose, high body mass index (BMI), and smoking have been recognized as the primary risk factors for dementia. Globally, as shown in Table 3, high fasting glucose was the leading risk factor, with 290,032 global deaths and 5,348,854 disability-adjusted life year rates in 2021. It is notable that the age-standardized mortality rate and age-standardized disability-adjusted life year rates caused by high fasting blood glucose in Figure 9 were positively correlated with SDI values and peaked in the high SDI area. High BMI followed closely, and its influence on the risk of dementia could not be ignored. High BMI also presented a positive correlation with SDI, with 61,559 deaths and 1,060,113 disability-adjusted life year rates in high SDI areas in 2021. When considering the impact of smoking in different regions, the study found that the disease burden was most prominent in high SDI regions, with 21,771 deaths and 442,915 disability-adjusted life year rates in 2021. This phenomenon might be related to the healthcare conditions and smoking habits in the region. Regarding gender differences, males are more affected by behavioral factors such as smoking, while females are more impacted by metabolic factors such as high BMI. In the population-attributable ratio analysis presented in Table 4, the effective control of male smoking would lead to a reduction of approximately 6.92% in the mortality rate of men with dementia in 2021, while for women, this reduction would merely be 1.55%. After adjusting for high BMI, the mortality reduction was slightly higher in women (7.25%) compared to men (5.91%). The control of fasting hyperglycemia had a substantial impact on the mortality of both men and women with dementia, resulting in reductions of 15.63% and 14.32%, respectively. Furthermore, from 1990 to 2021, the proportion of the dementia burden attributed to smoking decreased from 4.32% to 3.31%, and the disability-adjusted life year rates attributable to the fraction also declined from 5.25% to 4.08%. However, an upward trend was observed in the attributable fractions for fasting hyperglycemia and high BMI, indicating that these 2 factors are increasingly contributing to the escalating burden associated with dementia.

**Table 3:** Main risk factors for dementias related mortality case, disability-adjusted life-years, and average annual percentage change, among people aged ≥ 40 years, 1990–2021.

Risk factors by SDI	Mortality cases (95% UI) (1990/2021)			AAPC (95% CI)	DALYs (95% UI) (1990/2021)			AAPC (95% CI)
	Both	Males	Female		Both	Males	Females	
High fasting plasma glucose								
Global	71,471/290,032	23,067/98,900	48,403/191,131	1.13 (1.08 to 1.18)	1,441,540/5,348,854	497,842/1,929,129	943,698/3,419,725	1.13 (1.1 to 1.16)
High SDI	30,545/116,115	8886/38,734	21,659/77,380	1.28 (1.23 to 1.32)	565,750/1,910,976	178,778/680,390	386,971/1,230,586	1.26 (1.2 to 1.32)
High-middle SDI	17,556/65,959	5182/20,918	12,374/45,041	1.02 (0.95 to 1.08)	357,816/1,237,594	113,145/417,548	244,672/820,046	1.1 (1.05 to 1.15)
Middle SDI	14,869/69,335	5345/24,375	9524/44,959	0.83 (0.76 to 0.89)	327,924/1,411,109	123,053/517,510	204,872/893,599	0.88 (0.84 to 0.92)
Low-middle SDI	6394/30,601	2745/11,674	3649/18,927	1.68 (1.63 to 1.73)	142,662/622,127	62,157/245,474	80,505/376,652	1.53 (1.45 to 1.61)
Low SDI	2025/7757	882/3109	1143/4648	1.27 (1.18 to 1.37)	45,683/162,087	20,113/66,461	25,570/95,626	1.18 (1.11 to 1.26)
High body mass index								
Global	31,577/139,439	6761/38,849	24,816/100,590	1.24 (1.22 to 1.26)	644,750/2,665,746	150,984/784,584	493,766/1,881,162	1.38 (1.36 to 1.4)
High SDI	18,458/61,559	3993/17,838	14,465/43,721	0.82 (0.79 to 0.85)	350,824/1,060,113	85,868/332,293	350,824/1,060,113	0.92 (0.89 to 0.95)
High-middle SDI	9283/40,012	1864/9861	7419/30,151	1.34 (1.31 to 1.37)	199,359/771,425	42,573/201,848	156,786/569,577	1.41 (1.37 to 1.45)
Middle SDI	2462/27,311	596/7934	1865/19,377	3.62 (3.57 to 3.67)	60,674/597,893	15,100/177,559	45,574/420,333	3.71 (3.67 to 3.75)
Low-middle SDI	1150/8921	279/2750	872/6170	3.35 (3.26 to 3.44)	27,918/197,241	6673/27,918	21,246/135,687	3.27 (3.2 to 3.34)
Low SDI	422/1481	16/422	161/1059	4.46 (4.41 to 4.51)	4928/36,100	483/4928	4445/25,644	4.17 (4.14 to 4.21)
Smoking								
Global	32,165/67,176	20,500/46,755	11,665/20,420	−0.84 (−0.87 to −0.82)	794,915/1,533,214	532,606/1,110,023	262,310/423,191	−0.79 (−0.82 to −0.76)
High SDI	13,666/21,771	6850/11,745	6816/11,745	−1.27 (−1.29 to −1.25)	310,666/442,915	162,792/246,852	147,874/196,063	−1.23 (−1.25 to −1.21)
High-middle SDI	7881/18,463	5481/13,472	2399/4990	0.24 (−0.3 to −0.18)	200,798/435,096	145,970/329,224	54,829/105,872	−0.13 (−0.18 to −0.07)
Middle SDI	7296/19,215	5563/15,633	1732/3581	−0.78 (−0.82 to −0.75)	195,493/470,385	154,273/390,315	41,219/80,070	−0.74 (−0.81 to −0.67)
Low-middle SDI	2734/6466	2181/5063	553/1403	−0.52 (−0.59 to −0.45)	71,967/153,657	57,757/122,012	14,210/31,644	−0.64 (−0.68 to −0.6)
Low SDI	560/1216	406/812	153/404	−0.47 (−0.65 to −0.29)	15,215/30,072	11,309/20,918	3906/9153	−0.58 (−0.67 to −0.6)

AAPC = average annual percentage change, CI = confidence interva, DALYs = disability-adjusted life year rates, UI = uncertainty interval.

**Table 4:** Attribution analysis of risk factors in males and females based on SDI regions in 1990 and 2021.

Risk factors by SDI	Both (1990/2021)		Male (1990/2021)		Female (1990/2021)	
	Population (95% UI)	PAF (95% UI)	Population (95% UI)	PAF (95% UI)	Population (95% UI)	PAF (95% UI)
High fasting plasma glucose						
Global	71,470.52 (2849.13 to 221,701.22)/290,032.19 (11,759.52 to 916,714.18)	10.48 (0.89 to 21.03)/14.7 (1.21 to 29.41)	23,067.47 (904 to 74,593.63)/98,900.35 (3914.39 to 316,130.86)	11.31 (0.97 to 22.72)/15.63 (1.29 to 31.59)	48,403.05 (1949.91 to 148,604.25)/191,131.84 (7845.14 to 600,476.66)	10.19 (0.86 to 20.4)/14.32 (1.17 to 28.78)
High SDI	30,545.19 (1283.05 to 95,702.96)/116,114.61 (4820.68 to 364,124.07)	10.57 (0.93 to 20.65)/16.15 (1.33 to 31.95)	8886.11 (368.36 to 28,341.87)/38,734.25 (1576.87 to 124,186.27)	11.73 (1.04 to 23.32)/17.65 (1.48 to 34.71)	21,659.08 (913.46 to 67,725.36)/77,380.36 (3261.58 to 239,139.82)	10.21 (0.89 to 19.97)/15.53 (1.27 to 30.66)
High-middle SDI	17,555.54 (696.59 to 55,919.93)/65,959.34 (2680.76 to 205,256.98)	10.01 (0.84 to 20.14)/13.29 (1.1 to 26.5)	5181.97 (202.66 to 17,193.19)/20,917.96 (845.3 to 67,946.27)	10.48 (0.91 to 20.78)/13.83 (1.15 to 27.73)	12,373.57 (493.94 to 38,772.43)/45,041.39 (1835.45 to 139,993.68)	9.86 (0.82 to 19.9)/13.1 (1.08 to 26.09)
Middle SDI	14,869.26 (563.44 to 48,060.03)/69,334.61 (2706.71 to 219,022.4)	11.12 (0.91 to 23.18)/13.79 (1.12 to 28.33)	5344.85 (201.02 to 17,753.51)/24,375.33 (947.32 to 78,104.26)	11.62 (0.97 to 24.09)/14.23 (1.14 to 29.28)	9524.4 (364.56 to 30,332.9)/44,959.28 (1759.39 to 143,574.44)	10.9 (0.89 to 22.74)/13.63 (1.11 to 27.85)
Low-middle SDI	6394.09 (234.99 to 20,480.52)/30,601.01 (1133.95 to 98,703.47)	10.77 (0.87 to 22.27)/15.81 (1.27 to 32.33)	2745.01 (101.24 to 8906.34)/11,674.29 (433.04 to 38,369.25)	11.16 (0.92 to 22.99)/16.38 (1.31 to 33.67)	3649.08 (133.75 to 11,687.46)/18,926.72 (700.91 to 60,638.98)	10.52 (0.85 to 21.74)/15.5 (1.24 to 31.54)
Low SDI	2025.29 (73.88 to 6573.13)/7756.94 (280.13 to 25,000.78)	9.7 (0.77 to 20.27)/12.71 (0.99 to 26.31)	882.44 (31.16 to 2904.17)/3109.42 (111 to 10,140.13)	11.35 (0.9 to 23.73)/14.61 (1.12 to 30.54)	1142.85 (43.33 to 3720.88)/4647.52 (169.13 to 14,934.06)	8.83 (0.71 to 18.31)/11.79 (0.92 to 24.2)
High body mass index						
Global	31,577.42 (−1617.42 to 142,814.26)/139,439.23 (−21,837.61 to 580,290.39)	4.59 (−0.41 to 13.46)/6.8 (−1.52 to 19.23)	6761.06 (−90.38 to 32,944.6)/38,848.77 (−4830.57 to 165,651.67)	3.18 (−0.06 to 9.41)/5.91 (−1.03 to 16.96)	24,816.36 (−1869.75 to 109,376.76)/100,590.45 (−16,798.59 to 413,608.69)	5.13 (−0.63 to 15.04)/7.25 (−1.76 to 20.42)
High SDI	18,458.04 (−2099.98 to 78,329.33)/61,559.12 (−10,803.76 to 246,618.99)	6.23 (−0.94 to 17.8)/8.35 (−2.11 to 23.19)	3993.3 (−291.46 to 17,265.24)/17,838.49 (−2975.95 to 74,888.32)	4.96 (−0.39 to 14.65)/7.86 (−1.86 to 21.85)	14,464.74 (−1834.25 to 61,173.11)/43,720.63 (−7792.34 to 175,942.88)	6.66 (−1.13 to 18.91)/8.62 (−2.24 to 23.95)
High-middle SDI	9283.27 (−757.25 to 42,070.33)/40,012.29 (−6603.68 to 162,473.05)	5.17 (−0.65 to 14.86)/7.82 (−2.06 to 22.1)	1864.45 (−31.12 to 9290)/9860.8 (−1259.03 to 42,475.12)	3.64 (−0.09 to 10.57)/6.34 (−1.22 to 18.08)	7418.82 (−773.04 to 32,395.85)/30,151.5 (−5278.63 to 121,355.68)	5.74 (−0.89 to 16.53)/8.52 (−2.44 to 23.83)
Middle SDI	2461.51 (−19.25 to 13,171.97)/27,311.11 (−3231.14 to 118,499.03)	1.62 (−0.03 to 5.53)/1.62 (−0.03 to 5.53)	596.41 (−42.4 to 3595.62)/7934.14 (−696.12 to 36,157.19)	1.09 (−0.16 to 4.41)/4.38 (−0.55 to 12.61)	1865.1 (−1.02 to 9536.7)/19,376.96 (−2595.22 to 82,798.63)	1.89 (−0.01 to 6.1)/5.52 (−1 to 15.47)
Low-middle SDI	1150.44 (−0.77 to 6000.14)/8920.58 (−975.01 to 38,864.55)	1.68 (−0.01 to 5.35)/4.41 (−0.69 to 12.76)	278.69 (−17.42 to 1610.57)/2750.49 (−222.4 to 12,499.15)	0.92 (−0.14 to 3.54)/3.76 (−0.48 to 10.59)	871.76 (−6.14 to 4336.16)/6170.09 (−812.94 to 26,525.23)	2.2 (−0.01 to 6.56)/4.79 (−0.86 to 13.93)
Low SDI	176.9 (−37.51 to 1142.78)/1480.97 (−31.01 to 7375.4)	0.48 (−0.47 to 2.72)/2.07 (−0.03 to 6.41)	16.11 (−39.81 to 224.05)/422.08 (−5.37 to 2149.31)	−0.09 (−1.05 to 1.64)/1.62 (−0.06 to 5.12)	160.8 (−15.56 to 934.86)/1058.89 (−59.49 to 5137.43)	0.82 (−0.27 to 3.46)/2.31 (−0.14 to 7.04)
Smoking						
Global	32,164.82 (7445.51 to 89,320.27)/67,175.82 (15,694.65 to 184,664.76)	4.32 (3.02 to 5.64)/3.31 (2.3 to 4.34)	20,500.17 (4679.59 to 57,869.89)/46,755.42 (11,060.63 to 132,714.14)	8.85 (6.31 to 11.42)/6.92 (4.87 to 9.12)	11,664.65 (2788.55 to 31,824.2)/20,420.4 (4945.89 to 53,997.88)	2.43 (1.67 to 3.25)/1.55 (1.06 to 2.07)
High SDI	13,666.22 (3193.36 to 38,022.68)/21,770.67 (5160.07 to 60,011.5)	4.65 (3.23 to 6.2)/3.23 (2.26 to 4.4)	6849.78 (1566.96 to 19,201.52)/11,745.46 (2673.97 to 33,131.58)	8.44 (5.96 to 11.03)/5.41 (3.8 to 7.25)	6816.44 (1634.25 to 19,037.46)/10,025.21 (2437.97 to 26,534.04)	3.31 (2.29 to 4.45)/2.22 (1.55 to 3.04)
High-middle SDI	7880.68 (1767.51 to 22,010.2)/18,462.72 (4278.75 to 50,254.29)	3.97 (2.79 to 5.18)/3.61 (2.46 to 4.67)	5481.35 (1242.38 to 15,243.57)/13,472.34 (3253.86 to 37,014.04)	9.52 (6.76 to 12.29)/8.27 (5.81 to 10.91)	2399.33 (547.41 to 6457.99)/4990.39 (1088.87 to 13,400.06)	1.85 (1.27 to 2.49)/1.48 (0.94 to 2.08)
Middle SDI	7295.54 (1666.64 to 20,480.3)/19,214.62 (4535.5 to 54,275.94)	4.69 (3.29 to 6.16)/3.56 (2.49 to 4.64)	5563.2 (1280.47 to 15,912.13)/15,633.18 (3761.8 to 45,815.24)	10.12 (7.24 to 12.98)/8.4 (5.95 to 10.92)	1732.34 (398.51 to 4735.21)/3581.44 (804.32 to 9648.5)	1.92 (1.26 to 2.59)/1.08 (0.71 to 1.52)
Low-middle SDI	2733.65 (625.62 to 7836.22)/6466.32 (1472.81 to 18,526.77)	4.21 (2.97 to 5.59)/3.15 (2.18 to 4.22)	2180.79 (489.46 to 6275.19)/5063.36 (1147.74 to 14,560.03)	8.18 (5.71 to 10.68)/6.71 (4.72 to 8.89)	552.86 (130.8 to 1561.42)/1402.96 (327.9 to 3955.91)	1.52 (1.02 to 2.13)/1.13 (0.76 to 1.59)
Low SDI	559.62 (130.74 to 1625.67)/1216.04 (286.56 to 3510.84)	2.38 (1.65 to 3.19)/1.84 (1.28 to 2.42)	406.28 (92.66 to 1195.07)/812.19 (189.52 to 2344.82)	4.53 (3.19 to 5.99)/3.43 (2.42 to 4.53)	153.34 (35.85 to 438.31)/403.85 (96.08 to 1136.07)	1.16 (0.78 to 1.57)/1 (0.67 to 1.43)

PAF = population attributable fraction, SDI = sociodemographic index, UI = uncertainty interval.

### 3.7. Future forecasts of global burden of dementias

**Figure 10.**
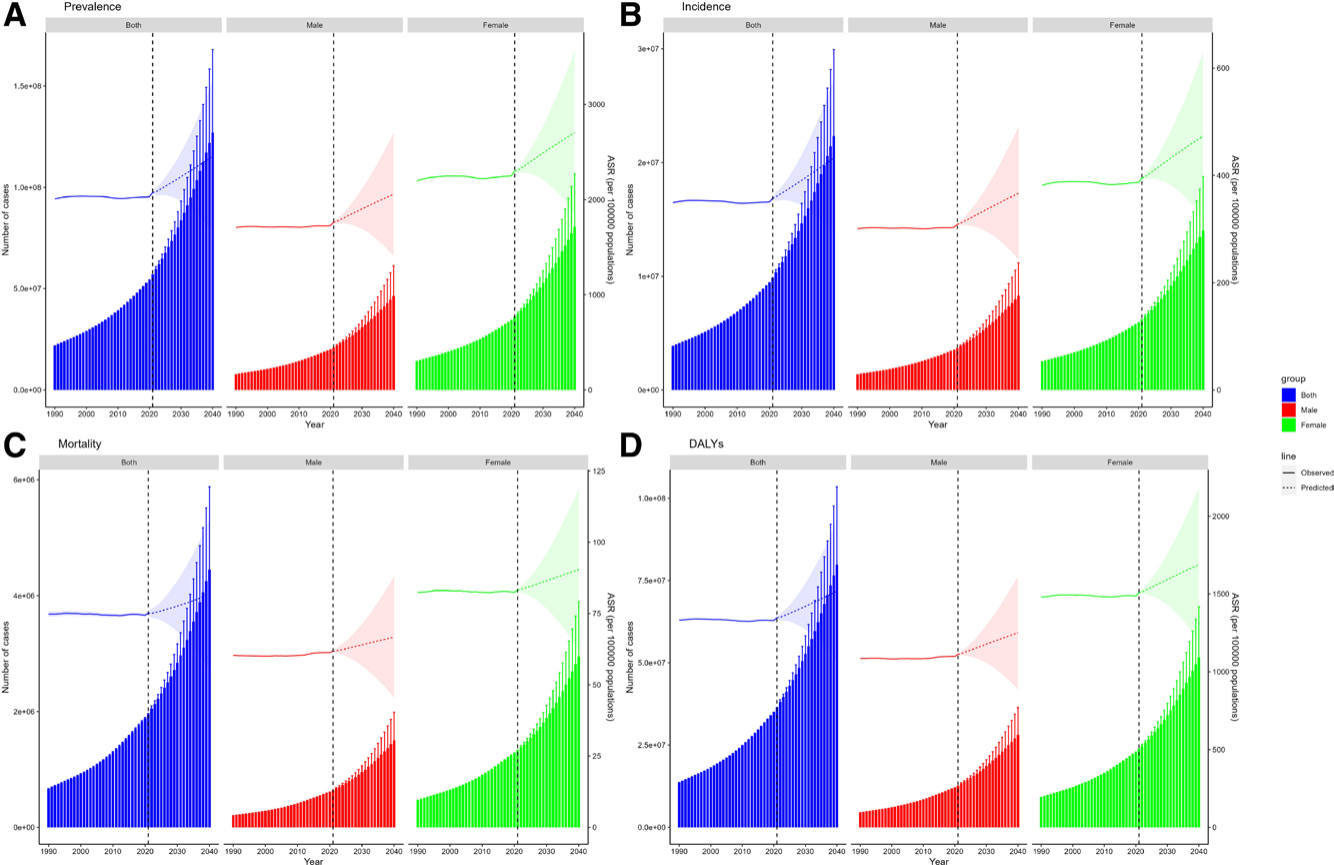
Future forecasts of global burden of dementias. (A) The forecasts of age-standardized prevalence rate. (B) The forecasts of age-standardized incidence rate. (C) The forecasts of age-standardized mortality rate. (D) The forecasts of age-standardized disability-adjusted life-year rate. ASR = age-standardized rate, DALY = disability-adjusted life-year.

The global burden of dementia is expected to undergo significant alterations from 2021 to 2040, presenting distinct tendencies for different indicators. As illustrated in the projections in Figure 10, the global age-standardized prevalence rate of dementia is anticipated to rise from 2073.21/100,000 in 2021 to 2454.96/100,000 in 2040, with an increase of 18.43%. The age-standardized prevalence rate of males increased from 1760.95/100,000 to 2055.55/100,000 (expressed as an approximation for 2040 for comparison), with a growth rate of 16.73%. The age-standardized prevalence rate of females rose more conspicuously, from 2300.02/100,000 to 2702.40/100,000, with an increase rate of 17.49%. The prevalence disparity between women and men expanded over time. The global age-standardized incidence rate for dementia is projected to increase from 357.74 per 100,000 in 2021 to 432.62 per 100,000 in 2040, an augmentation of 20.93%. The age-standardized incidence rate of males ascended from 308.88/100,000 to 366.54/100,000, with an increase of 18.67%. The growth rate of ASIR in women was higher than that in men, approximately 19.74%. The age-standardized disability-adjusted life year rates of dementia is expected to continuously increase in the next 20 years, with an overall growth rate of approximately 12.72%. The age-standardized disability-adjusted life year rates increased by 12.35% in males and 11.86% in females, with no significant difference between them. The global age-standardized mortality rate of dementia is anticipated to ascend from 75.13 per 100,000 people in 2021 to 81.81 per 100,000 people in 2040, with an increase of 8.89%. The growth rate of age-standardized mortality rate was 7.97% in males and 8.56% in females, which manifested a similar growth trend. The results of this study imply that the global burden of dementia will continuously increase in the next 2 decades, with a heavier burden in women. This trend might be associated with the longer life expectancy of women, physiological differences, as well as social roles and environmental factors. Therefore, gender differences should be fully considered in the development of prevention and control strategies for dementia, and more attention and care should be given to female patients.

## 4. Discussion

As the inevitable consequence of the transformation of population structure, population aging has emerged as the core issue of human society in the 21st century. The proportion of the global population aged 65 years and older is projected to nearly double from 8.5% in 2015 to 16.7% in 2050, as stated by the researchers. With the advancement of population aging, dementia has become a global public health challenge. A comprehensive analysis of the global dementia burden during the period from 1990 to 2021 revealed the disparities and changing trends in the burden of dementia across geographic regions and SDI regions. These findings not only highlight the extensive impact of dementia worldwide but also emphasize the specific distribution patterns and development trends of dementia in different socioeconomic backgrounds, providing scientific evidence for the formulation of more precise and effective strategies for the prevention and control of dementia.

From 1990 to 2021, the global age-standardized prevalence rate, age-standardized prevalence rate, and age-standardized disability-adjusted life year rate of dementia all exhibited a notable upward trend throughout the study period, with increments of 160.81%, 156.54%, and 167.7%, respectively. This data is consistent with the findings of the previous GBD Study 2019, which revealed that the global burden of dementia has been escalating year by year from 1990 to 2021. In 2019, Alzheimer disease was officially designated as the sixth leading cause of death in the United States. When the COVID-19 emerged as the third leading cause of death in 2020 and 2021, Alzheimer disease remained the seventh. In fact, the number of deaths attributed to dementia is far greater than the reported number of deaths from Alzheimer disease. In 2019, the officially recorded number of deaths due to dementia was 271,872, among which 121,499 were from Alzheimer disease.^[10]^ It is noteworthy that despite the escalating overall burden, there has not been a significant increase in the global age-standardized mortality rate in recent times, which might be attributed to the increasingly advanced diagnosis and treatment technologies and the continuous enhancement of care. At the regional level, high-middle SDI areas manifested the highest prevalence, incidence, mortality, and disability-adjusted life year rates of dementia in 2021, followed by high SDI regions. Meanwhile, high-middle SDI regions also took the lead in terms of the annual growth rates of age-standardized prevalence rate and age-standardized prevalence rate. This finding further emphasizes the persistent socioeconomic disparities in the global dementia burden, where regions with higher SDI values often bear a higher disease burden of dementia. This might be closely related to the extension of life expectancy, the intensification of population aging, and the improvement of medical expenditure and the level of diagnosis and treatment services in high SDI areas. Nonetheless, in recent years, the age-standardized mortality rate and age-standardized disability-adjusted life year rate of dementia in low SDI and low-middle SDI areas have also exhibited an ascending trend. This phenomenon is likely the outcome of a combination of factors, including, but not limited to, restricted access to local healthcare, suboptimal management of risk factors, and insufficient identification of dementia risk factors and symptoms. The global economic burden of dementia and Alzheimer disease is immense, and it is projected to reach a staggering US $14,513 billion between 2020 and 2050. It is notable that this economic burden is not uniformly distributed globally, presenting significant regional variances. East Asia and the Pacific emerged as the region with the highest economic burden, followed by Europe, Central Asia, and North America. At the country-specific level, the economic burden of ADOD in China is particularly prominent, estimated at approximately US $2961 billion. The United States and Japan come next, also encountering substantial economic pressures. In 2020, caregivers for individuals suffering from Alzheimer disease or other dementia in the United States provided approximately 18 billion hours of informal pro bono assistance, contributing nearly $340 billion. This amounts to approximately 59 percent of Wal-Mart’s annual net revenue of $572.8 billion and 14 times that of McDonald ($23.3 billion).^[11]^ These data not only reveal the profound impact of ADOD on the global economy but also emphasize the significant inequalities in the economic burden of the disease across diverse regions and countries. When addressing the global challenge of dementia, we also need to pay increased attention to the influence of socioeconomic differences on the burden of dementia and adopt targeted measures to promote the equity and efficacy of global dementia prevention and control.

From an individual perspective, dementia is not a disease confined to the elderly population. Despite its considerable correlation with age, it sporadically emerges among young and middle-aged individuals. In this study, the prevalence, incidence, mortality, and disability-adjusted life year rates of dementia were all positively correlated with age. The burden of dementia increases significantly with age. Notably, individuals aged 65 to 69 years presented the most substantial annual increments in prevalence, incidence, and mortality compared with the 2019 GBD study, a shift that occurred 5 years earlier. We postulate that this phenomenon might be closely related to the younger trend of chronic diseases such as cardiovascular and cerebrovascular disorders, hypertension, and hyperlipidemia, as well as risk factors like smoking, drinking, poor eating habits, and the social environment. Globally, women have higher prevalence, incidence, mortality, and disability-adjusted life year rates of dementia than men. According to an epidemiological survey in the United States, approximately two-thirds of individuals with Alzheimer disease are women. Among the 6.7 million people aged 65 years and above with Alzheimer dementia, 4.1 million are women and 2.6 million are men.

The Lancet report also suggested that the prevalence of Alzheimer disease among Chinese women was 2.37 times that of men. Although age is a primary risk factor for dementia and women tend to have a longer lifespan than men, the differences in survival do not fully explain this gender disparity. Several recent studies have hypothesized that estrogen might play a role in protecting the brain from damage caused by Alzheimer disease, such that this protective effect may be weakened or lost when estrogen levels decline.^[12]^ Peaks in cognitive decline have been suggested to be associated with the accumulation of visceral fat, energy imbalance, and menopausal bone loss.^[13,14]^ Numerous studies consistently demonstrate that the APOE-e4 genotype is the most well-established common genetic risk factor for Alzheimer disease, with more pronounced associations observed in the female population regarding Alzheimer disease and neurodegeneration. A recent meta-analysis revealed that while age plays a crucial role in the association between APOE-e4 and Alzheimer disease, there is no significant gender-based variation observed in the overall association of APOE-e4 with Alzheimer disease.^[15,16]^ The researchers conducted a comprehensive analysis revealing that the APOE gene exerts a crucial influence on individuals aged 55 to 70 years, especially among women who exhibit heightened vulnerability to Alzheimer disease during this specific age bracket. Nevertheless, it still remains an unresolved mystery within the scientific community whether estrogen, a sex hormone, regulates the impact of APOE-e4. The burden of protective and risk factors, as well as Alzheimer pathology and related diseases (such as cerebrovascular disease), varies between genders due to disparities in biological factors (e.g., genetic risk) and social constructs (e.g., educational attainment and lifestyle).^[17–19]^

In its latest 2024 report, the Lancet Commission has presented a comprehensive evaluation of the modifiable risk factors contributing to dementia on a global scale, based on ongoing research since 2020. The report unveils that approximately 45% of dementia cases worldwide can be attributed to fourteen modifiable risk factors. Compared to the previous 2020 report, 2 new risk factors have been identified: elevated low-density lipoprotein cholesterol levels during midlife (45–65 years) and vision impairment in late life (>65 years). This discovery underscores the significance of actively managing these modifiable risk factors from childhood onward in order to prevent or delay the onset of dementia in nearly half of all cases. However, it is important to note that only high fasting glucose levels, high BMI, and smoking are explicitly listed as risk factors for dementia in the GBD 2021 database. This discrepancy suggests an ongoing deepening and refining of our understanding regarding the risk factors associated with dementia. Insulin resistance not only results in diminished insulin signaling within the central nervous system but also triggers a cascade of alterations in brain metabolism. Specifically, insulin resistance is regarded as a pivotal molecular mechanism linking fasting hyperglycemia to Alzheimer disease. More precisely, it can facilitate the heightened toxicity of β-amyloid, hyperphosphorylation of tau protein, oxidative stress response, and neuroinflammation, all of which collectively contribute to the progression of dementia.^[20]^ The presence of diabetes has been identified as a significant risk factor for the development of dementia in middle-aged individuals. In a prospective cohort study involving 10,095 participants, it was observed that the likelihood of developing dementia increased significantly with each 5-year reduction in age at which type 2 diabetes manifested (hazard ratio [HR] = 1.24, 95% CI 1.06–1.46).^[21]^ The findings from another study examining fasting glucose levels in nondiabetic adults aged >65 years revealed that individuals with a blood glucose level of 115 mg/dL exhibited a significantly heightened risk of developing dementia within a span of 5 years, as compared to those with a blood glucose level of 99 mg/dL (HR = 1.18, 95% CI, 1.04–1.33).^[22]^ The relationship between BMI and dementia risk has been a topic of debate. In 2020, Qu conducted a comprehensive systematic review and meta-analysis of 14 high-quality studies involving 77,890 subjects to investigate the potential link between obesity status and dementia. The results showed that midlife obesity was significantly associated with all-cause dementia, with a relative risk of 1.31 (95% CI: 1.02–1.68) at a quantitative cutoff value of 29 kg/m^2^. This finding provides strong evidence supporting the notion that obesity increases the risk for developing dementia.^[23]^ The subsequent analysis revealed that obese individuals frequently exhibit inadequate levels of physical activity, which not only exacerbates the detrimental effects of obesity itself but also renders them more susceptible to hypertension, diabetes, hyperlipidemia, as well as cardiovascular and cerebrovascular diseases. These concurrent chronic conditions, through intricate pathophysiological mechanisms, may directly or indirectly contribute to cognitive decline and consequently heighten the risk of dementia.^[24]^ Additionally, it is noteworthy that weight stigma is also prevalent among individuals with high BMI, and this psychological burden is strongly correlated with elevated cortisol levels, heightened inflammatory responses, and a range of adverse health outcomes. As a stress hormone, prolonged elevation of cortisol may have detrimental effects on brain structure and function, while inflammation serves as the underlying pathological mechanism for numerous chronic diseases and cognitive decline.^[25–27]^ Therefore, it is theoretically plausible that the sequence of physiological changes triggered by weight shame could also serve as an indirect pathway linking a high BMI to the susceptibility of developing dementia.^[28]^ Although a previous 2020 Lancet Commission identified smoking as a risk factor in later life, accumulating evidence now suggests that smoking during midlife poses greater dangers than smoking in older age. For instance, findings from a UK Biobank study involving 497,401 adults revealed that individuals under the age of 50 years who smoke had a HR of 1.74 (95% CI: 1.23–2.47) for dementia, while those over the age of 65 had an HR of 1.74 (95% CI: 1.23–2.47).^[29]^ The act of smoking was also found to be associated with an elevated risk of dementia overall (HR = 1.28, 95% CI: 1.18–1.40), which aligns with the findings of the previous meta-analysis.^[30]^ The findings from 2 prospective cohort studies conducted in Denmark, involving a total of 61,664 participants, revealed that male smokers exhibited a significantly elevated risk of developing dementia, with a HR of 3.2 (95% UI: 1.4–7.4). Similarly, female smokers also experienced an increased risk of 1.7 (95% UI: 1.1–2.8) compared to never-smokers.^[31]^ From a biological perspective, smoking significantly increases the risk of stroke and further exacerbates the risk of developing vascular dementia.^[32]^ Additionally, it directly impacts the onset of Alzheimer disease in an adverse manner. Abundant research data strongly support that smoking plays a pivotal role in promoting oxidative stress and inflammation, which are crucial components in the core pathological process of Alzheimer disease.^[30,33]^

The treatment of dementia is dependent on the etiology and stage of the condition.^[6,34]^ Cholinesterase inhibitors, memantine, and antiamyloid (Aβ) immunomodulators are utilized to moderately slow cognitive decline in patients with Alzheimer disease or mild dementia.^[35]^ Nonpharmacological interventions, such as identifying triggers and implementing personalized behavioral therapies, are preferred for managing psychiatric and behavioral symptoms associated with dementia. The use of antipsychotic medications lacks substantial evidence regarding their efficacy in treating these symptoms while potentially increasing mortality rates, risk of significant falls, and cognitive decline.^[36–39]^ To date, there is currently no efficacious treatment available for dementia. Therefore, the management and prevention of risk factors associated with dementia, along with prompt identification and early diagnosis of the condition, are imperative in mitigating the global disease burden.

The present study entails a meticulous and comprehensive analysis of the global and regional burden of dementia, as well as its associated risk factors, based on the most recent epidemiological data spanning from 1990 to 2021 across 204 countries and regions. However, it is crucial to acknowledge certain limitations that arise from the execution and interpretation of this study. First, dementia subtypes were not further classified. Second, it should be noted that the GBD database does not encompass all populations and regions, leading to uneven data quality. Furthermore, it is important to highlight that the current version of the database (as of 2021) encompasses only a limited number of risk factors for dementia while failing to capture the complete spectrum of complex causes underlying this disease.

## Acknowledgments

The authors extend their sincere gratitude to the contributors of the Global Burden of Disease, Injury, and Risk Factor Study 2021 for their invaluable efforts.

## Author contributions

**Validation:** Songxin Zhong, Chi Gong, Changqiang Feng.

**Visualization:** Songxin Zhong, Chi Gong, Changqiang Feng.

**Writing – original draft:** Songxin Zhong, Chao Qin, Yanni Lin.

**Writing – review & editing:** Songxin Zhong, Chao Qin, Yanni Lin.

**Funding acquisition:** Chao Qin.

**Investigation:** Chao Xiao, Rida Li, Hengchang Qi.

**Methodology:** Chao Xiao, Rida Li, Hengchang Qi.

**Project administration:** Chao Xiao, Yining Lan.

**Software:** Chao Xiao, Yining Lan.

**Resources:** Yining Lan.

**Supervision:** Chi Gong, Changqiang Feng.

## Supplementary Material


