# Supplementary material for: The global, regional, and national burdens of dementia in 204 countries and territories from 1990 to 2021: A trend analysis based on the Global Burden of Disease Study 2021
Source: Medicine (Baltimore). 2025 Mar 14;104(11):e41836. doi: 10.1097/MD.0000000000041836 (PMC11922445; doi:10.1097/MD.0000000000041836)
Supplement: Supplementary file 1 [file medi-104-e41836-s001.pdf]

Supplement table 1: The prevalence cases and age-standardized prevalence rate of dementias in 1990 and 2021 and its trends.

Location	Prevalence				
	1990 counts (95%UI)	2021 counts (95%UI)	1990 ASR (95%UI)	2021ASR (95%UI)	AAPC (95%CI)
Afghanistan	38675.277 (30074.254-48739.26)	49010.338 (37820.874-62420.008)	2323.855 (1808.132-2917.982)	2275.826 (1773.6-2867.287)	-0.07 (-0.08, -0.06)
Albania	10616.683 (8181.509-13490.291)	27825.659 (21713.457-34960.321)	1930.949 (1492.126-2444.943)	1938.083 (1509.868-2438.077)	0.01 (0.01, 0.02)
Algeria	68758.087 (53665.77-86930.794)	213904.377 (166442.432-270523.118)	2351.184 (1835.562-2959.95)	2248.365 (1751.858-2833.814)	-0.14 (-0.16, -0.13)
American Samoa	92.254 (70.03-118.244)	232.47 (176.994-296.071)	1913.608 (1464.672-2429.968)	1871.932 (1430.566-2375.955)	-0.07 (-0.08, -0.07)
Andorra	338.904 (261.701-428.391)	1085.737 (837.651-1371.179)	2058.04 (1590.193-2598.681)	1927.079 (1487.167-2435.223)	-0.21 (-0.23, -0.19)
Angola	16851.962 (13037.023-21448.975)	52578.06 (40782.181-66682.4)	2273.218 (1774.796-2858.41)	2207.122 (1723.701-2771.452)	-0.09 (-0.1, -0.08)
Antigua and Barbuda	330.269 (256.352-416.404)	504.434 (389.369-641.905)	1670.239 (1293.472-2110.894)	1630.205 (1260.024-2068.771)	-0.08 (-0.09, -0.07)
Argentina	182118.623 (141261.245-229747.167)	347969.496 (269482.839-439783.975)	1865.83 (1447.115-2353.6)	1770.093 (1369.592-2239.021	-0.17 (-0.19, -0.15)

				)	
Armenia	14696.737 (11402.253-18474.101)	28418.452 (22153.812-35854.973)	1957.44 (1521.462-2456.139)	1947.255 (1515.644-2458.61)	-0.01 (-0.02, -0.01)
Australia	132333.273 (104160.471-164488.755)	300594.008 (242577.09-367652.716)	2099.562 (1651.891-2610.352)	1762.321 (1414.322-2163.249)	-0.56 (-0.58, -0.54)
Austria	89764.197 (69744.117-113320.213)	143897.79 (110850.077-181912.021)	2091.549 (1619.487-2648.082)	1954.528 (1500.847-2478.648)	-0.22 (-0.23, -0.21)
Azerbaijan	26702.547 (20765.461-33662.561)	47756.55 (36993.312-60785.607)	1942.191 (1511.892-2446.013)	1880.55 (1461.612-2381.347)	-0.1 (-0.11, -0.09)
Bahamas	724.264 (559.126-920.893)	1860.249 (1433.787-2363.693)	1665.938 (1288.601-2111.904)	1630.123 (1260.53-2065.159)	-0.07 (-0.08, -0.07)
Bahrain	745.82 (573.118-950.855)	3851.818 (2944.019-4955.203)	2357.251 (1829.927-2962.378)	2287.36 (1780.197-2884.112)	-0.1 (-0.11, -0.08)
Bangladesh	170705.176 (132229.725-216183.31)	516766.086 (399229.679-654675.861)	1379.108 (1071.923-1740.233)	1326.512 (1026.298-1677.682)	-0.13 (-0.14, -0.12)
Barbados	1842.881 (1437.043-2315.154)	2857.434 (2218.201-3612.665)	1689.836 (1311.186-2129.617)	1620.006 (1255.707-2050.334)	-0.14 (-0.15, -0.13)
Belarus	82518.788 (64049.914-104382.828)	110570.991 (85982.769-139671.344)	2002.861 (1552.304-2535.483)	1997.044 (1551.178-2521.446)	-0.01 (-0.01, 0)
Belgium	128420.695 (101663.542-158986.772)	198767.066 (153513.554-250390.68)	2352.526 (1855.414-2921.728)	2072.538 (1595.609-2617.284)	-0.41 (-0.42, -0.4)

Belize	523.727 (406.983-660.544)	1391.697 (1082.055-1761.304)	1734.799 (1348.957-2186.84)	1666.821 (1302.217-2100.578)	-0.13 (-0.14, -0.12)
Benin	7821.427 (6029.653-9961.94)	16564.253 (12908.874-20944.368)	1431.301 (1104.645-1819.45)	1280.063 (1004.197-1610.809)	-0.36 (-0.38, -0.34)
Bermuda	324.7 (251.968-411.862)	870.045 (677.011-1100.982)	1723.281 (1336.631-2185.54)	1694.624 (1315.714-2148.901)	-0.05 (-0.06, -0.05)
Bhutan	729.142 (557.802-934.102)	2324.573 (1792.227-2959.568)	1410.55 (1088.725-1791.416)	1295.588 (1000.555-1646.217)	-0.27 (-0.28, -0.27)
Bolivia (Plurinational State of)	11199.987 (8615.258-14223.328)	33654.976 (25927.357-42794.305)	1386.364 (1070.233-1753.919)	1363.217 (1054.106-1727.67)	-0.05 (-0.06, -0.05)
Bosnia and Herzegovina	20175.137 (15563.654-25609.007)	41105.317 (31901.474-51694.777)	1914.037 (1480.213-2423.845)	1915.034 (1483.277-2410.797)	0 (-0.01, 0.01)
Botswana	2235.403 (1732.766-2829.062)	6048.31 (4655.129-7661.884)	1860.711 (1447.842-2341.886)	1784.715 (1382.184-2248.019)	-0.14 (-0.14, -0.13)
Brazil	526396.212 (411365.22-662505.268)	1825794.909 (1426435.216-2299979.947)	2269.569 (1776.752-2850.255)	2274.685 (1779.164-2863.227)	0 (-0.02, 0.02)
Brunei Darussalam	435.485 (333.027-555.149)	1331.011 (1019.787-1693.871)	1736.467 (1335.26-2200.174)	1729.53 (1334.195-2186.956)	-0.01 (-0.03, 0.01)
Bulgaria	66300.58 (51231.64-83832.564)	98848.154 (76762.266-124450.042)	1965.217 (1515.888-2488.396	1922.201 (1489.054-2426.448	-0.07 (-0.08, -0.07)

			)	)	
Burkina Faso	14687.074 (11383.969-18599.528)	30516.966 (23388.797-38950.784)	1434.966 (1114.301-1810.932)	1337.783 (1029.224-1698.036)	-0.23 (-0.23, -0.22)
Burundi	10635.669 (8272.559-13459.557)	18569.384 (14337.679-23609.384)	1889.537 (1471.94-2383.405)	1735.429 (1346.723-2190.427)	-0.27 (-0.28, -0.27)
Cabo Verde	1120.8 (876.365-1411.678)	1800.192 (1386.609-2289.636)	1391.334 (1083.281-1756.319)	1317.298 (1017.894-1670.543)	-0.18 (-0.19, -0.17)
Cambodia	20918.207 (16177.752-26516.024)	59413.39 (45853.469-75433.659)	2040.906 (1583.264-2575.236)	1967.488 (1520.573-2486.809)	-0.12 (-0.13, -0.11)
Cameroon	13890.028 (10696.653-17679.499)	36246.166 (27745.195-46405.881)	1331.298 (1030.238-1684.935)	1258.952 (969.753-1599.698)	-0.18 (-0.19, -0.17)
Canada	285209.964 (232144.869-342181.25)	631350.311 (512901.459-758059.823)	2645.663 (2152.739-3176.076)	2366.87 (1918.988-2845.509)	-0.36 (-0.42, -0.3)
Central African Republic	4964.16 (3827.118-6273.679)	9274.367 (7167.846-11769.425)	2389.86 (1863.631-2987.933)	2375.46 (1869.082-2963.083)	-0.02 (-0.04, 0)
Chad	10625.75 (8194.95-13462)	17240.699 (13226.306-21970.924)	1412.76 (1091.14-1786.613)	1278.981 (987.511-1618.199)	-0.32 (-0.33, -0.31)
Chile	53092.385 (41698.514-66420.859)	159046.603 (123630.021-200403.316)	1825.288 (1434.165-2280.654)	1795.619 (1394.389-2264.625)	-0.06 (-0.07, -0.04)
China	4024535.832 (3096553.007-5119415.051	16990827.32 (13142115.77-21474308.738	2100.519 (1625.425-2663.056	2691.031 (2087.158-3395.199	0.75 (0.7, 0.8)

	)	)	)	)	
Colombia	91163.168 (70872.785-114681.762)	362960.552 (282291.956-459070.772)	1934.162 (1508.286-2425.451)	1908.55 (1486.212-2412.382)	-0.04 (-0.05, -0.03)
Comoros	795.399 (617.419-1008.934)	2185.942 (1696.893-2754.785)	1826.753 (1421.216-2303.408)	1738.926 (1353.212-2182.096)	-0.16 (-0.17, -0.15)
Congo	4909.973 (3790.055-6225.73)	11812.918 (9643.6-14326.027)	2219.332 (1723.188-2793.981)	2109.293 (1750.996-2517.31)	-0.16 (-0.18, -0.15)
Cook Islands	61.308 (46.668-77.882)	161.552 (124.245-204.697)	1924.816 (1471.203-2436.296)	1886.904 (1449.342-2392.975)	-0.07 (-0.07, -0.06)
Costa Rica	10290.444 (8003.857-12976.672)	35074.125 (27171.054-44398.543)	1932.396 (1504.245-2434.563)	1885.303 (1461.881-2385.219)	-0.08 (-0.09, -0.07)
Coted'Ivoire	10628.564 (8126.241-13662.54)	31517.828 (23997.768-40371.699)	1333.068 (1029.405-1694.898)	1280.831 (984.79-1627.08)	-0.13 (-0.14, -0.12)
Croatia	35201.87 (27294.521-44664.665)	65214.809 (50446.438-82491.859)	2004.643 (1555.517-2540.155)	1934.122 (1492.336-2453.768)	-0.11 (-0.13, -0.1)
Cuba	51187.819 (40350.584-64043.208)	111074.563 (86827.09-138784.803)	1556.878 (1223.931-1949.609)	1571.498 (1227.405-1965.123)	-0.01 (-0.07, 0.04)
Cyprus	4485.667 (3447.458-5697.437)	13693.807 (10571.475-17305.111)	2061.645 (1588.406-2607.555)	1976.278 (1522.674-2502.445)	-0.14 (-0.14, -0.13)
Czechia	86215.545 (66907.665-108884.039)	150648.729 (117023.298-189994.427)	1922.422 (1488.714-2431.351)	1906.442 (1477.676-2409.621)	-0.03 (-0.04, -0.02)

Democratic People's Republic of Korea	69717.014 (53024.666-88840.26)	177068.328 (136809.183-224554.097)	1887.727 (1445.254-2389.725)	1848.921 (1428.45-2340.298)	-0.07 (-0.08, -0.05)
Democratic Republic of the Congo	69182.897 (53630.426-87861.248)	172192.179 (133777.333-217864.961)	2231.224 (1742.486-2806.756)	2258.099 (1770.135-2830.705)	0.04 (0.03, 0.05)
Denmark	52763.307 (41818.056-65587.575)	64491.649 (50382.371-80718.32)	1753.338 (1383.265-2187.828)	1418.103 (1102.28-1782.256)	-0.69 (-0.72, -0.66)
Djibouti	486.255 (374.565-617.706)	2298.609 (1772.864-2938.357)	1906.567 (1485.601-2393.778)	1801.701 (1402.951-2278.128)	-0.18 (-0.19, -0.17)
Dominica	331.829 (258.701-419.379)	422.338 (326.499-534.233)	1700.366 (1322.136-2152.648)	1650.02 (1275.694-2085.801)	-0.1 (-0.11, -0.09)
Dominican Republic	16999.883 (13296.549-21360.332)	54453.251 (42522.614-68131.494)	1691.404 (1324.447-2120.398)	1710.665 (1337.365-2138.029)	0 (-0.03, 0.03)
Ecuador	21194.675 (16381.555-26995.819)	70162.802 (54008.534-89010.178)	1393.661 (1079.475-1770.815)	1359.366 (1046.052-1723.805)	-0.08 (-0.09, -0.07)
Egypt	126457.557 (100310.124-157262.462)	283117.839 (221004.139-355790.267)	2253.986 (1795.08-2784.6)	2171.497 (1700.395-2709.743)	-0.12 (-0.14, -0.1)

El Salvador	17374.09 (13464.996-21922.918)	43345.725 (33667.175-54713.101)	1884.076 (1463.081-2374.02)	1905.681 (1481.215-2406.798)	0.04 (0.03, 0.05)
Equatorial Guinea	932.594 (723.576-1179.577)	2470.88 (1912.007-3127.824)	2303.313 (1799-2891.345)	2201.39 (1717.169-2763.859)	-0.15 (-0.15, -0.14)
Eritrea	3497.698 (2687.516-4473.444)	9856.425 (7561.466-12542.707)	1920.18 (1492.57-2419.056)	1799.631 (1394.136-2266.926)	-0.21 (-0.22, -0.2)
Estonia	13049.646 (10147.858-16447.657)	21191.902 (16465.591-26604.569)	1963.542 (1523.686-2477.052)	1938.23 (1501.324-2441.599)	-0.04 (-0.06, -0.02)
Eswatini	1151.821 (888.092-1462.674)	2036.239 (1575.094-2591.358)	1804.555 (1396.491-2276.883)	1730.371 (1344.41-2189.865)	-0.14 (-0.14, -0.13)
Ethiopia	74977.906 (57633.194-95653.022)	194300.52 (149821.642-248795.799)	1906.342 (1477.092-2410.481)	1752.228 (1357.554-2231.637)	-0.27 (-0.28, -0.26)
Fiji	1436.639 (1094.159-1846.46)	3311.004 (2537.764-4221.735)	1944.432 (1493.486-2472.445)	1905.579 (1469.604-2416.125)	-0.07 (-0.09, -0.05)
Finland	50585.553 (39355.661-63074.722)	95435.821 (73699.385-121062.485)	2062.772 (1601.078-2576.938)	1842.891 (1415.775-2346.11)	-0.36 (-0.38, -0.35)
France	511499.719 (424416.484-605617.488)	924607.012 (747823.002-1124663.055)	1653.489 (1363.418-1972.261)	1568.999 (1258.073-1920.245)	-0.17 (-0.18, -0.16)
Gabon	3337.598 (2588.47-4198.667)	5306.199 (4109.385-6718.964)	2250.575 (1748.581-2820.377)	2193.985 (1707.446-2758.852)	-0.08 (-0.09, -0.07)
Gambia	1122.246 (864.477-1422.92)	3221.861 (2471.031-4098.474)	1414.009 (1094.211-1781.158)	1304.94 (1005.267-1653.48)	-0.26 (-0.27, -0.25)

Georgia	36652.099 (28511.315-46119.851)	43614.767 (33973.369-54798.635)	1944.883 (1512.219-2446.685)	1946.124 (1514.812-2448.337)	0 (-0.01, 0.01)
Germany	1093576.905 (891843.5-1330489.001)	1995249.411 (1597469.611-2431321.995)	2418.458 (1969.915-2946.959)	2451.111 (1949.328-3009.525)	0.05 (0.03, 0.06)
Ghana	18002.52 (13779.285-23050.951)	48622.607 (37242.966-61943.253)	1284.347 (988.064-1635.556)	1253.972 (966.432-1587.001)	-0.08 (-0.08, -0.07)
Greece	105423.58 (81418.048-133068.691)	212406.781 (164942.152-266496.652)	2102.067 (1620.039-2655.404)	2005.88 (1550.17-2529.072)	-0.15 (-0.16, -0.15)
Greenland	168.747 (128.898-215.261)	400.428 (309.209-507.407)	2405.799 (1848.296-3042.654)	2350.92 (1817.113-2967.262)	-0.09 (-0.12, -0.05)
Grenada	479.918 (373.847-603.356)	520.884 (402.965-660.339)	1704.503 (1324.499-2148.287)	1680.747 (1302.353-2125.298)	-0.04 (-0.05, -0.04)
Guam	305.578 (231.882-391.346)	1411.345 (1086.184-1791.137)	1889.731 (1445.751-2403.024)	1881.444 (1450.052-2386.648)	-0.02 (-0.03, 0)
Guatemala	15481.089 (11956.415-19676.911)	61937.487 (48236.645-77747.704)	1932.009 (1499.341-2444.091)	1911.479 (1489.535-2397.79)	-0.03 (-0.04, -0.02)
Guinea	12338.832 (9579.25-15632.881)	18992.785 (14604.25-24108.157)	1393.699 (1083.208-1761.512)	1310.32 (1012.594-1657.211)	-0.2 (-0.21, -0.19)
Guinea-Bissau	1123.792 (860.757-1431.534)	1865.979 (1424.244-2400.493)	1325.943 (1020.407-1679.08)	1287.92 (993.443-1641.842)	-0.1 (-0.1, -0.09)
Guyana	1638.529 (1267.237-2073.854)	2730.833 (2105.543-3461.778)	1656.92 (1286.143-2088.551)	1640.124 (1271.059-2068.184)	-0.03 (-0.04, -0.02)

Haiti	12454.538 (9611.458-15792.993)	26287.678 (20345.648-33447.406)	1751.53 (1360.151-2207.842)	1618.712 (1263.561-2043.919)	-0.26 (-0.26, -0.25)
Honduras	10395.292 (8059.172-13078.779)	31858.802 (24736.56-40174.616)	1952.86 (1516.258-2450.604)	1916.86 (1491.126-2411.591)	-0.06 (-0.07, -0.05)
Hungary	89412.528 (69094.176-112590.835)	139417.253 (108292.895-176044.871)	1927.826 (1487.455-2432.625)	1906.224 (1477.986-2411.15)	-0.03 (-0.05, -0.02)
Iceland	2349.977 (1857.724-2888.267)	4529.838 (3579.687-5562.985)	2273.342 (1793.222-2800.751)	2042.361 (1609.576-2513.035)	-0.34 (-0.36, -0.32)
India	1350127.814 (1037042.058-1729200.025)	4169684.391 (3206149.016-5335096.627)	1314.576 (1018.52-1672.029)	1302.851 (1004.842-1662.843)	-0.02 (-0.04, 0.01)
Indonesia	460739.935 (353674.128-589210.144)	1109363.844 (848231.792-1422876.559)	2027.55 (1565.238-2578.635)	1977.993 (1521.063-2524.259)	-0.08 (-0.09, -0.08)
Iran (Islamic Republic of)	133808.958 (103434.915-170355.045)	506627.064 (392038.046-642412.812)	2402.936 (1871.356-3034.58)	2316.702 (1798.38-2929.545)	-0.12 (-0.12, -0.11)
Iraq	55207.535 (42748.444-69468.121)	126348.591 (97550.618-159931.276)	2380.071 (1847.268-2988.742)	2269.37 (1762.124-2851.153)	-0.16 (-0.17, -0.15)
Ireland	27678.164 (21345.278-34928.357)	53858.615 (41421.985-67957.616)	2064.672 (1589.158-2607.113)	1880.476 (1443.108-2376.899)	-0.3 (-0.31, -0.29)
Israel	31746.204 (24547.639-40277.67)	87011.553 (67181.128-110251.543)	2025.796 (1561.583-2570.933)	1902.846 (1466.523-2413.276)	-0.2 (-0.21, -0.19)
Italy	633482.874 (493181.907-787567.013)	1427945.831 (1106751.766-1808324.904)	2070.08 (1606.798-2582.992)	2267.813 (1749.604-2881.521)	0.3 (0.27, 0.32)

Jamaica	12081.522 (9400.161-15251.263)	20331.57 (15734.277-25626.42)	1921.954 (1493.459-2429.912)	1821.518 (1410.447-2293.761)	-0.17 (-0.19, -0.15)
Japan	1023227.485 (796313.478-1294958.352)	3367357.702 (2630028.985-4252290.113)	1940.812 (1509.149-2457.004)	2013.76 (1569.813-2548.463)	0.15 (0.12, 0.18)
Jordan	6988.083 (5398.379-8874.496)	41147.493 (31922.432-51996.795)	2343.404 (1820.116-2954.21)	2350.184 (1835.921-2953.386)	0.01 (-0.06, 0.07)
Kazakhstan	67948.816 (52698.238-85925.148)	87907.495 (68131.933-111123.269)	1941.682 (1506.77-2453.144)	1894.08 (1469.053-2383.041)	-0.08 (-0.09, -0.06)
Kenya	37536.34 (28997.931-47818.374)	94643.197 (72989.347-120865.686)	1831.302 (1419.948-2323.847)	1807.525 (1403.28-2292.283)	-0.04 (-0.05, -0.03)
Kiribati	158.717 (121.976-203.006)	305.17 (235.216-388.273)	2088.46 (1616.165-2644.984)	2096.784 (1626.991-2640.278)	0.02 (0.01, 0.02)
Kuwait	3123.186 (2411.754-3943.166)	16140.606 (12456.937-20544.979)	2449.99 (1912.33-3060.475)	2305.054 (1799.463-2903.649)	-0.2 (-0.21, -0.18)
Kyrgyzstan	16675.144 (12962.592-20977.289)	23636.617 (18350.574-29679.725)	1928.066 (1497.832-2425.415)	1941.724 (1511.559-2425.122)	0.03 (0.02, 0.04)
Lao People's Democratic Republic	9180.395 (7083.847-11663.165)	21887.639 (16916.867-27726.335)	2014.386 (1559.847-2541.62)	1959.222 (1520.05-2467.125)	-0.09 (-0.1, -0.08)
Latvia	23308.6 (18034.033-29358.853)	31568.553 (24679.069-39711.949)	1969.506 (1521.97-2484.254)	1973.408 (1537.107-2490.851)	0.01 (-0.01, 0.02)

Lebanon	13979.202 (10857.999-17600.28)	54345.818 (42568.786-67985.572)	2468.531 (1921.803-3097.282)	2474.258 (1936.577-3099.742)	-0.01 (-0.03, 0.02)
Lesotho	4223.354 (3271.414-5336.024)	4510.148 (3499.398-5715.374)	1901.431 (1474.158-2397.586)	1868.545 (1453.921-2359.823)	-0.06 (-0.06, -0.05)
Liberia	3785.961 (2919.895-4797.552)	6138.296 (4715.379-7842.884)	1301.368 (1004.723-1647.502)	1254.485 (974.904-1585.092)	-0.12 (-0.14, -0.1)
Libya	12543.586 (9775.061-15784.749)	30320.883 (23425.242-38223.642)	2405.201 (1881.427-3014.985)	2278.244 (1771.905-2856.245)	-0.17 (-0.18, -0.17)
Lithuania	29311.759 (22873.142-36856.305)	44997.936 (35046.635-56482.983)	1943.431 (1513.272-2449.106)	1937.344 (1504.471-2439.067)	0 (-0.03, 0.04)
Luxembourg	3037.006 (2332.153-3843.148)	5610.085 (4352.097-7162.109)	1646.291 (1260.395-2088.329)	1415.582 (1095.867-1810.205)	-0.49 (-0.56, -0.42)
Madagascar	21748.601 (16935.088-27597.595)	38470.684 (29554.342-49255.089)	1811.405 (1414.491-2288.634)	1717.55 (1332.534-2172.518)	-0.17 (-0.18, -0.16)
Malawi	15373.515 (11878.338-19502.355)	30185.458 (23388.449-38242.106)	1815.92 (1407.49-2290.614)	1808.059 (1406.956-2276.039)	-0.01 (-0.02, -0.01)
Malaysia	53815.989 (41641.769-68099.558)	153806.867 (118379.527-195045.486)	2077.798 (1612.454-2620.157)	1964.324 (1513.858-2486.216)	-0.17 (-0.2, -0.13)
Maldives	367.006 (282.916-468.204)	1794.45 (1387.616-2274.578)	1933.903 (1499.256-2447.308)	1993.433 (1549.163-2512.468)	0.1 (0.09, 0.11)
Mali	12164.953 (9365.855-15457.221)	26749.238 (20581.813-33943.361)	1376.563 (1063.997-1737.863)	1313.193 (1016.806-1654.647)	-0.15 (-0.16, -0.15)

Malta	2725.952 (2101.332-3445.533)	7301.935 (5649.042-9225.825)	2077.521 (1600.885-2624.497)	1918.031 (1477.084-2431.993)	-0.26 (-0.27, -0.25)
Marshall Islands	65.737 (49.677-84.206)	115.543 (87.416-149.184)	1844.386 (1403.204-2348.836)	1774.676 (1361.787-2260.447)	-0.12 (-0.13, -0.12)
Mauritania	3556.259 (2740.009-4533.135)	7418.168 (5720.145-9425.343)	1398.003 (1079.823-1775.504)	1293.77 (1001.146-1639.55)	-0.25 (-0.26, -0.24)
Mauritius	3623.963 (2789.47-4622.937)	11135.547 (8622.834-14105.137)	1981.906 (1530.029-2518.238)	1963.187 (1521.302-2483.369)	-0.03 (-0.04, -0.02)
Mexico	200517.247 (154959.814-255948.409)	608490.051 (469008.342-776306.545)	1748.541 (1353.631-2227.902)	1595.817 (1231.978-2033.204)	-0.29 (-0.32, -0.27)
Micronesia (Federated States of)	244.558 (187.964-311.13)	321.396 (246.052-410.632)	2065.825 (1592.046-2613.38)	2077.317 (1603.599-2626.899)	0.02 (0.01, 0.02)
Monaco	590.213 (457.208-745.988)	769.034 (590.381-972.875)	2079.828 (1603.479-2637.595)	1909.649 (1459.608-2425.62)	-0.27 (-0.29, -0.26)
Mongolia	5381.468 (4184.197-6785.522)	10277.344 (7956.701-12998.542)	1951.889 (1522.311-2456.577)	1971.693 (1534.619-2479.098)	0.04 (0.02, 0.06)
Montenegro	3747.05 (2909.688-4739.513)	5755.242 (4461.521-7253.103)	1962.398 (1523.771-2481.261)	1909.867 (1479.104-2406.519)	-0.09 (-0.11, -0.07)
Morocco	93819.016 (73074.569-117945.798)	210666.527 (163224.712-265250.919)	2403.275 (1874.55-3016.124)	2236.083 (1734.13-2808.111)	-0.23 (-0.25, -0.21)

Mozambique	24877.24 (19271.814-31573.307)	43031.879 (33267.291-54585.531)	1866.263 (1448.699-2350.442)	1807.604 (1405.506-2273.516)	-0.1 (-0.11, -0.1)
Myanmar	115857.514 (90238.056-146668.585)	257641.176 (199774.417-326606.374)	2152.471 (1681.778-2714.857)	1982.183 (1540.454-2504.157)	-0.26 (-0.27, -0.26)
Namibia	2596.468 (2005.908-3289.2)	5765.293 (4458.534-7302.232)	1841.16 (1425.574-2314.288)	1773.501 (1375.509-2236.78)	-0.12 (-0.13, -0.12)
Nauru	16.354 (12.373-20.936)	23.239 (17.736-29.765)	1890.175 (1448.991-2396.699)	1949.629 (1501.358-2480.113)	0.1 (0.08, 0.12)
Nepal	31930.454 (24622.467-40422.587)	83342.334 (64231.719-105609.679)	1525.95 (1184.166-1917.124)	1356.806 (1047.096-1714.36)	-0.38 (-0.39, -0.37)
Netherlands	148609.507 (120418.515-177952.678)	278818.697 (220783.854-343097.942)	2124.532 (1719.772-2548.335)	2091.971 (1650.619-2582.084)	-0.05 (-0.06, -0.03)
New Zealand	27937.517 (21590.311-35332.384)	62912.812 (48418.269-79821.374)	2175.085 (1677.392-2753.539)	2045.08 (1570.482-2599.418)	-0.2 (-0.21, -0.18)
Nicaragua	8193.092 (6355.089-10334.654)	27802.045 (21620.52-35006.577)	1972.346 (1533.97-2481.34)	1956.24 (1523.502-2458.569)	-0.03 (-0.03, -0.02)
Niger	8163.596 (6269.715-10425.527)	24494.629 (18832.422-31174.654)	1405.581 (1087.65-1782.436)	1312.999 (1015.513-1661.237)	-0.22 (-0.23, -0.21)
Nigeria	139803.885 (107244.785-179269.114)	253561.462 (192590.953-326868.671)	1246.757 (959.646-1594.528)	1128.064 (864.877-1444.471)	-0.32 (-0.34, -0.31)
Niue	15.78 (12.139-20.046)	12.482 (9.523-15.922)	1898.292 (1456.788-2416.869)	1841.59 (1404.766-2346.812)	-0.1 (-0.11, -0.09)

North Macedonia	10033.763 (7722.648-12739.917)	17226.554 (13267.943-21908.926)	1915.186 (1477.287-2428.823)	1901.608 (1466.707-2413.02)	-0.02 (-0.03, -0.01)
Northern Mariana Islands	55.401 (41.472-71.726)	217.409 (164.406-279.704)	1884.378 (1435.578-2396.852)	1855.502 (1413.743-2365.689)	-0.05 (-0.08, -0.03)
Norway	59569.575 (46654.598-74800.792)	73853.448 (56787.981-93889.695)	2297.752 (1793.144-2893.83)	1909.437 (1463.882-2431.995)	-0.59 (-0.62, -0.56)
Oman	3633.494 (2804.925-4614.691)	9013.8 (6848.322-11526.354)	2352.028 (1829.017-2961.524)	2175.509 (1671.404-2750.948)	-0.26 (-0.28, -0.23)
Pakistan	205549.278 (158559.161-262804.286)	375360.279 (286749.384-482394.715)	1388.535 (1074.276-1770.969)	1295.241 (998.158-1654.095)	-0.23 (-0.24, -0.22)
Palau	43.395 (32.775-55.532)	90.737 (68.37-116.38)	1840.437 (1399.346-2345.643)	1775.315 (1347.773-2259.104)	-0.12 (-0.13, -0.1)
Palestine	5556.875 (4319.397-6995.323)	13745.511 (10671.774-17397.564)	2400.554 (1869.359-3013.438)	2311.683 (1805.127-2905.244)	-0.13 (-0.15, -0.12)
Panama	8350.819 (6448.883-10549.232)	28209.161 (21895.699-35623.228)	1877.992 (1452.554-2368.202)	1853.501 (1440.03-2338.992)	-0.04 (-0.05, -0.03)
Papua New Guinea	6961.892 (5344.359-8899.17)	19750.305 (15187.145-25190.134)	2072.159 (1608.37-2618.324)	1940.719 (1505.463-2449.6)	-0.21 (-0.22, -0.2)
Paraguay	14311.308 (11146.768-18082.292)	36298.694 (28335.254-45695.257)	2180.419 (1701.046-2749.382	2052.654 (1605.26-2579.684)	-0.19 (-0.21, -0.18)

			)		
Peru	46444.759 (36061.084-58732.538)	144346.947 (111669.343-182138.303)	1319.461 (1027.99-1663.652)	1303.848 (1010.733-1643.077)	-0.04 (-0.06, -0.03)
Philippines	148616.463 (115056.219-188683.866)	419362.683 (323754.058-534686.686)	2067.211 (1606.657-2617.328)	1980.414 (1534.86-2514.155)	-0.14 (-0.15, -0.13)
Poland	274981.903 (212180.384-348433.934)	501491.616 (386872.888-636965.542)	2041.404 (1574.014-2590.684)	1928.473 (1485.176-2452.512)	-0.18 (-0.19, -0.18)
Portugal	87614.657 (67671.975-111119.847)	203138.148 (157742.496-255812.091)	2009.265 (1548.855-2548.826)	1969.714 (1524.17-2488.928)	-0.07 (-0.07, -0.06)
Puerto Rico	20227.35 (15783.392-25408.908)	50546.787 (39344.473-64014.003)	1721.203 (1341.253-2163.855)	1683.707 (1307.981-2136.486)	-0.07 (-0.08, -0.07)
Qatar	407.756 (309.725-526.26)	3426.402 (2563.836-4440.013)	2274.102 (1772.695-2865.149)	2213.331 (1707.221-2795.288)	-0.09 (-0.1, -0.07)
Republic of Korea	149621.539 (117484.469-184797.944)	697045.329 (551626.552-860949.68)	2243.518 (1771.255-2756.292)	2210.058 (1747.634-2731.179)	-0.04 (-0.06, -0.02)
Republic of Moldova	22000.7 (17085.14-27810.281)	39085.711 (30359.193-49191.117)	1893.773 (1471.543-2392.589)	1913.451 (1484.7-2407.568)	0.03 (0.02, 0.05)
Romania	150690.364 (116894.978-190925.83)	265106.699 (205650.58-334641.518)	1907.703 (1478.522-2416.763)	1903.523 (1474.317-2405.631)	-0.01 (-0.01, 0)
Russian	1044397.55 (808037.132-1323493.112)	1605863.012 (1248722.197-2034122.726)	2001.431 (1548.383-2537.88)	1975.862 (1534.373-2504.315)	-0.04 (-0.05, -0.03)

Federation					
Rwanda	11086.145 (8615.397-14035.702)	26431.801 (20482.745-33417.964)	1851.635 (1442.797-2333.298)	1840.511 (1433.756-2312.653)	-0.02 (-0.03, -0.01)
Saint Kitts and Nevis	198.224 (153.604-250.39)	276.668 (212.747-351.748)	1613.895 (1245.517-2041.622)	1595.316 (1233.859-2018.883)	-0.04 (-0.05, -0.03)
Saint Lucia	443.084 (342.466-558.744)	1281.162 (991.799-1620.046)	1698.248 (1311.612-2143.308)	1647.668 (1276.042-2082.859)	-0.1 (-0.11, -0.09)
Saint Vincent and the Grenadines	384.832 (299.211-484.029)	734.544 (569.874-926.525)	1743.057 (1353.18-2195.713)	1654.812 (1283.35-2087.029)	-0.17 (-0.18, -0.15)
Samoa	404.382 (309.517-514.335)	712.625 (544.439-909.205)	1923.536 (1479.839-2436.922)	1860.335 (1425.096-2365.97)	-0.11 (-0.12, -0.1)
San Marino	267.006 (208.132-334.68)	604.547 (466.512-763.61)	2106.044 (1637.815-2645.352)	1861.15 (1432.284-2358.128)	-0.4 (-0.42, -0.37)
Sao Tome and Principe	240.08 (185.451-304.133)	338.945 (258.954-434.419)	1321.339 (1020.118-1671.416)	1227.622 (945.047-1564.697)	-0.24 (-0.25, -0.22)
Saudi Arabia	30755.158 (23658.164-39108.537)	71831.514 (54482.709-92818.52)	2232.769 (1727.358-2823.066)	2096.316 (1618.812-2666.557)	-0.21 (-0.24, -0.18)
Senegal	11108.031 (8588.062-14088.734)	25540.853 (19639.277-32600.736)	1380.232 (1071.941-1745.585)	1283.93 (990.936-1630.958)	-0.24 (-0.24, -0.23)

Serbia	55542.904 (42795.05-70671.868)	111693.898 (86295.266-141234.033)	1901.208 (1469.576-2406.546)	1907.423 (1470.245-2415.757)	0.01 (0, 0.02)
Seychelles	371.664 (286.546-469.043)	635.887 (487.748-807.62)	1995.327 (1537.723-2519.163)	1922.326 (1478.455-2435.948)	-0.12 (-0.12, -0.12)
Sierra Leone	7680.936 (5960.93-9733.809)	12378.409 (9561.545-15805.638)	1402.462 (1087.794-1774.109)	1321.988 (1025.343-1680.272)	-0.19 (-0.21, -0.18)
Singapore	8786.259 (6929.259-10807.579)	43790.857 (35528.478-53020.646)	1541.692 (1226.015-1883.345)	1596.7 (1297.287-1928.223)	0.11 (0.1, 0.13)
Slovakia	36501.581 (28284.49-46201.793)	60989.611 (47434.518-77044.216)	1931.3 (1495.2-2446.739)	1897.015 (1473.452-2397.929)	-0.05 (-0.07, -0.04)
Slovenia	15350.078 (11829.716-19385.414)	33191.069 (25768.622-41761.748)	1894.609 (1458.783-2395.535)	1899.287 (1471.487-2395.142)	0.01 (0, 0.02)
Solomon Islands	511.367 (391.192-654.13)	1440.008 (1103.372-1836.153)	1955.523 (1512.593-2479.438)	1949.43 (1504.071-2468.816)	-0.01 (-0.02, 0)
Somalia	8326.108 (6437.309-10583.827)	20264.145 (15680.472-25785.912)	1861.63 (1455.263-2341.322)	1843.264 (1438.955-2320.955)	-0.03 (-0.04, -0.02)
South Africa	109918.043 (84843.512-140037.992)	224952.937 (173619.478-287257.238)	1930.775 (1493.758-2453.455)	1820.802 (1407.894-2317.719)	-0.19 (-0.21, -0.17)
South Sudan	11488.563 (8928.364-14532.502)	14635.9 (11246.329-18671.251)	1840.668 (1431.316-2322.083)	1709.884 (1324.811-2160.052)	-0.23 (-0.25, -0.22)
Spain	373385.859 (307868.801-443475.568)	713870.301 (562389.881-889047.502)	2020.524 (1662.65-2406.226)	1739.062 (1365.823-2173.004)	-0.54 (-0.65, -0.43)

Sri Lanka	53753.074 (41728.085-68166.45)	152888.79 (118015.862-193810.311)	1968.28 (1532.677-2482.682)	1894.349 (1463.656-2398.073)	-0.13 (-0.15, -0.11)
Sudan	53974.221 (41961.543-67779.697)	105439.699 (82034.016-133671.691)	2362.84 (1843.318-2955.789)	2187.706 (1710.188-2760.224)	-0.25 (-0.27, -0.23)
Suriname	1330.358 (1031.109-1674.63)	3317.267 (2575.968-4192.442)	1801.223 (1400.235-2260.86)	1715.258 (1334.325-2164.989)	-0.16 (-0.18, -0.14)
Sweden	130519.303 (103472.656-161032.796)	185342.607 (143489.389-233795.8)	2256.512 (1781.805-2793.696)	2082.753 (1604.828-2640.303)	-0.25 (-0.27, -0.23)
Switzerland	83489.616 (64925.881-104657.885)	141610.165 (109418.602-178533.118)	2137.819 (1657.944-2687.031)	1911.531 (1472.481-2416.889)	-0.35 (-0.41, -0.29)
Syrian Arab Republic	32568.785 (25355.273-41221.377)	74180.565 (57500.878-94146.757)	2449.258 (1912.754-3084.782)	2251.906 (1750.526-2843.705)	-0.27 (-0.3, -0.25)
Taiwan (Province of China)	57300.007 (44347.243-72007.188)	246277.501 (193310.289-302700.7)	1516.499 (1179.351-1893.401)	1658.889 (1301.2-2037.785)	0.3 (0.26, 0.35)
Tajikistan	14980.18 (11629.218-18888.837)	23728.988 (18275.085-30325.391)	1906.328 (1481.823-2401.366)	1784.471 (1383.569-2262.513)	-0.21 (-0.22, -0.2)
Thailand	162939.975 (127656.663-204054.329)	659436.747 (512618.685-832033.015)	1796.396 (1412.508-2240.414)	1802.021 (1401.435-2273.203)	0.02 (0, 0.03)
Timor-Leste	1234.938 (956.721-1574.105)	4508.319 (3505.703-5692.55)	2125.823 (1663.643-2680.07)	1984.937 (1544.565-2502.292)	-0.22 (-0.23, -0.21)

Togo	3796.847 (2925.693-4842.418)	10754.401 (8208.99-13787.578)	1356.943 (1052.553-1717.863)	1309.085 (1008.155-1660.814)	-0.12 (-0.14, -0.11)
Tokelau	7.549 (5.775-9.587)	9.336 (7.181-11.878)	1912.892 (1464.084-2427.717)	1872.442 (1439.704-2383.441)	-0.07 (-0.08, -0.06)
Tonga	281.246 (216.643-358.058)	481.567 (371.799-608.804)	1989.089 (1536.826-2525.6)	1924.988 (1487.591-2431.16)	-0.11 (-0.11, -0.1)
Trinidad and Tobago	4189.764 (3259.636-5297.026)	10398.555 (8064.879-13125.677)	1726.804 (1342.699-2182.849)	1686.733 (1307.847-2127.843)	-0.08 (-0.08, -0.07)
Tunisia	31476.785 (24583.409-39580.785)	92441.288 (72067.6-116035.933)	2529.856 (1976.133-3167.488)	2363.241 (1844.612-2961.545)	-0.22 (-0.24, -0.19)
Turkey	235492.603 (183659.286-296110.698)	688425.25 (535224.136-865789.028)	2608.811 (2040.881-3269.478)	2447.686 (1904.126-3073.351)	-0.2 (-0.22, -0.19)
Turkmenistan	9340.665 (7224.559-11781.524)	18834.144 (14575.156-23819.44)	1916.382 (1485.085-2413.046)	1821.839 (1417.825-2292.672)	-0.16 (-0.17, -0.15)
Tuvalu	28.415 (21.818-36.317)	52.184 (40.046-66.259)	1997.406 (1544.263-2535.651)	1943.851 (1494.744-2459.054)	-0.09 (-0.1, -0.08)
Uganda	27591.799 (21405.881-34888.437)	61427.57 (47442.402-77940.429)	1797.893 (1397.768-2262.248)	1774.593 (1377.992-2236.013)	-0.04 (-0.05, -0.03)
Ukraine	438830.564 (337372.041-560705.987)	527238.112 (406322.047-670609.765)	2008.068 (1542.694-2568.027)	1945.281 (1495.945-2476.694)	-0.1 (-0.11, -0.09)
United Arab Emirates	1695.587 (1274.183-2186.895)	11960.239 (8750.71-15756.908)	2162.464 (1660.639-2735.788)	1949.018 (1492.59-2467.715)	-0.34 (-0.37, -0.31)

United Kingdom	607852.039 (472790.167-764690.088)	906466.758 (701075.275-1142443.922)	1868.877 (1448.782-2357.253)	1765.754 (1358.424-2232.848)	-0.19 (-0.2, -0.17)
United Republic of Tanzania	46620.515 (36220.044-58723.845)	108488.833 (85972.437-134936.319)	1852.477 (1442.241-2325.651)	1702.489 (1359.489-2103.307)	-0.27 (-0.28, -0.27)
United States of America	2736354.454 (2137192.928-3449021.613)	4876579.435 (3795610.604-6136272.31)	2417.538 (1885.366-3051.098)	2309.42 (1794.737-2909.591)	-0.14 (-0.16, -0.13)
United States Virgin Islands	360.912 (279.32-458.761)	1043.737 (805.671-1323.248)	1675.376 (1302.733-2118.138)	1624.568 (1250.243-2064.336)	-0.1 (-0.11, -0.09)
Uruguay	24539.052 (19080.404-30887.059)	39145.559 (30507.353-49456.08)	1878.304 (1458.347-2365.657)	1778.175 (1382.644-2252.575)	-0.18 (-0.19, -0.16)
Uzbekistan	63642.734 (49306.648-80460.045)	114617.136 (88018.021-145740.513)	1817.85 (1408.766-2297.761)	1809.093 (1397.288-2287.808)	-0.01 (-0.03, 0)
Vanuatu	228.627 (172.112-294.639)	672.378 (511.585-859.652)	1844.273 (1403.391-2348.201)	1805.145 (1382.066-2289.653)	-0.07 (-0.09, -0.06)
Venezuela (Bolivarian Republic of)	58360.437 (45616.018-73126.881)	197422.157 (154484.797-247838.641)	2189.205 (1714.713-2736.354)	2144.464 (1681.492-2687.616)	-0.07 (-0.08, -0.06)

Viet Nam	236104.736 (184068.939-298867.431)	531198.123 (410652.362-674705.782)	2059.44 (1605.203-2604.496)	1940.026 (1504.049-2457.959)	-0.2 (-0.21, -0.19)
Yemen	26830.806 (20893.739-33691.99)	75922.147 (58888.674-95936.949)	2559.222 (2000.108-3190.956)	2323.951 (1808.999-2918.45)	-0.31 (-0.32, -0.3)
Zambia	11349.99 (8801.894-14441.85)	26840.753 (20797.526-33960.199)	1760.25 (1371.43-2225.104)	1767.077 (1381.02-2216.514)	0.01 (0, 0.02)
Zimbabwe	17117.351 (13270.212-21776.281)	25485.883 (19748.032-32395.289)	1826.766 (1420.316-2314.575)	1745.882 (1361.419-2203.565)	-0.14 (-0.15, -0.14)

ASPR: age-standardized prevalence rate; AAPC: average annual percentage change; SDI: sociodemographic index; CI: confidence interval; UI: uncertainty interval.

Supplement table 2: The incidence cases and age-standardized incidence rate of dementias in 1990 and 2021 and its trends.

	Incidence

	1990 counts （95%UI）	2021 counts （95%UI）	1990 ASR （95%UI）	2021ASR （95%UI）	AAPC （95%CI）
Afghanistan	6792.347 (4572.494-9394.266)	8764.017 (5853.592-12101.404)	396.798 (268.581-545.567)	391.165 (264.297-536.048)	-0.05 (-0.05, -0.04)
Albania	1848.76 (1239.979-2591.811)	4828.202 (3272.13-6652.906)	334.422 (225.564-466.734)	336.444 (227.976-464.268)	0.02 (0.01, 0.03)
Algeria	12123.059 (8202.149-16700.263)	37174.11 (25101.132-51170.29)	400.547 (272.22-548.331)	384.126 (260.235-528.278)	-0.13 (-0.16, -0.11)
American Samoa	16.281 (10.805-22.923)	40.746 (27.286-56.808)	332.309 (223.308-463.197)	326.049 (219.687-452.643)	-0.06 (-0.07, -0.06)
Andorra	61.19 (41.032-85.024)	190.216 (128.967-259.782)	363.12 (244.335-503.725)	341.378 (231.197-465.489)	-0.2 (-0.22, -0.18)
Angola	2937.735 (1975.008-4101.339)	9171.089 (6130.459-12786.563)	380.839 (260.107-525.201)	371.044 (251.861-511.014)	-0.08 (-0.09, -0.08)
Antigua and Barbuda	57.423 (39.008-79.338)	88.127 (59.203-122.994)	290.964 (196.774-403.516)	284.032 (191.405-395.451)	-0.08 (-0.09, -0.07)
Argentina	32950.438 (22393.382-45532.32)	62824.568 (42399.358-86210.444)	335.138 (227.963-462.676)	318.833 (214.879-438.093)	-0.16 (-0.17, -0.15)
Armenia	2554.874 (1734.346-3518.449)	4929.914 (3365.001-6756.009)	341.765 (232.841-469.453)	340.562 (231.675-467.292)	-0.01 (-0.02, 0)
Australia	23451.335 (16442.198-31515.272)	52191.145 (38295.546-67948.756)	367.238 (257.041-494.344)	306.685 (223.562-401.256)	-0.58 (-0.6, -0.55)

Austria	15981.207 (10855.27-21970.966)	25290.605 (17123.379-34660.678)	369.443 (250.361-510.352)	346.792 (233.726-476.948)	-0.2 (-0.22, -0.19)
Azerbaijan	4661.612 (3147.359-6407.483)	8310.561 (5562.08-11567.439)	339.772 (229.954-466.956)	329.421 (222.038-455.17)	-0.1 (-0.11, -0.09)
Bahamas	127.528 (85.726-177.577)	325.917 (218.252-454.019)	290.623 (196.2-403.333)	284.637 (191.8-394.514)	-0.07 (-0.08, -0.06)
Bahrain	134.123 (88.777-186.443)	691.661 (457.893-970.576)	399.266 (270.51-547.741)	390.179 (263.559-536.514)	-0.08 (-0.09, -0.06)
Bangladesh	30546.439 (20568.206-42498.275)	92253.511 (61687.838-127715.896)	246.572 (166.962-341.624)	237.408 (159.365-327.461)	-0.12 (-0.14, -0.11)
Barbados	319.949 (216.825-440.567)	497.271 (336.589-686.907)	294.313 (198.378-407.921)	282.457 (190.771-390.516)	-0.13 (-0.14, -0.13)
Belarus	14328.953 (9724.801-19836.84)	19115.132 (12968.946-26305.642)	348.282 (236.485-482.178)	347.562 (235.429-478.771)	0 (-0.01, 0)
Belgium	22507.984 (15714.617-30518.644)	34244.744 (23148.473-46857.011)	408.926 (284.404-556.863)	363.227 (244.087-499.58)	-0.38 (-0.39, -0.37)
Belize	91.365 (61.808-126.45)	243.771 (163.989-338.117)	301.862 (204.609-417.273)	290.29 (196.609-400.675)	-0.13 (-0.14, -0.11)
Benin	1377.33 (911.878-1918.382)	2908.588 (1962.71-4024.18)	253.092 (168.481-351.413)	226.595 (154.859-310.981)	-0.36 (-0.37, -0.34)
Bermuda	56.316 (37.676-78.387)	150.042 (101.362-207.272)	298.132 (199.8-414.798)	293.475 (197.806-406.529)	-0.05 (-0.05, -0.05)

Bhutan	129.011 (85.241-181.805)	415.117 (277.406-577.866)	250.587 (168.048-349.545)	231.381 (155.158-321.317)	-0.26 (-0.27, -0.24)
Bolivia (Plurinational State of)	2016.257 (1351.441-2809.323)	6052.015 (4043.588-8440.243)	248.915 (168.188-345.176)	245.098 (164.793-340.441)	-0.05 (-0.06, -0.04)
Bosnia and Herzegovina	3521.348 (2366.218-4893.96)	7139.758 (4843.191-9839.952)	333.699 (225.899-461.912)	333.945 (226.098-461.299)	0.01 (-0.01, 0.02)
Botswana	391.413 (261.405-545.481)	1062.418 (709.492-1472.673)	323.204 (218.46-446.348)	310.487 (209.459-426.708)	-0.13 (-0.14, -0.12)
Brazil	90514.896 (61745.061-123785.531)	305420.634 (208064.266-421159.352)	386.396 (264.918-526.617)	379.626 (258.942-522.815)	-0.06 (-0.07, -0.04)
Brunei Darussalam	78.116 (52.23-109.371)	237.252 (158.369-331.032)	304.439 (205.852-423.805)	303.381 (205.273-418.653)	-0.01 (-0.03, 0.01)
Bulgaria	11720.64 (7839.291-16277.043)	17298.956 (11743.678-23671.924)	343.445 (230.885-476.005)	335.855 (226.961-461.84)	-0.07 (-0.08, -0.07)
Burkina Faso	2570.134 (1703.033-3575.388)	5394.015 (3560.315-7567.994)	254.845 (170.579-351.561)	238.375 (158.999-331.778)	-0.22 (-0.22, -0.21)
Burundi	1849.953 (1244.956-2565.521)	3237.26 (2152.986-4527.117)	325.88 (221.582-449.863)	300.238 (202.616-415.941)	-0.26 (-0.27, -0.26)
Cabo Verde	197.996 (133.19-275.105)	319.354 (213.869-445.118)	246.062 (165.092-342.524)	233.413 (156.971-324.231)	-0.17 (-0.18, -0.16)
Cambodia	3587.36 (2402.55-5006.307)	10161.606 (6804.473-14160.092)	346.753 (234.204-480.304)	335.204 (225.948-464.597)	-0.11 (-0.12, -0.1)

Cameroon	2454.72 (1641.686-3434.989)	6432.667 (4241.584-9036.142)	238.104 (160.925-330.531)	225.391 (150.751-313.274)	-0.18 (-0.19, -0.17)
Canada	48602.999 (35315.166-63289.953)	105263.865 (76360.033-137508.879)	446.877 (324.463-582.48)	395.508 (286.23-517.489)	-0.4 (-0.47, -0.33)
Central African Republic	864.746 (581.984-1202.202)	1621.775 (1078.265-2250.405)	396.855 (271.366-544.111)	390.947 (266.078-532.153)	-0.05 (-0.07, -0.03)
Chad	1872.128 (1253.681-2616.765)	3051.896 (2010.818-4277.227)	251.146 (169.124-349.95)	228.395 (152.563-317.765)	-0.3 (-0.32, -0.29)
Chile	9600.642 (6619.209-13028.368)	28466.358 (19325.199-39089.773)	328.163 (226.671-445.321)	322.246 (218.412-442.855)	-0.06 (-0.07, -0.04)
China	703177.998 (470346.562-974847.966)	2914112.017 (1988623.823-4006327.259)	361.809 (244.193-497.718)	452.499 (309.413-620.799)	0.68 (0.63, 0.72)
Colombia	16113.305 (10837.606-22343.614)	63668.015 (43323.334-88073.866)	339.689 (230.036-469.2)	334.965 (228.462-462.445)	-0.04 (-0.05, -0.03)
Comoros	137.927 (92.196-192.441)	380.714 (254.412-525.798)	314.444 (212.41-434.399)	301.6 (203.013-414.375)	-0.13 (-0.14, -0.12)
Congo	856.865 (576.734-1191.352)	2079.109 (1493.54-2736.708)	373.339 (254.064-513.606)	360.878 (264.667-468.517)	-0.11 (-0.12, -0.1)
Cook Islands	10.708 (7.155-14.979)	27.989 (18.804-38.788)	333.214 (224.106-463.893)	327.202 (219.681-453.906)	-0.06 (-0.07, -0.05)
Costa Rica	1812.208 (1217.522-2498.979)	6142.911 (4117.245-8539.717)	339.126 (228.205-467.148)	330.455 (221.827-458.423)	-0.08 (-0.09, -0.08)

Coted'Ivoire	1875.81 (1225.936-2642.376)	5597.972 (3660.816-7871.979)	237.634 (158.845-330.298)	228.926 (152.39-318.115)	-0.12 (-0.13, -0.11)
Croatia	6091.181 (4095.92-8400.272)	11298.715 (7668.361-15570.458)	347.924 (234.805-479.919)	336.586 (227.29-465.956)	-0.1 (-0.12, -0.08)
Cuba	9051.32 (6219.276-12372.811)	19277.529 (13279.111-26265.162)	275.504 (188.896-377.81)	273.079 (187.96-372.308)	-0.06 (-0.1, -0.01)
Cyprus	830.547 (557.312-1152.057)	2459.375 (1643.077-3389.851)	364.642 (246.147-503.075)	350.611 (233.799-485.072)	-0.13 (-0.14, -0.12)
Czechia	15054.369 (10167.83-20852.837)	26326.702 (17787.25-36227.887)	334.232 (225.9-463.159)	332.278 (223.827-458.413)	-0.02 (-0.03, -0.01)
Democratic People's Republic of Korea	12409.993 (8259.902-17281.832)	31408.03 (21035.223-43552.602)	331.27 (222.616-457.913)	324.129 (218.033-448.903)	-0.07 (-0.09, -0.05)
Democratic Republic of the Congo	12092.416 (8141.171-16892.583)	29847.15 (20024.31-41347.433)	377.541 (258.023-520.616)	379.357 (258.323-519.548)	0.01 (0.01, 0.02)
Denmark	9291.616 (6514.802-12514.147)	11465.867 (7857.522-15673.859)	307.328 (214.088-416.047)	251.948 (171.444-346.287)	-0.64 (-0.68, -0.61)
Djibouti	85.345 (56.642-119.021)	402.279 (267.912-563.85)	327.353 (221.969-449.836)	310.728 (211.142-429.454)	-0.17 (-0.17, -0.16)
Dominica	58.203 (39.469-80.414)	73.747 (49.336-101.951)	297.238 (200.914-411.976)	288.485 (193.22-398.524)	-0.1 (-0.1, -0.09)
Dominican Republic	2988.56 (2034.917-4095.631)	9479.284 (6519.514-12944.36)	296.149 (202.43-405.481)	297.314 (204.696-405.561)	-0.02 (-0.04,

					0.01)
Ecuador	3780.727 (2542.24-5258.099)	12516.955 (8356.909-17407.765)	248.387 (167.588-344.72)	242.437 (162.067-337.148)	-0.08 (-0.09, -0.07)
Egypt	22678.391 (15698.486-30894.983)	50931.448 (34620.173-69670.165)	390.547 (272.695-529.888)	377.782 (258.992-512.703)	-0.11 (-0.13, -0.08)
El Salvador	3066.419 (2064.861-4269.642)	7619.082 (5120.868-10546.06)	331.671 (223.945-460.843)	335.234 (225.41-463.733)	0.04 (0.03, 0.05)
Equatorial Guinea	161.935 (108.853-224.345)	431.306 (288.87-597.806)	386.557 (263.306-529.605)	371.758 (252.372-510.294)	-0.13 (-0.13, -0.12)
Eritrea	617.306 (406.702-867.035)	1734.657 (1143.532-2438.698)	330.526 (222.854-455.842)	311.315 (209.606-432.52)	-0.19 (-0.2, -0.18)
Estonia	2267.927 (1533.565-3138.575)	3662.139 (2486.161-5018.687)	340.5 (230.189-472.163)	336.904 (227.391-464.073)	-0.04 (-0.05, -0.02)
Eswatini	202.869 (134.394-283.004)	358.849 (238.131-502.055)	313.905 (210.737-434.537)	301.93 (203.275-418.714)	-0.13 (-0.13, -0.12)
Ethiopia	13006.358 (8682.329-18132.158)	34226.426 (22969.121-47481.695)	330.817 (223.957-456.311)	307.318 (207.919-423.615)	-0.24 (-0.25, -0.23)
Fiji	254.87 (169.199-358.872)	581.926 (385.55-814.888)	336.76 (227.311-468.531)	330.56 (221.975-458.84)	-0.06 (-0.08, -0.05)
Finland	9014.011 (6196.287-12214.913)	16662.347 (11305.594-22985.437)	362.187 (248.367-492.408)	323.968 (218.291-449.2)	-0.36 (-0.37, -0.35)
France	91989.847 (70554.699-115751.33)	163141.399 (119385.229-212767.574)	296.921 (225.454-376.723)	279.71 (202.578-366.971)	-0.19 (-0.2,

					-0.18)
Gabon	569.549 (386.284-783.632)	911.227 (614.106-1260.164)	375.915 (255.832-515.199)	367.476 (249.822-503.687)	-0.07 (-0.08, -0.06)
Gambia	197.971 (131.604-276.979)	572.853 (377.401-801.419)	251.583 (169.255-349.155)	232.765 (154.96-324.109)	-0.25 (-0.26, -0.24)
Georgia	6398.731 (4316.944-8843.559)	7575.892 (5179.926-10420.264)	339.764 (229.368-469.464)	340.495 (232.677-468.478)	0.01 (0, 0.01)
Germany	200149.039 (147609.515-258799.215)	339825.114 (245627.083-447422.248)	433.52 (318.181-564.852)	424.592 (303.768-563.546)	-0.07 (-0.09, -0.05)
Ghana	3176.535 (2091.296-4463.973)	8607.837 (5668.867-12049.045)	229.784 (153.556-320.234)	224.325 (149.659-311.414)	-0.08 (-0.08, -0.07)
Greece	18805.654 (12685.538-25784.468)	37087.063 (25412.703-50807.015)	371.015 (250.233-510.192)	355.992 (241.87-491.503)	-0.13 (-0.14, -0.13)
Greenland	29.867 (19.983-41.648)	69.347 (46.907-96.424)	412.53 (279.353-570.442)	405.16 (275.384-559.751)	-0.07 (-0.11, -0.04)
Grenada	83.212 (56.288-114.692)	91.614 (61.544-127.844)	297.33 (200.519-411.267)	292.819 (197.674-406.342)	-0.05 (-0.06, -0.04)
Guam	53.583 (35.418-75.418)	241.93 (162.785-336.793)	327.619 (219.965-456.679)	325.525 (219.625-451.214)	-0.02 (-0.04, 0)
Guatemala	2751.886 (1839.028-3836.152)	10937.521 (7405.38-15072.716)	339.705 (229.178-468.888)	336.205 (228.024-463.061)	-0.03 (-0.04, -0.03)
Guinea	2174.001 (1442.073-3021.216)	3371.559 (2255.36-4692.332)	248.425 (165.467-343.876)	234.168 (158.015-324.313)	-0.19 (-0.2, -0.18)

Guinea-Bissau	197.072 (128.807-276.315)	329.671 (215.665-464.214)	236.263 (157.147-328.172)	230.179 (154.093-319.918)	-0.09 (-0.09, -0.08)
Guyana	289.159 (194.522-401.417)	481.499 (322.587-668.619)	290.099 (196.179-400.926)	287.536 (194.12-396.826)	-0.03 (-0.04, -0.02)
Haiti	2210.371 (1470.524-3081.382)	4672.176 (3119.728-6534.269)	305.589 (205.988-422.303)	283.269 (191.579-392.602)	-0.25 (-0.25, -0.24)
Honduras	1844.695 (1242.23-2552.374)	5675.177 (3820.307-7841.868)	343.645 (232.438-474.489)	338.125 (228.804-466.048)	-0.05 (-0.05, -0.04)
Hungary	15684.699 (10616.188-21712.209)	24330.611 (16566.525-33559.942)	336.132 (227.728-465.6)	333.011 (226.167-460.191)	-0.03 (-0.05, -0.01)
Iceland	416.038 (296.691-551.263)	798.816 (574.818-1060.747)	403.313 (286.763-535.74)	365.534 (262.582-484.791)	-0.31 (-0.33, -0.3)
India	241550.106 (160830.066-337062.191)	749489.735 (500350.004-1044589.649)	237.341 (159.914-328.18)	235.764 (158.214-326.985)	-0.02 (-0.04, 0)
Indonesia	78839.923 (52971.472-109517.604)	189722.06 (126947.877-265054.328)	346.428 (234.629-478.521)	338.505 (228.352-469.081)	-0.07 (-0.08, -0.07)
Iran (Islamic Republic of)	23334.399 (15808.518-32335.082)	87936.73 (59671.359-121047.489)	411.263 (280.671-563.54)	399.491 (272.029-548.035)	-0.1 (-0.11, -0.08)
Iraq	9438.571 (6414.493-12915.931)	22203.921 (14927.148-30738.536)	404.139 (275.623-551.286)	387.228 (262.885-532.261)	-0.14 (-0.16, -0.12)
Ireland	4989.527 (3359.628-6831.202)	9578.782 (6467.242-13256.821)	365.258 (246.022-501.567)	334.541 (225.18-463.985)	-0.28 (-0.29, -0.28)

Israel	5741.618 (3851.846-7896.425)	15408.043 (10447.307-21171.302)	359.384 (241.269-495.588)	337.944 (228.37-465.015)	-0.19 (-0.21, -0.18)
Italy	106541.204 (74140.867-143702.371)	248809.366 (168523.691-340681.759)	347.398 (240.409-471.498)	402.579 (270.813-554.701)	0.47 (0.43, 0.52)
Jamaica	2078.309 (1395.193-2869.797)	3489.769 (2342.438-4827.493)	330.498 (221.601-457.32)	312.979 (210.715-432.214)	-0.17 (-0.19, -0.16)
Japan	184306.436 (125843.018-252179.493)	576269.984 (394111.794-788030.062)	345.971 (236.492-473.346)	350.194 (238.235-480.607)	0.07 (-0.02, 0.15)
Jordan	1219.324 (820.207-1695.225)	7242.943 (4903.664-10016.917)	398.452 (270.842-549.822)	401.81 (274.745-550.767)	0.03 (-0.03, 0.08)
Kazakhstan	11925.726 (8079.045-16478.926)	15444.166 (10391.324-21324.163)	339.801 (230.87-468.703)	331.888 (224.559-455.797)	-0.07 (-0.09, -0.06)
Kenya	6571.811 (4430.715-9071.791)	16494.27 (11076.55-22903.641)	319.604 (216.778-439.04)	314.079 (212.781-432.072)	-0.06 (-0.06, -0.05)
Kiribati	28.059 (18.69-39.298)	53.956 (35.953-75.238)	361.948 (244.211-501.885)	363.979 (246.465-501.914)	0.02 (0.01, 0.03)
Kuwait	544.287 (364.543-754.987)	2813.538 (1891.449-3908.123)	411.557 (279.778-564.34)	392.701 (268.557-539.254)	-0.15 (-0.16, -0.14)
Kyrgyzstan	2913.45 (1981.148-3999.205)	4133.92 (2792.876-5694.084)	337.402 (229.658-463.275)	340.203 (231.616-465.674)	0.03 (0.02, 0.04)
Lao People's Democratic Republic	1568.599 (1047.51-2193.393)	3764.481 (2518.037-5219.684)	341.324 (230.4-471.585)	334.037 (225.632-460.435)	-0.07 (-0.08, -0.06)
Latvia	4054.772 (2742.865-5564.734)	5466.939 (3749.064-7502.172)	342.368 (231.541-470.855)	343.438 (234.144-473.612)	0.01 (0, 0.02)

Lebanon	2400.568 (1618.888-3314.719)	9166.701 (6263.144-12506.759)	417.204 (282.991-573.437)	419.672 (286.359-573.644)	0.01 (-0.01, 0.03)
Lesotho	738.463 (493.198-1023.893)	790.844 (530.774-1099.368)	330.092 (221.943-456.003)	325.379 (220.697-450.076)	-0.05 (-0.06, -0.04)
Liberia	664.271 (440.643-929.651)	1101.312 (724.606-1541.578)	232.334 (155.163-323.578)	224.514 (150.523-310.226)	-0.11 (-0.13, -0.09)
Libya	2154.182 (1472.123-2972.389)	5286.672 (3552.29-7302.365)	409.306 (281.567-562.012)	389.752 (264.016-535.191)	-0.16 (-0.17, -0.15)
Lithuania	5084.923 (3456.287-6997.004)	7770.763 (5275.809-10645.586)	337.754 (229.347-466.04)	336.75 (227.275-464.11)	0 (-0.03, 0.04)
Luxembourg	558.094 (377.16-769.221)	1033.34 (708.359-1419.694)	301.071 (202.969-416.8)	260.971 (178.233-359.254)	-0.47 (-0.53, -0.4)
Madagascar	3780.73 (2554.228-5228.386)	6742.841 (4480.218-9467.099)	313.958 (214.111-432.146)	298.028 (201.91-412.455)	-0.17 (-0.17, -0.16)
Malawi	2682.723 (1800.466-3747.458)	5279.575 (3521.003-7362.694)	314.868 (213.316-435.394)	312.806 (211.111-431.804)	-0.02 (-0.03, -0.01)
Malaysia	9071.162 (6052.506-12610.45)	26046.428 (17301.057-36416.298)	346.922 (232.877-480.117)	332.467 (221.907-462.978)	-0.13 (-0.15, -0.11)
Maldives	62.414 (41.404-86.969)	309.203 (207.87-430.508)	328.141 (220.49-453.262)	338.227 (229.012-468.412)	0.1 (0.09, 0.11)
Mali	2124.623 (1403.461-2960.97)	4711.813 (3130.958-6586.306)	244.701 (163.652-338.116)	234.649 (158.007-324.813)	-0.14 (-0.14, -0.13)

Malta	489.37 (331.694-674.796)	1295.27 (876.912-1778.739)	366.851 (248.875-505.925)	340.273 (229.15-469.322)	-0.24 (-0.26, -0.23)
Marshall Islands	11.663 (7.759-16.347)	20.509 (13.51-28.94)	320.942 (216.144-445.707)	310.491 (208.301-431.5)	-0.11 (-0.11, -0.1)
Mauritania	626.518 (419.609-873.644)	1315.964 (873.18-1840.468)	249.534 (168.045-346.367)	231.614 (154.866-322.509)	-0.24 (-0.25, -0.23)
Mauritius	615.697 (410.748-857.428)	1882.683 (1259.792-2613.822)	335.65 (225.566-465.043)	333.77 (223.789-461.906)	-0.02 (-0.03, -0.01)
Mexico	36268.848 (24460.929-50098.562)	110466.595 (74419.072-153880.39)	316.747 (214.402-437.125)	290.326 (196.01-403.65)	-0.27 (-0.3, -0.25)
Micronesia (Federated States of)	42.987 (28.91-59.94)	56.313 (37.599-78.639)	357.868 (242.299-495.989)	360.603 (243.758-498.138)	0.03 (0.02, 0.03)
Monaco	104.421 (70.907-143.894)	135.764 (91.684-187.567)	368.35 (248.971-510.652)	339.708 (228.323-471.156)	-0.26 (-0.27, -0.25)
Mongolia	952.197 (644.86-1313.07)	1818.689 (1222.377-2513.552)	341.744 (232.877-469.319)	346.034 (235.137-475.064)	0.05 (0.03, 0.06)
Montenegro	654.138 (442.913-906.858)	1006.479 (681.323-1389.019)	342.389 (232.071-474.449)	333.574 (225.771-460.395)	-0.08 (-0.11, -0.05)
Morocco	16234.105 (11087.433-22355.21)	36513.885 (24766.725-50427.735)	408.796 (280.114-561.335)	381.924 (260.062-525.784)	-0.22 (-0.23, -0.2)
Mozambique	4333.517 (2901.284-6026.99)	7516.127 (5042.617-10402.379)	321.798 (217.914-443.896)	311.672 (212.033-426.554)	-0.1 (-0.11, -0.1)

Myanmar	19762.279 (13274.787-27387.407)	44027.744 (29350.874-61091.831)	365.112 (247.498-502.513)	337.837 (226.514-466.626)	-0.25 (-0.26, -0.24)
Namibia	454.08 (305.302-631.668)	1012.397 (678.571-1411.822)	320.108 (216.928-441.755)	309.072 (209.096-428.131)	-0.11 (-0.12, -0.1)
Nauru	2.919 (1.931-4.103)	4.143 (2.761-5.811)	331.035 (221.962-458.692)	340.466 (230.538-472.316)	0.09 (0.08, 0.1)
Nepal	5686.595 (3806.021-7938.109)	14856.356 (9928.165-20673.6)	272.491 (184.47-376.746)	242.893 (163.393-336.672)	-0.37 (-0.38, -0.36)
Netherlands	26946.621 (19664.945-34846.841)	48272.056 (34023.059-64425.28)	381.175 (277.058-494.997)	363.833 (255.024-487.735)	-0.15 (-0.16, -0.14)
New Zealand	4957.095 (3338.789-6853.911)	11097.387 (7455.75-15262.724)	382.45 (257.555-528.99)	360.985 (241.845-497.685)	-0.18 (-0.2, -0.17)
Nicaragua	1443.328 (973.407-1997.915)	4883.036 (3292.612-6732.071)	344.88 (234.051-475.302)	342.393 (231.405-471.198)	-0.02 (-0.03, -0.02)
Niger	1432.718 (952.999-2015.441)	4296.208 (2819.795-6051.212)	248.72 (168.284-345.712)	234.092 (155.958-326.078)	-0.19 (-0.2, -0.18)
Nigeria	24794.806 (16446.511-34562.546)	45312.399 (29841.724-63534.189)	225.383 (150.636-312.603)	205.622 (137.333-285.644)	-0.3 (-0.32, -0.28)
Niue	2.737 (1.845-3.792)	2.171 (1.445-3.023)	329.338 (221.017-457.895)	320.483 (213.479-446.106)	-0.09 (-0.09, -0.08)
North Macedonia	1753.802 (1178.905-2443.191)	3033.476 (2046.897-4185.013)	334.138 (225.682-463.211)	331.966 (224.687-456.976)	-0.02 (-0.03, -0.01)

Northern Mariana Islands	10.04 (6.594-14.211)	37.958 (25.104-53.119)	327.173 (221.618-455.338)	322.437 (215.124-446.882)	-0.05 (-0.08, -0.02)
Norway	10639.942 (7314.057-14450.726)	13148.554 (8877.763-18184.372)	407.885 (279.168-556.383)	341.465 (229.666-473.056)	-0.57 (-0.59, -0.54)
Oman	636.272 (428.491-887.708)	1618.884 (1077.163-2266.307)	397.088 (270.266-548.237)	371.507 (251.286-513.2)	-0.22 (-0.24, -0.19)
Pakistan	37268.583 (24949.83-51999.683)	67818.323 (44883.095-94484.074)	252.255 (169.381-350.626)	235.403 (157.482-325.144)	-0.22 (-0.23, -0.22)
Palau	7.653 (5.047-10.82)	15.915 (10.475-22.403)	320.134 (213.417-449.915)	310.635 (206.768-433.21)	-0.1 (-0.11, -0.08)
Palestine	960.764 (654.462-1325.394)	2402.201 (1622.838-3325.27)	407.677 (278.457-559.906)	392.987 (268.117-540.299)	-0.13 (-0.14, -0.12)
Panama	1472.17 (994.403-2036.521)	4943.26 (3322.156-6864.197)	329.223 (223.109-454.655)	324.543 (218.377-450.356)	-0.04 (-0.05, -0.04)
Papua New Guinea	1228.731 (814.373-1718.07)	3500.401 (2322.822-4894.65)	358.541 (242.629-495.138)	337.234 (227.298-465.477)	-0.2 (-0.21, -0.19)
Paraguay	2430.613 (1632.456-3364.433)	6135.358 (4129.482-8480.751)	368.009 (248.041-507.836)	345.841 (233.725-476.492)	-0.2 (-0.21, -0.19)
Peru	8297.036 (5523.562-11518.037)	25916.801 (17491.413-35832.385)	235.924 (157.785-326.293)	233.856 (158.255-322.754)	-0.03 (-0.04, -0.02)
Philippines	25714.684 (17319.471-35514.307)	72091.912 (48507.449-99963.481)	355.586 (241.304-489.594)	340.29 (230.298-469.281)	-0.14 (-0.15, -0.13)

Poland	48711.068 (32865.496-66990.266)	88407.298 (59758.998-121488.918)	360.202 (243.437-495.755)	341.369 (229.89-469.914)	-0.17 (-0.18, -0.16)
Portugal	15775.265 (10609.773-21876.521)	35666.188 (24222.682-48848.892)	354.718 (238.514-492.623)	349.433 (235.807-481.161)	-0.05 (-0.05, -0.04)
Puerto Rico	3509.333 (2376.225-4838.352)	8775.002 (5933.247-12145.288)	298.329 (201.784-411.934)	292.152 (197.196-405.078)	-0.07 (-0.08, -0.06)
Qatar	75.166 (49.509-106.017)	640.049 (412.32-904.512)	386.618 (263.652-529.945)	378.072 (254.656-518.076)	-0.07 (-0.09, -0.05)
Republic of Korea	26565.754 (18406.628-35954.893)	117489.28 (81676.367-159082.33)	381.486 (266.835-513.292)	372.301 (258.443-504.723)	-0.07 (-0.11, -0.04)
Republic of Moldova	3862.061 (2607.809-5351.225)	6801.067 (4614.136-9315.726)	330.526 (223.911-457.117)	334.486 (226.892-458.126)	0.04 (0.03, 0.05)
Romania	26479.91 (17801.941-36619.024)	46175.036 (31478.097-63496.428)	333.272 (225.18-460.566)	332.562 (225.853-458.592)	-0.01 (-0.01, 0)
Russian Federation	183437.941 (123983.912-252116.429)	279805.527 (190294.209-384458.553)	349.727 (236.921-481.263)	346.21 (235.02-476.137)	-0.03 (-0.04, -0.02)
Rwanda	1926.887 (1293.255-2686.12)	4608.579 (3087.536-6382.726)	319.724 (216.824-442.013)	318.349 (216.163-437.594)	-0.01 (-0.02, 0)
Saint Kitts and Nevis	34.803 (23.548-48.384)	48.451 (32.322-67.465)	281.663 (190.178-392.772)	278.945 (187.996-385.55)	-0.03 (-0.04, -0.03)
Saint Lucia	77.517 (52.137-107.525)	222.618 (150.924-309.021)	295.16 (198.73-410.231)	286.713 (194.411-397.886)	-0.1 (-0.1, -0.09)
Saint Vincent and the	67.295 (45.505-92.362)	127.747 (86.036-177.188)	303.512 (205.042-417.652)	288.447 (194.437-400.22)	-0.16 (-0.18, -0.15)

Grenadines					
Samoa	70.42 (46.978-98.246)	124.743 (82.225-173.673)	333.017 (223.636-462.369)	323.732 (214.795-448.891)	-0.09 (-0.1, -0.09)
San Marino	47.22 (32.175-64.767)	105.89 (71.326-146.43)	371.296 (252.646-510.275)	331.294 (222.088-458.988)	-0.37 (-0.39, -0.34)
Sao Tome and Principe	42.196 (28.116-58.947)	60.225 (39.481-84.256)	234.554 (157.02-327.096)	219.281 (145.393-304.859)	-0.22 (-0.23, -0.2)
Saudi Arabia	5403.055 (3629.496-7562.194)	13064.429 (8591.714-18453.16)	380.401 (257.857-528.436)	359.791 (242.637-497.933)	-0.19 (-0.21, -0.16)
Senegal	1955.529 (1310.431-2724.064)	4513.219 (2975.107-6350.421)	245.588 (165.79-340.331)	228.95 (152.248-319.997)	-0.23 (-0.23, -0.22)
Serbia	9634.769 (6509.835-13415.982)	19542.527 (13213.594-26960.877)	331.988 (225.162-460.066)	333.536 (225.198-460.642)	0.01 (0.01, 0.02)
Seychelles	62.851 (42.212-87.427)	108.143 (72.159-150.734)	337.971 (226.977-470.041)	326.368 (218.745-453.288)	-0.11 (-0.12, -0.11)
Sierra Leone	1360.092 (907.34-1889.025)	2200.862 (1463.348-3073.711)	250.265 (167.828-346.372)	236.461 (158.753-328.496)	-0.18 (-0.19, -0.17)
Singapore	1571.587 (1119.77-2072.001)	7763.979 (5662.702-10107.576)	273.498 (197.073-357.659)	283.436 (207.406-367.758)	0.12 (0.1, 0.14)
Slovakia	6379.005 (4302.653-8829.268)	10646.475 (7238.62-14671.862)	336.574 (227.485-466.038)	331.19 (225.017-456.547)	-0.05 (-0.06, -0.04)
Slovenia	2665.753 (1790.684-3695.887)	5733.44 (3903.434-7883.401)	328.916 (221.109-456.434)	330.114 (223.941-455.583)	0.01 (0, 0.02)

Solomon Islands	90.229 (59.73-126.595)	256.155 (169.852-357.136)	339.311 (227.495-469.784)	339.793 (228.286-469.957)	0.01 (0, 0.01)
Somalia	1475.561 (979.906-2057.766)	3589.613 (2382.441-5026.278)	322.011 (217.949-443.557)	318.81 (216.447-439.339)	-0.03 (-0.04, -0.03)
South Africa	19442.72 (13174.026-26845.223)	39714.686 (26792.723-55049.07)	341.1 (231.748-469.744)	321.964 (217.978-445.097)	-0.19 (-0.2, -0.17)
South Sudan	1980.002 (1331.78-2755.11)	2565.211 (1709.998-3574.162)	315.53 (214.307-436.516)	296.231 (200.575-408.568)	-0.2 (-0.21, -0.19)
Spain	65154.502 (49006.747-82872.492)	123769.741 (85383.427-167984.285)	349.696 (261.898-446.654)	306.205 (210.408-417.573)	-0.54 (-0.66, -0.41)
Sri Lanka	9120.69 (6121.451-12706.089)	25785.846 (17327.561-35958.226)	332.458 (224.976-460.927)	321.025 (216.361-446.415)	-0.11 (-0.13, -0.1)
Sudan	9489.11 (6374.478-13080.53)	18537.903 (12451.377-25653.387)	402.605 (272.969-551.821)	375.849 (254.912-516.929)	-0.22 (-0.24, -0.21)
Suriname	232.455 (156.648-321.788)	579.514 (392.909-797.668)	313.743 (212.418-432.597)	299.312 (203.561-411.09)	-0.15 (-0.17, -0.13)
Sweden	24220.482 (17358.537-32252.11)	33510.675 (22935.278-45742.21)	411.413 (292.503-551.708)	377.351 (257.056-517.205)	-0.27 (-0.3, -0.25)
Switzerland	14539.157 (9921.732-19843.861)	24775.981 (16791.29-33994.805)	372.238 (252.922-510.445)	338.372 (228.501-465.643)	-0.3 (-0.35, -0.25)
Syrian Arab Republic	5625.206 (3809.197-7777.665)	13050.94 (8798.814-17997.347)	416.453 (283.897-572.581)	387.665 (263.008-531.899)	-0.23 (-0.26, -0.21)

Taiwan (Province of China)	10336.647 (7003.94-14078.976)	43204.179 (30331.641-57611.088)	272.482 (186.294-368.334)	293.057 (206.201-389.763)	0.25 (0.2, 0.3)
Tajikistan	2618.659 (1771.128-3602.466)	4156.953 (2785.396-5805.07)	334.38 (226.631-459.286)	313.595 (212.072-432.774)	-0.21 (-0.22, -0.19)
Thailand	27745.683 (18821.341-38381.258)	112595.356 (75964.183-156374.367)	306.218 (209.263-421.542)	308.514 (208.187-428.269)	0.03 (0.01, 0.05)
Timor-Leste	213.618 (142.785-297.949)	768.391 (519.688-1068.025)	359.59 (243.866-495.721)	338.174 (229.676-468.002)	-0.2 (-0.21, -0.19)
Togo	673.017 (444.413-941.572)	1900.005 (1250.799-2668.066)	242.175 (161.962-335.836)	233.79 (156.796-324.665)	-0.12 (-0.12, -0.11)
Tokelau	1.314 (0.88-1.83)	1.63 (1.095-2.275)	332.657 (223.375-462.614)	326.724 (219.424-455.823)	-0.06 (-0.07, -0.05)
Tonga	48.981 (32.917-68.244)	83.793 (56.329-116.018)	343.904 (232.592-477.365)	333.996 (224.892-462.225)	-0.1 (-0.1, -0.09)
Trinidad and Tobago	734.535 (499.631-1017.934)	1820.908 (1224.429-2516.075)	301.189 (205.184-417.903)	294.552 (198.243-406.528)	-0.07 (-0.08, -0.07)
Tunisia	5479.534 (3695.658-7511.546)	15844.558 (10784.955-21648.723)	427.021 (290.145-582.708)	401.268 (273.936-547.073)	-0.2 (-0.22, -0.17)
Turkey	40370.057 (27496.114-55160.466)	117990.915 (79847.468-161827.335)	439.794 (301.517-598.495)	415.527 (282.007-568.602)	-0.19 (-0.2, -0.17)
Turkmenistan	1643.202 (1107.551-2269.733)	3296.345 (2206.324-4594.835)	336.228 (227.924-462.109)	319.785 (215.829-442.765)	-0.16 (-0.17, -0.15)

Tuvalu	4.99 (3.327-7.001)	9.158 (6.112-12.733)	346.802 (234.246-481.177)	338.802 (227.551-469.005)	-0.07 (-0.09, -0.06)
Uganda	4803.017 (3226.335-6674.563)	10730.739 (7168.711-14960.278)	310.938 (210.309-429.72)	306.736 (207.264-424.261)	-0.04 (-0.05, -0.03)
Ukraine	77995.967 (52514.674-107872.073)	93467.279 (63267.935-128619.64)	355.581 (239.503-491.827)	345.233 (233.055-476.211)	-0.09 (-0.11, -0.08)
United Arab Emirates	312.963 (204.3-442.194)	2323.608 (1467.494-3376.13)	369.03 (248.939-510.292)	339.035 (227.229-472.385)	-0.28 (-0.3, -0.26)
United Kingdom	112868.766 (77786.114-153271.078)	164947.027 (113259.711-224021.473)	342.277 (235.173-467.107)	322.434 (219.844-440.21)	-0.2 (-0.21, -0.18)
United Republic of Tanzania	8090.989 (5423.322-11192.186)	18873.201 (13002.439-25563.399)	319.766 (216.526-439.472)	294.18 (204.909-396.07)	-0.27 (-0.28, -0.26)
United States of America	471555.656 (324074.676-643348.448)	822911.394 (562874.4-1125402.857)	414.072 (284.141-566.113)	392.205 (267.579-536.806)	-0.18 (-0.18, -0.17)
United States Virgin Islands	63.413 (42.534-88.699)	181.429 (122.124-252.284)	291.366 (196.612-405.057)	282.511 (189.251-394.248)	-0.1 (-0.11, -0.09)
Uruguay	4421.758 (3009.146-6044.972)	7016.806 (4773.686-9634.519)	336.618 (228.742-460.825)	319.923 (216.602-440.731)	-0.16 (-0.19, -0.13)
Uzbekistan	11115.799 (7481.439-15296.601)	20088.386 (13367.628-27897.674)	318.763 (214.617-439.394)	317.216 (212.767-437.194)	-0.01 (-0.02, -0.01)
Vanuatu	40.44 (26.514-57.166)	119.028 (78.508-167.382)	320.608 (214.571-446.735)	314.56 (210.588-436.804)	-0.06 (-0.08,

					-0.05)
Venezuela (Bolivarian Republic of)	10207 (6944.865-13999.99)	34325.267 (23336.777-47336.835)	379.664 (259.194-519.616)	371.523 (253.673-510.392)	-0.07 (-0.08, -0.06)
Viet Nam	39851.991 (26734.358-55356.296)	90171.717 (60719.941-125405.881)	348.156 (234.282-482.409)	328.875 (222.658-455.938)	-0.19 (-0.2, -0.18)
Yemen	4712.072 (3191.384-6499.083)	13409.667 (9047.822-18495.134)	430.865 (294.662-589.073)	397.642 (270.405-544.537)	-0.26 (-0.27, -0.25)
Zambia	1986.208 (1320.625-2766.313)	4722.88 (3175.063-6572.46)	305.762 (205.813-423.06)	306.635 (209.401-422.57)	0.01 (0, 0.02)
Zimbabwe	2997.649 (2004.586-4197.172)	4505.107 (2989.982-6297.611)	317.727 (214.737-441.579)	306.008 (205.972-423.628)	-0.12 (-0.13, -0.11)

ASIR: age-standardized incidence rate; AAPC: average annual percentage change; SDI: socio-demographic index;

UI:uncertainty interval, CI: confidence interval;

Supplement table 3: The age-standardized disability-adjusted life years and mortality rate of dementia and its trends.

	ASDR	ASMR

	1990 year	2021 year	AAPC （95%CI）	1990 year	2021 year	AAPC （95%CI）
Afghanistan	1805.783 (739.848-4306.288)	1725.825 (727.427-4016.157)	-0.15 (-0.17, -0.12)	105.494 (24.634-296.665)	99.385 (23.451-269.671)	-0.2 (-0.22, -0.17)
Albania	1203.846 (550.665-2675.709)	1170.479 (539.096-2617.138)	-0.1 (-0.18, -0.03)	64.605 (14.865-177.607)	62.202 (13.904-176.34)	-0.14 (-0.25, -0.03)
Algeria	1525.27 (685.643-3524.569)	1431.085 (648.113-3199.741)	-0.2 (-0.21, -0.19)	83.717 (19.085-237.615)	78.24 (17.887-214.973)	-0.21 (-0.25, -0.18)
American Samoa	1298.467 (563.33-2997.054)	1210.834 (543.023-2712.66)	-0.22 (-0.25, -0.18)	72.165 (16.533-200.367)	67.131 (15.306-185.564)	-0.23 (-0.27, -0.19)
Andorra	1341.758 (618.143-2859.849)	1249.597 (573.172-2728.054)	-0.24 (-0.3, -0.18)	76.325 (18.362-198.361)	71.938 (17.857-192.063)	-0.2 (-0.29, -0.11)
Angola	1529.208 (671.37-3548.422)	1704.525 (711.321-4055.794)	0.36 (0.32, 0.39)	84.239 (18.911-238.766)	99.783 (23.438-280.323)	0.56 (0.5, 0.61)
Antigua and Barbuda	975.01 (458.689-2181.96)	931.637 (438.131-2094.074)	-0.14 (-0.18, -0.1)	49.916 (11.571-141.302)	47.144 (10.901-134.682)	-0.15 (-0.23, -0.08)
Argentina	1142.501 (529.263-2534.877)	1104.072 (509.625-2402.101)	-0.11 (-0.13, -0.09)	62.363 (14.825-172.485)	60.375 (14.723-161.382)	-0.1 (-0.13, -0.07)
Armenia	1145.518 (537.884-2526.001)	1190.507 (545.079-2610.234)	0.11 (0.04, 0.18)	59.68 (13.898-166.235)	63.761 (15.167-171.817)	0.2 (0.09, 0.31)
Australia	1311.471 (609.699-2861.194)	1190.94 (541.037-2556.836)	-0.31 (-0.34, -0.27)	72.295 (17.462-194.845)	68.382 (16.966-177.76)	-0.18 (-0.23, -0.14)

Austria	1340.603 (613.159-2994.685)	1272.36 (591.37-2740.232)	-0.16 (-0.18, -0.14)	76.012 (17.994-208.727)	72.995 (18.3-189.872)	-0.11 (-0.14, -0.09)
Azerbaijan	1186.517 (549.607-2628.335)	1148.402 (529.131-2586.538)	-0.11 (-0.15, -0.06)	63.299 (14.751-175.544)	61.351 (14.338-174.206)	-0.1 (-0.18, -0.02)
Bahamas	962.646 (457.194-2115.401)	933.008 (440.807-2036.214)	-0.11 (-0.16, -0.06)	48.371 (11.285-135.881)	47.081 (10.682-132.967)	-0.09 (-0.17, -0.01)
Bahrain	1553.927 (691.429-3565.881)	1388.543 (627.92-3203.23)	-0.37 (-0.42, -0.31)	87.44 (20.474-248.539)	76.518 (17.461-217.581)	-0.42 (-0.51, -0.33)
Bangladesh	871.772 (387.552-1993.363)	928.522 (395.784-2225.798)	0.23 (0.13, 0.32)	46.003 (10.454-131.357)	51.583 (11.563-153.059)	0.41 (0.25, 0.58)
Barbados	953.15 (453.741-2112.575)	937.402 (440.232-2050.145)	-0.05 (-0.15, 0.04)	47.306 (10.747-136.224)	47.729 (10.733-132.666)	0.03 (-0.11, 0.17)
Belarus	1189.496 (556.459-2642.282)	1176.638 (548.124-2596.229)	-0.03 (-0.07, 0.01)	62.812 (14.58-175.622)	61.466 (14.285-172.6)	-0.07 (-0.14, 0.01)
Belgium	1516.382 (697.213-3283.675)	1366.028 (629.768-2890.343)	-0.33 (-0.4, -0.26)	87.002 (21.285-230.376)	79.083 (20.575-199.626)	-0.31 (-0.37, -0.24)
Belize	987.609 (470.415-2127.951)	959.481 (455.6-2080.658)	-0.09 (-0.17, -0.02)	49.35 (11.609-134.033)	48.377 (11.365-131.313)	-0.06 (-0.17, 0.06)
Benin	965.462 (417.923-2249.632)	944.07 (394.905-2232.62)	-0.07 (-0.13, -0.01)	53.724 (12.215-153.023)	55.09 (12.476-155.957)	0.07 (0.01, 0.13)
Bermuda	996.981 (469.747-2193.778)	964.609 (460.962-2034.131)	-0.09 (-0.28, 0.1)	50.831 (11.705-143.203)	49.128 (11.754-131.112)	-0.06 (-0.37, 0.25)

Bhutan	889.88 (393.422–1997.868)	1006.792 (408.694–2415.627)	0.41 (0.37, 0.44)	46.346 (9.664–130.027)	58.634 (13.276–166.681)	0.77 (0.73, 0.81)
Bolivia (Plurinational State of)	877.833 (392.124–2030.853)	878.966 (395.323–1967.808)	0.01 (−0.02, 0.03)	46.268 (10.406–132.899)	46.752 (10.636–128.599)	0.03 (0, 0.07)
Bosnia and Herzegovina	1152.821 (535.238–2559.985)	1118.611 (530.252–2400.657)	−0.1 (−0.14, −0.05)	60.893 (14.268–168.215)	58.461 (13.461–155.857)	−0.13 (−0.2, −0.05)
Botswana	1253.281 (542.524–2985.18)	1215.06 (533.79–2790.431)	−0.1 (−0.21, 0)	68.743 (14.898–202.744)	67.481 (15.623–186.034)	−0.05 (−0.2, 0.1)
Brazil	1529.38 (681.283–3442.656)	1505.591 (683.687–3299.278)	−0.05 (−0.07, −0.04)	84.114 (20.703–226.195)	81.525 (20.732–212.185)	−0.1 (−0.13, −0.06)
Brunei Darussalam	1154.532 (510.281–2663.783)	1185.71 (524.183–2625.95)	0.07 (0.02, 0.11)	67.05 (15.617–187.527)	69.504 (16.489–187.143)	0.1 (0.03, 0.17)
Bulgaria	1147.157 (531.873–2587.51)	1138.178 (534.572–2528.814)	−0.03 (−0.07, 0.01)	59.641 (13.344–171.679)	59.854 (13.855–170.109)	0 (−0.03, 0.03)
Burkina Faso	1117.979 (458.597–2642.665)	1021.19 (421.615–2419.86)	−0.29 (−0.39, −0.18)	66.128 (15.125–184.909)	60.554 (13.784–169.694)	−0.28 (−0.41, −0.15)
Burundi	1232.807 (552.293–2813.379)	1289.897 (536.719–3089.536)	0.15 (0.12, 0.18)	67.363 (15.094–188.547)	74.823 (17.02–212.915)	0.35 (0.28, 0.42)
Cabo Verde	907.442 (407.052–2041.326)	923.166 (403.776–2104.814)	0.05 (0, 0.1)	49.371 (11.279–136.67)	52.397 (12.04–143.595)	0.2 (0.12, 0.27)
Cambodia	1232.266 (559.674–2783.713)	1384.619 (596.577–3238.722)	0.38 (0.36, 0.41)	63.199 (14.311–178.319)	76.911 (17.547–217.557)	0.64 (0.6, 0.69)
Cameroon	1033.781 (425.105–2427.701)	972.842 (399.917–2403.692)	−0.22 (−0.27, −0.16)	61.109 (13.93–171.085)	57.505 (12.666–170.621)	−0.22 (−0.29, −0.15)

Canada	1346.61 (683.569-2769.885)	1246.273 (621.356-2543.976)	-0.26 (-0.3, -0.21)	65.821 (16.071-177.189)	63.18 (15.818-164.904)	-0.11 (-0.18, -0.03)
Central African Republic	1719.679 (736.556-3908.359)	1693.818 (710.536-3839.465)	-0.05 (-0.11, 0)	98.128 (22.675-263.671)	95.507 (20.995-259.647)	-0.08 (-0.17, 0)
Chad	932.091 (408.886-2160.691)	929.759 (388.095-2182.375)	-0.01 (-0.04, 0.01)	51.362 (11.162-145.398)	53.047 (11.652-149.197)	0.1 (0.07, 0.13)
Chile	1094.392 (510.881-2422.203)	1081.244 (507.241-2315.864)	-0.04 (-0.07, 0)	58.241 (13.942-159.077)	57.996 (14.192-153.515)	-0.01 (-0.07, 0.06)
China	1596.629 (683.379-3645.309)	1680.029 (772.548-3687.56)	0.08 (-0.02, 0.19)	93.765 (21.96-255.229)	92.081 (22.419-250.032)	-0.09 (-0.17, 0)
Colombia	1072.793 (515.717-2345.22)	1072.211 (522.306-2267.935)	-0.01 (-0.03, 0.01)	53.702 (12.803-149.346)	53.341 (13.006-141.484)	-0.03 (-0.06, 0)
Comoros	1304.275 (565.269-2950.008)	1412.571 (577.46-3377.96)	0.25 (0.22, 0.27)	74.691 (17.11-199.84)	84.134 (19.695-232.448)	0.38 (0.33, 0.42)
Congo	1748.034 (727.567-4050.292)	1708.997 (697.208-3947.083)	-0.07 (-0.1, -0.04)	104.246 (24.267-283.14)	103.027 (23.592-276.948)	-0.03 (-0.07, 0)
Cook Islands	1332.35 (577.922-3076.505)	1186.477 (536.844-2619.168)	-0.38 (-0.41, -0.34)	75.363 (17.552-211.212)	65.416 (15.421-177.117)	-0.46 (-0.51, -0.41)
Costa Rica	1089.528 (517.588-2349.079)	1065.869 (513.691-2244.878)	-0.05 (-0.15, 0.04)	55.073 (13.051-151.318)	53.881 (13.166-142.321)	-0.06 (-0.08, -0.04)
Coted'Ivoire	978.425 (410.196-2286.664)	966.511 (401.651-2317.425)	-0.06 (-0.09, -0.02)	57.953 (13.384-159.356)	57.059 (12.543-163.732)	-0.05 (-0.09, -0.02)

Croatia	1185.793 (551.322–2634.243)	1143.398 (530.891–2518.624)	−0.11 (−0.22, 0)	62.358 (14.385–175.795)	60.751 (14.104–167.817)	−0.08 (−0.21, 0.05)
Cuba	914.529 (429.143–2050.664)	904.896 (427.297–2008.84)	−0.05 (−0.08, −0.01)	47.186 (10.989–132.798)	46.013 (10.666–130.975)	−0.09 (−0.17, −0.02)
Cyprus	1477.463 (634.215–3499.647)	1318.555 (586.142–2980.725)	−0.37 (−0.44, −0.31)	86.439 (19.499–245.648)	77.11 (17.973–215.221)	−0.37 (−0.45, −0.29)
Czechia	1150.264 (530.076–2598.57)	1143.196 (531.394–2476.45)	−0.01 (−0.06, 0.04)	61.427 (14.361–173.202)	60.84 (14.609–165.524)	−0.02 (−0.05, 0)
Democratic People's Republic of Korea	1263.204 (556.416–2877.278)	1288.579 (560.262–2938.582)	0.07 (0.05, 0.09)	69.659 (15.056–195.973)	72.335 (16.276–200.71)	0.12 (0.08, 0.17)
Democratic Republic of the Congo	1585.883 (671.718–3699.329)	1793.118 (737–4205.042)	0.4 (0.37, 0.43)	90 (20.466–252.101)	105.863 (24.315–289.863)	0.53 (0.49, 0.58)
Denmark	1203.05 (532.98–2744.303)	1151.421 (485.067–2605.556)	−0.12 (−0.23, −0.01)	69.356 (16.494–191.848)	72.056 (17.63–189.021)	0.15 (0.04, 0.26)
Djibouti	1365.613 (580.546–3222.393)	1439.708 (592.966–3340.785)	0.17 (0.14, 0.2)	78.085 (17.737–220.622)	85.919 (19.253–235.324)	0.31 (0.28, 0.33)
Dominica	984.73 (460.528–2185.384)	959.127 (450.69–2125.853)	−0.08 (−0.12, −0.05)	49.789 (11.407–143.732)	49.18 (11.482–139.677)	−0.04 (−0.09, 0)
Dominican Republic	992.614 (462.873–2171.297)	970.13 (459.97–2084.786)	−0.07 (−0.12, −0.02)	51.097 (11.584–142.356)	48.958 (11.131–134.662)	−0.13 (−0.25, −0.02)
Ecuador	838.568 (385.409–1894.688)	809.205 (372.397–1794.471)	−0.12 (−0.15, −0.08)	43.398 (9.927–123.037)	41.682 (9.074–117.869)	−0.13 (−0.22, −0.04)

Egypt	1467.848 (653.955-3406.782)	1349.241 (613.736-3031.708)	-0.27 (-0.37, -0.17)	80.899 (18.368-230.707)	72.805 (16.859-203.535)	-0.33 (-0.48, -0.19)
El Salvador	1093.091 (518.883-2386.601)	1081.005 (517.284-2270.608)	-0.02 (-0.11, 0.06)	55.8 (13.277-153.28)	54.6 (13.138-142.255)	-0.05 (-0.22, 0.12)
Equatorial Guinea	1583.339 (679.377-3704.194)	1679.948 (696.009-3924.488)	0.18 (0.12, 0.24)	88.376 (19.799-251.603)	98.731 (22.148-272.462)	0.35 (0.29, 0.41)
Eritrea	1304.937 (572.435-3022.654)	1491.99 (600.261-3540.74)	0.44 (0.42, 0.45)	71.677 (16.258-204.157)	89.748 (20.054-249.608)	0.73 (0.69, 0.78)
Estonia	1173.341 (546.786-2638.826)	1203.771 (552.886-2625.159)	0.08 (0.05, 0.11)	62.022 (14.241-176.113)	65.619 (15.613-176.215)	0.17 (0.13, 0.21)
Eswatini	1253.461 (540.233-2907.861)	1257.48 (531.539-2903.926)	0.02 (-0.01, 0.06)	69.762 (15.589-199.234)	69.831 (15.521-193.539)	0.04 (-0.01, 0.09)
Ethiopia	1410.036 (595.244-3338.937)	1440.694 (595.699-3352.182)	0.07 (0.04, 0.1)	80.199 (18.543-225.466)	85.64 (20.504-228.329)	0.21 (0.17, 0.25)
Fiji	1270.351 (556.127-2944.315)	1209.68 (545.87-2685.727)	-0.17 (-0.19, -0.14)	70.381 (15.988-202.376)	67.324 (15.362-186.322)	-0.15 (-0.22, -0.09)
Finland	1423.515 (634.203-3161.373)	1308.77 (583.31-2813.79)	-0.29 (-0.32, -0.26)	84.015 (20.466-223.317)	78.08 (19.769-199.21)	-0.26 (-0.29, -0.23)
France	1159.15 (518.682-2595.067)	1095.931 (495.207-2360.176)	-0.18 (-0.2, -0.16)	68.634 (16.489-185.441)	64.8 (16.272-166.651)	-0.18 (-0.22, -0.15)
Gabon	1769.949 (740.149-3986.984)	1757.406 (733.035-4057.961)	-0.02 (-0.05, 0.02)	105.554 (25.296-274.152)	105.88 (24.734-286.875)	0.02 (-0.04, 0.07)
Gambia	1019.562 (436.146-2361.102)	1019.812 (407.797-2489.954)	0 (-0.16, 0.16)	59.022 (13.066-166.203)	60.203 (13.553-173.994)	0.04 (0, 0.09)

Georgia	1170.368 (548.655-2604)	1191.434 (552.702-2625.27)	0.04 (-0.08, 0.17)	61.509 (14.329-172.03)	63.048 (15.315-170.39)	0.07 (-0.14, 0.28)
Germany	1526.284 (726.344-3246.475)	1519.473 (725.256-3123.707)	0 (-0.07, 0.06)	88.122 (22.285-229.293)	87.378 (22.89-219.056)	-0.01 (-0.1, 0.09)
Ghana	888.743 (385.381-2067.686)	979.141 (399.832-2384.611)	0.3 (0.26, 0.34)	50.013 (11.27-142.202)	57.892 (13.001-168.501)	0.45 (0.39, 0.52)
Greece	1322.217 (607.069-2960.364)	1283.203 (593.696-2770.103)	-0.11 (-0.14, -0.08)	74.223 (17.71-205.103)	72.374 (17.709-191.398)	-0.08 (-0.1, -0.07)
Greenland	1449.872 (670.385-3273.815)	1406.034 (661.403-3003.697)	-0.09 (-0.21, 0.03)	79.065 (17.955-224.338)	76.72 (18.23-201.694)	-0.09 (-0.22, 0.03)
Grenada	1008.172 (469.574-2224.77)	975.567 (456.203-2160.227)	-0.07 (-0.17, 0.02)	52.886 (12.23-145.842)	50.03 (11.427-141.857)	-0.04 (-0.18, 0.11)
Guam	1259.328 (558.4-2847.877)	1099.997 (539.526-2226.467)	-0.45 (-0.54, -0.36)	69.121 (16.133-192.481)	54.695 (14.781-132.374)	-0.79 (-0.93, -0.65)
Guatemala	1102.405 (511.768-2493.86)	1076.315 (509.974-2327.352)	-0.08 (-0.1, -0.07)	56.47 (12.662-161.514)	54.625 (12.577-151.38)	-0.12 (-0.15, -0.09)
Guinea	926.954 (400.69-2128.258)	959.025 (403.818-2250.611)	0.11 (0.08, 0.14)	50.902 (11.285-143.604)	55.254 (11.987-155.824)	0.26 (0.23, 0.29)
Guinea-Bissau	983.009 (409.95-2332.762)	1010.882 (411.993-2476.214)	0.08 (0.05, 0.11)	56.446 (12.557-160.862)	58.964 (12.892-173.472)	0.12 (0.08, 0.16)
Guyana	907.7 (431.153-1986.492)	919.084 (431.946-2028.133)	0.05 (-0.1, 0.2)	45.545 (10.491-129.128)	46.476 (10.328-130.925)	0.09 (-0.17, 0.35)
Haiti	1017.47 (473.204-2295.436)	962.645 (438.821-2192.182)	-0.17 (-0.2, -0.15)	51.18 (11.607-148.086)	49.27 (10.376-143.914)	-0.12 (-0.14, -0.1)

Honduras	1184.397 (547.949-2630.233)	1277.725 (560.564-2929.436)	0.25 (0.18, 0.33)	60.799 (13.832-169.827)	68.629 (15.817-194.22)	0.41 (0.31, 0.51)
Hungary	1152.499 (527.734-2596.457)	1138.064 (531.453-2467.142)	-0.03 (-0.07, 0.01)	61.925 (14.245-173.774)	60.036 (14.155-164.01)	-0.09 (-0.13, -0.06)
Iceland	1357.872 (649.937-2904.301)	1283.303 (608.437-2667.566)	-0.19 (-0.27, -0.11)	73.731 (17.971-195.94)	72.59 (18.356-183.659)	-0.07 (-0.19, 0.06)
India	782.892 (354.384-1830.76)	913.313 (391.787-2134.024)	0.51 (0.4, 0.63)	39.886 (8.711-117.689)	50.732 (11.78-140.774)	0.82 (0.6, 1.05)
Indonesia	1090.587 (517.041-2423.241)	1260.654 (561.337-2929.865)	0.47 (0.44, 0.5)	52.42 (11.315-149.715)	68.612 (15.792-197.62)	0.88 (0.82, 0.95)
Iran (Islamic Republic of)	1512.9 (692.01-3382.329)	1414.923 (652.983-3093.398)	-0.22 (-0.24, -0.2)	81.557 (19.543-222.618)	75.211 (18.063-202.277)	-0.26 (-0.29, -0.23)
Iraq	1465.854 (674.185-3190.953)	1413.425 (639.659-3146.828)	-0.11 (-0.14, -0.09)	78.137 (18.88-211.819)	76.994 (17.97-211.521)	-0.04 (-0.08, 0)
Ireland	1326.895 (603.57-2987.538)	1246.512 (577.517-2659.214)	-0.2 (-0.25, -0.15)	75.547 (17.882-209.449)	71.957 (18.056-183.842)	-0.17 (-0.24, -0.1)
Israel	1293.892 (589.302-2901.637)	1259.158 (575.329-2684.391)	-0.09 (-0.13, -0.05)	72.969 (17.413-201.016)	72.552 (18.266-185.754)	-0.03 (-0.1, 0.05)
Italy	1543.319 (674.594-3455.934)	1515.77 (704.631-3191.115)	-0.06 (-0.11, -0.02)	94.096 (23.274-248.626)	88.866 (23.262-223.546)	-0.18 (-0.23, -0.13)
Jamaica	1018.007 (499.206-2163.157)	981.422 (483.055-2088.577)	-0.11 (-0.16, -0.06)	49.051 (11.512-135.216)	47.861 (11.33-131.599)	-0.05 (-0.12, 0.02)
Japan	1375.877 (614.077-3026.886)	1362.211 (639.279-2800.121)	-0.04 (-0.07, 0)	81.185 (20.298-213.22)	78.646 (21.301-191.362)	-0.13 (-0.17,

						-0.09)
Jordan	1456.15 (674.163-3193.084)	1344.359 (636.909-2959.928)	-0.26 (-0.34, -0.18)	78.396 (18.991-212.635)	69.814 (16.549-194.965)	-0.37 (-0.47, -0.27)
Kazakhstan	1167.719 (542.423-2591.914)	1109.971 (511.166-2498.486)	-0.17 (-0.22, -0.13)	62.102 (14.612-172.585)	58.078 (13.182-167.279)	-0.22 (-0.29, -0.15)
Kenya	1266.882 (549.386-2871.45)	1435.62 (586.087-3226.994)	0.4 (0.35, 0.45)	71.332 (16.334-196.496)	85.749 (19.576-221.055)	0.59 (0.54, 0.64)
Kiribati	1328.545 (590.958-3054.008)	1446.476 (632.052-3309.829)	0.28 (0.27, 0.29)	70.954 (15.21-201.779)	81.809 (18.078-228.646)	0.46 (0.44, 0.48)
Kuwait	1506.129 (707.302-3253.719)	1367.459 (648.04-2908.011)	-0.31 (-0.51, -0.1)	80.596 (20.104-212.297)	72.766 (18.118-193.431)	-0.32 (-0.65, 0.02)
Kyrgyzstan	1183.339 (546.618-2617.074)	1156.07 (543.336-2518.103)	-0.07 (-0.12, -0.03)	63.453 (14.697-175.044)	60.023 (14.283-163.765)	-0.18 (-0.25, -0.12)
Lao People's Democratic Republic	1162.332 (543.076-2605.401)	1217.253 (548.498-2801.361)	0.15 (0.12, 0.19)	58.507 (12.793-168.377)	64.512 (14.554-187.961)	0.31 (0.28, 0.34)
Latvia	1173.691 (545.249-2620.924)	1171.506 (547.142-2571.935)	0 (-0.04, 0.04)	62.233 (14.32-175.488)	62.104 (14.592-171.699)	0 (-0.03, 0.02)
Lebanon	1414.909 (671.677-3100.834)	1374.569 (663.446-2934.065)	-0.1 (-0.16, -0.03)	73.796 (17.172-204.941)	71.179 (17.208-193.325)	-0.12 (-0.22, -0.01)
Lesotho	1235.479 (548.601-2723.407)	1286.268 (555.592-3025.538)	0.14 (0.09, 0.2)	66.15 (14.442-182.132)	70.008 (15.984-199.748)	0.22 (0.16, 0.27)
Liberia	921.114 (391.362-2227.67)	918.048 (383.505-2224.747)	-0.02 (-0.11, 0.07)	53.086 (11.924-153.813)	53.362 (11.405-154.319)	0.01 (-0.09, 0.12)

Libya	1644.821 (739.276-3553.548)	1500.136 (673.874-3336.328)	-0.3 (-0.37, -0.23)	92.003 (22.356-237.785)	83.76 (19.551-228.905)	-0.31 (-0.41, -0.21)
Lithuania	1170.039 (539.473-2607.502)	1175.325 (542.643-2588.734)	0.02 (-0.04, 0.08)	61.954 (14.428-176.628)	62.788 (14.764-174.953)	0.04 (-0.02, 0.11)
Luxembourg	973.606 (456.004-2149.69)	920.75 (422.532-2003.783)	-0.18 (-0.24, -0.11)	52.612 (12.004-148.379)	52.93 (12.813-139.599)	0.03 (-0.07, 0.13)
Madagascar	1076.721 (494.826-2435.218)	1092.264 (494.229-2473.092)	0.05 (0.01, 0.09)	56.233 (12.765-161.571)	58.966 (13.312-164.808)	0.16 (0.1, 0.21)
Malawi	1261.353 (549.726-2936.647)	1385.374 (584.103-3254.488)	0.31 (0.28, 0.35)	70.954 (16.221-199.918)	80.862 (18.349-227.082)	0.44 (0.36, 0.51)
Malaysia	1329.65 (606.942-2955.983)	1330.369 (580.203-3059.387)	-0.01 (-0.07, 0.06)	70.601 (17.303-189.39)	72.858 (17.314-201.583)	0.07 (-0.05, 0.19)
Maldives	1083.748 (509.103-2445.173)	1149.688 (539.101-2514.83)	0.19 (0.16, 0.21)	53.009 (11.807-152.142)	58.402 (13.999-162.509)	0.29 (0.27, 0.31)
Mali	1095.453 (441.958-2593.773)	1086.208 (430.394-2626.18)	-0.02 (-0.05, 0.01)	65.801 (14.864-184.122)	65.273 (14.803-183.682)	-0.02 (-0.08, 0.03)
Malta	1320.809 (605.746-2933.636)	1260.577 (582.007-2678.933)	-0.15 (-0.23, -0.06)	73.809 (17.654-201.061)	71.703 (18.335-182.55)	-0.09 (-0.22, 0.05)
Marshall Islands	1345.247 (572.015-3161.303)	1257.609 (532.682-2971.576)	-0.22 (-0.23, -0.2)	77.429 (17.511-219.996)	72.205 (16.197-206.427)	-0.23 (-0.24, -0.21)
Mauritania	989.296 (424.208-2288.748)	983.075 (412.247-2389.607)	-0.03 (-0.12, 0.06)	56.16 (12.693-157.968)	56.994 (12.296-167.296)	0.04 (-0.01, 0.08)
Mauritius	1225.488 (560.448-2745.562)	1165.295 (542.646-2585.482)	-0.18 (-0.27, -0.1)	65.471 (15.405-181.627)	60.642 (14.515-166.419)	-0.28 (-0.42, -0.15)

Mexico	924.28 (443.596-2034.907)	886.765 (421.136-1960.759)	-0.14 (-0.16, -0.12)	45.11 (10.312-128.985)	44.22 (10.334-126.557)	-0.07 (-0.17, 0.03)
Micronesia (Federated States of)	1422.165 (620.727-3336.989)	1399.742 (615.766-3265.81)	-0.05 (-0.07, -0.03)	79.224 (18.036-226.167)	78.375 (17.798-223.285)	-0.03 (-0.06, -0.01)
Monaco	1355.405 (622.085-2998.87)	1346.253 (612.731-2866.971)	-0.02 (-0.04, -0.01)	77.119 (17.914-212.849)	81.302 (20.082-203.195)	0.17 (0.15, 0.19)
Mongolia	1233.347 (560.747-2745.852)	1201.72 (559.02-2669.796)	-0.08 (-0.2, 0.04)	67.676 (15.853-187.066)	64.702 (15.223-179.383)	-0.19 (-0.37, -0.01)
Montenegro	1171.733 (546.219-2583.666)	1146.217 (527.498-2547.358)	-0.06 (-0.14, 0.01)	61.502 (14.256-168.776)	61.136 (14.057-169.875)	0 (-0.11, 0.11)
Morocco	1487.871 (690.22-3296.5)	1432.939 (640.307-3224.335)	-0.12 (-0.13, -0.11)	79.075 (18.614-217.791)	78.073 (18.095-216.756)	-0.04 (-0.05, -0.03)
Mozambique	1364.456 (581.206-3157.986)	1465.163 (594.516-3566.108)	0.26 (0.2, 0.31)	80.208 (18.88-222.82)	88.896 (20.102-255.517)	0.37 (0.29, 0.46)
Myanmar	1166.506 (552.798-2560.293)	1217.23 (545.926-2781.038)	0.15 (0.13, 0.16)	56.937 (12.783-164.257)	64.232 (14.541-185.071)	0.4 (0.38, 0.43)
Namibia	1202.833 (536.545-2744.694)	1293.227 (555.345-3077.607)	0.24 (0.22, 0.27)	64.785 (14.863-180.78)	72.487 (16.18-209.804)	0.38 (0.34, 0.41)
Nauru	1335.668 (570.115-3191.496)	1339.518 (577.029-3141.426)	0.01 (0, 0.02)	75.35 (17.135-218.481)	75.246 (16.739-218.762)	-0.01 (-0.02, 0.01)
Nepal	839.156 (394.478-1873.613)	928.612 (397.443-2160.711)	0.33 (0.3, 0.36)	41.748 (9.168-120.555)	51.729 (11.512-148.477)	0.7 (0.65, 0.75)
Netherlands	1440.211 (658.084-3105.084)	1418.917 (644.233-3045.459)	-0.05 (-0.08, -0.01)	84.878 (21.262-221.165)	83.455 (21.187-214.654)	-0.05 (-0.11, 0.01)

New Zealand	1391.171 (635.14-3052.631)	1317.914 (605.845-2838.19)	-0.18 (-0.21, -0.14)	78.032 (19.02-208.574)	74.167 (18.713-194.063)	-0.18 (-0.23, -0.13)
Nicaragua	1091.795 (527.875-2318.864)	1066.002 (516.008-2225.281)	-0.09 (-0.13, -0.06)	54.615 (13.12-147.713)	52.491 (12.622-140.702)	-0.15 (-0.2, -0.09)
Niger	988.526 (419.658-2305.72)	988.858 (409.952-2351.319)	0 (-0.05, 0.05)	56.38 (12.633-158.13)	57.729 (12.877-164.612)	0.08 (0.03, 0.13)
Nigeria	886.766 (377.616-2083.054)	924.414 (377.618-2243.865)	0.12 (0.08, 0.16)	51.867 (12.047-147.152)	56.781 (12.942-161.076)	0.27 (0.22, 0.33)
Niue	1430.047 (601.11-3346.165)	1296.891 (565.685-2984.009)	-0.32 (-0.35, -0.29)	82.973 (18.976-232.749)	74.55 (17.708-203.544)	-0.35 (-0.38, -0.31)
North Macedonia	1108.953 (522.86-2435.797)	1117.121 (517.528-2513.598)	0.02 (-0.01, 0.05)	57.377 (13.414-157.848)	58.764 (13.39-167.236)	0.07 (0.06, 0.09)
Northern Mariana Islands	1256.732 (556.008-2859.452)	1227.663 (547.372-2798.374)	-0.08 (-0.15, 0)	69.697 (15.738-195.495)	68.146 (16.088-189.106)	-0.06 (-0.12, 0)
Norway	1426.321 (664.518-3105.63)	1277.866 (582.388-2779.601)	-0.35 (-0.38, -0.32)	79.298 (19.295-212.631)	74.286 (18.45-193.461)	-0.2 (-0.24, -0.17)
Oman	1532.038 (691.68-3389.573)	1410.857 (645.657-3227.562)	-0.28 (-0.34, -0.22)	84.447 (19.942-226.5)	78.026 (19.392-216.136)	-0.27 (-0.37, -0.18)
Pakistan	928.814 (402.282-2163.616)	997.297 (406.086-2344.993)	0.22 (0.17, 0.28)	51.036 (11.1-147.546)	58.51 (13.182-162.452)	0.44 (0.38, 0.49)
Palau	1301.545 (554.579-3030.827)	1164.1 (505.605-2754.472)	-0.37 (-0.39, -0.34)	73.836 (16.56-209.239)	64.816 (14.449-188.696)	-0.42 (-0.48, -0.36)

Palestine	1478.094 (674.822–3386.723)	1379.446 (640.692–3109.701)	−0.24 (−0.26, −0.22)	79.715 (17.991–229.87)	73.733 (17.011–208.079)	−0.26 (−0.29, −0.22)
Panama	1045.383 (500.106–2240.162)	1042.167 (498.331–2166.046)	0.02 (−0.01, 0.04)	52.354 (12.592–143.471)	52.573 (12.863–136.812)	0.03 (−0.05, 0.1)
Papua New Guinea	1235.907 (563.891–2783.716)	1175.199 (525.889–2722.851)	−0.16 (−0.18, −0.14)	63.501 (13.986–180.323)	61.426 (13.752–181.275)	−0.11 (−0.12, −0.09)
Paraguay	1397.138 (638.148–3056.799)	1391.469 (619.119–3040.617)	0 (−0.07, 0.06)	74.466 (17.857–201.001)	76.534 (18.344–200.254)	0.11 (0.01, 0.21)
Peru	821.368 (371.93–1853.672)	802.1 (370.68–1737.865)	−0.06 (−0.17, 0.06)	43.066 (9.889–121.956)	41.528 (9.678–111.625)	−0.1 (−0.31, 0.1)
Philippines	1176.828 (550.102–2652.682)	1225.668 (557.647–2714.678)	0.13 (0.07, 0.19)	59.808 (13.627–171.752)	64.195 (14.998–176.916)	0.23 (0.14, 0.32)
Poland	1213.779 (564.236–2714.089)	1179.309 (546.506–2600.679)	−0.1 (−0.12, −0.08)	64.139 (15.091–179.098)	62.96 (15.188–171.201)	−0.06 (−0.11, −0.02)
Portugal	1327.427 (592.522–2991.3)	1292.877 (593.771–2775.251)	−0.09 (−0.12, −0.05)	76.614 (17.854–212.713)	74.054 (18.431–192.894)	−0.11 (−0.16, −0.06)
Puerto Rico	994.606 (465.009–2198.25)	951.362 (456.432–1998.675)	−0.16 (−0.22, −0.11)	50.422 (11.686–142.954)	47.686 (11.569–127.293)	−0.21 (−0.27, −0.15)
Qatar	1548.252 (678.912–3567.156)	1397.923 (622.5–3179.962)	−0.33 (−0.48, −0.18)	89.533 (20.584–251.377)	79.274 (18.594–222.742)	−0.42 (−0.53, −0.31)
Republic of Korea	1734.578 (740.793–3936.02)	1493.812 (683.834–3097.446)	−0.49 (−0.51, −0.46)	106.601 (26.071–285.905)	86.679 (22.461–213.034)	−0.68 (−0.73, −0.64)

Republic of Moldova	1157.999 (528.334-2614.9)	1112.57 (529.165-2393.332)	-0.13 (-0.15, -0.11)	62.026 (14.374-176.027)	56.811 (13.457-153.254)	-0.28 (-0.32, -0.24)
Romania	1143.934 (532.147-2580.413)	1143.4 (532.073-2525.577)	-0.01 (-0.03, 0.02)	60.263 (13.887-172.697)	59.912 (13.923-167.807)	-0.03 (-0.08, 0.02)
Russian Federation	1212.941 (563.678-2724.702)	1188.566 (554.639-2655.157)	-0.06 (-0.1, -0.03)	64.573 (14.98-181.537)	62.891 (14.854-173.12)	-0.08 (-0.13, -0.04)
Rwanda	1310.852 (567.665-3083.038)	1472.426 (603.216-3532.502)	0.38 (0.35, 0.42)	74.686 (17.228-213.185)	87.818 (20.588-246.145)	0.54 (0.45, 0.62)
Saint Kitts and Nevis	938.939 (437.248-2117.447)	919.096 (432.385-2032.665)	-0.08 (-0.23, 0.06)	48.257 (10.797-141.495)	46.365 (10.583-130.662)	-0.15 (-0.4, 0.11)
Saint Lucia	971.715 (451.463-2175.727)	950.607 (447.872-2089.331)	-0.1 (-0.15, -0.05)	49.683 (11.151-143.765)	48.985 (11.365-136.544)	-0.04 (-0.19, 0.12)
Saint Vincent and the Grenadines	1026.807 (475.791-2294.773)	936.846 (445.111-2065.893)	-0.31 (-0.41, -0.21)	53.15 (12.08-150.45)	47.367 (10.652-133.969)	-0.39 (-0.64, -0.14)
Samoa	1432.562 (612.963-3321.422)	1365.901 (576.412-3202.35)	-0.15 (-0.17, -0.14)	82.871 (18.851-227.393)	79.131 (18.346-223.755)	-0.15 (-0.18, -0.12)
San Marino	1275.096 (597.487-2717.113)	1148.299 (544.447-2366.933)	-0.35 (-0.46, -0.25)	70.663 (17.227-186.167)	64.193 (16.517-161.872)	-0.33 (-0.51, -0.15)
Sao Tome and Principe	929.049 (402.144-2183.672)	912.776 (384.833-2155.798)	-0.06 (-0.11, -0.01)	53.208 (12.02-150.676)	52.829 (12.236-149.978)	-0.03 (-0.11, 0.05)
Saudi Arabia	1496.805 (659.132-3380.356)	1364.338 (599.037-3127.506)	-0.31 (-0.33, -0.28)	85.082 (19.755-234.588)	77.208 (18.039-216.832)	-0.32 (-0.36, -0.29)

Senegal	987.129 (415.18-2334.026)	1043.975 (421.362-2539.399)	0.15 (0.1, 0.2)	56.497 (12.597-159.534)	62.312 (13.495-178.548)	0.31 (0.17, 0.46)
Serbia	1145.041 (525.944-2615.301)	1132.053 (524.7-2507.462)	-0.04 (-0.06, -0.02)	60.684 (13.665-175.772)	59.489 (13.681-167.766)	-0.07 (-0.11, -0.03)
Seychelles	1247.265 (571.715-2798.82)	1167.427 (527.43-2580.154)	-0.21 (-0.24, -0.18)	65.766 (15.28-184.267)	62.02 (14.17-172.072)	-0.18 (-0.22, -0.14)
Sierra Leone	909.628 (401.346-2123.466)	905.296 (387.839-2156.008)	-0.01 (-0.06, 0.03)	50.616 (11.132-146.807)	51.034 (11.532-148.37)	0.02 (-0.03, 0.07)
Singapore	1000.21 (456.596-2234.587)	978.92 (469.691-2042.935)	-0.08 (-0.13, -0.02)	56.167 (13.441-151.576)	53.468 (13.679-136.762)	-0.18 (-0.28, -0.07)
Slovakia	1172.517 (540.4-2624.164)	1155.074 (532.982-2517.574)	-0.05 (-0.09, 0)	62.468 (14.497-173.634)	61.753 (14.601-167.135)	-0.04 (-0.11, 0.03)
Slovenia	1141.157 (528.257-2532.71)	1152.089 (538.209-2457.269)	0.04 (-0.04, 0.13)	60.24 (14.084-165.351)	62.142 (15.157-165.752)	0.11 (-0.03, 0.25)
Solomon Islands	1288.583 (563.711-3046.63)	1288.859 (562.838-3018.424)	-0.01 (-0.06, 0.04)	70.058 (15.434-206.817)	70.579 (15.791-203.567)	0.02 (-0.04, 0.09)
Somalia	1241.814 (539.933-2899.224)	1329.243 (569.059-3167.136)	0.23 (0.19, 0.26)	67.892 (15.114-194.058)	73.452 (16.444-215.041)	0.27 (0.22, 0.33)
South Africa	1185.473 (545.95-2650.318)	1214.785 (537.872-2794.818)	0.08 (0.03, 0.13)	63.217 (14.483-174.902)	67.447 (15.789-188.652)	0.22 (0.15, 0.3)
South Sudan	1229.704 (524.315-2856.629)	1234.42 (513.34-2864.902)	0.02 (-0.02, 0.05)	69.015 (15.127-193.733)	70.383 (15.216-196.066)	0.06 (0.04, 0.07)
Spain	1375.551 (616.6-3029.063)	1249.256 (563.978-2658.919)	-0.33 (-0.39, -0.28)	79.327 (19.749-212.332)	74.258 (19.257-187.958)	-0.21 (-0.24, -0.18)

Sri Lanka	1118.47 (523.589-2470.16)	1102.31 (506.648-2394.215)	-0.05 (-0.1, 0.01)	56.359 (12.98-158.595)	56.521 (12.73-153.859)	0.01 (-0.09, 0.11)
Sudan	1428.95 (659.717-3321.272)	1321.052 (613.393-2908.224)	-0.26 (-0.27, -0.24)	75.354 (17.308-215.084)	69.771 (15.763-191.17)	-0.25 (-0.27, -0.24)
Suriname	997.144 (475.958-2167.303)	966.066 (463.978-2063.235)	-0.09 (-0.15, -0.02)	49.532 (11.552-138.143)	48.697 (11.013-132.396)	-0.03 (-0.16, 0.11)
Sweden	1336.062 (640.244-2859.068)	1245.672 (596.298-2576.1)	-0.22 (-0.28, -0.16)	73.639 (17.888-196.338)	69.301 (17.436-176.852)	-0.19 (-0.26, -0.12)
Switzerland	1343.209 (618.408-2930.114)	1271.169 (587.236-2662.252)	-0.17 (-0.27, -0.08)	74.324 (18.012-200.196)	73.567 (19.108-183.704)	-0.01 (-0.13, 0.11)
Syrian Arab Republic	1440.838 (673.218-3279.282)	1349.289 (618.397-3083.434)	-0.22 (-0.25, -0.18)	75.633 (17.735-214.401)	71.179 (16.051-205.28)	-0.21 (-0.29, -0.12)
Taiwan (Province of China)	1047.79 (462.576-2378.157)	1111.687 (508.53-2387.139)	0.25 (0.16, 0.35)	60.341 (14.339-165.088)	63.119 (15.718-163.892)	0.19 (-0.01, 0.4)
Tajikistan	1173.638 (537.242-2577.004)	1170.469 (522.732-2659.763)	0 (-0.04, 0.03)	62.478 (14.38-170.094)	64.499 (15.158-178.806)	0.13 (0.05, 0.2)
Thailand	1210.15 (534.015-2725.281)	1181.316 (539.613-2561.859)	-0.08 (-0.14, -0.03)	66.225 (15.427-182.988)	63.049 (15.18-163.353)	-0.16 (-0.27, -0.06)
Timor-Leste	1141.663 (543.819-2527.175)	1193.883 (539.335-2751.574)	0.14 (0.1, 0.17)	56.001 (12.356-161.376)	62.523 (14.214-180.946)	0.36 (0.31, 0.4)
Togo	969.882 (411.848-2273.621)	1018.21 (421.325-2448.356)	0.14 (0.09, 0.18)	55.419 (12.229-158.406)	60.025 (13.139-171.822)	0.23 (0.17, 0.29)

Tokelau	1468.226 (609.297–3537.918)	1333.833 (578.237–3069.82)	−0.31 (−0.36, −0.25)	84.603 (19.559–242.221)	75.851 (17.613–208.054)	−0.35 (−0.38, −0.32)
Tonga	1394.033 (615.771–3206.963)	1349.259 (593.181–3002.563)	−0.1 (−0.15, −0.05)	78.925 (18.531–218.026)	77.33 (18.174–208.346)	−0.07 (−0.15, 0)
Trinidad and Tobago	976.563 (461.486–2183.984)	940.428 (449.684–2047.94)	−0.12 (−0.17, −0.08)	49.765 (11.248–143.746)	47.025 (10.722–130.621)	−0.18 (−0.24, −0.12)
Tunisia	1575.51 (721.594–3491.707)	1451.653 (671.837–3184.066)	−0.27 (−0.3, −0.24)	84.396 (19.8–230.006)	77.734 (18.111–212.297)	−0.28 (−0.33, −0.23)
Turkey	1647.437 (751.439–3622.753)	1469.724 (686.603–3257.739)	−0.35 (−0.45, −0.25)	89.87 (21.336–244.371)	78.151 (18.652–216.29)	−0.41 (−0.6, −0.22)
Turkmenistan	1178.619 (542.669–2637.656)	1114.946 (519.93–2471.002)	−0.18 (−0.24, −0.12)	62.736 (14.772–174.551)	58.885 (13.649–163.986)	−0.19 (−0.29, −0.09)
Tuvalu	1431.096 (622.969–3447.704)	1364.183 (594.301–3218.175)	−0.15 (−0.17, −0.13)	80.585 (18.552–236.578)	77.189 (17.503–222.314)	−0.14 (−0.16, −0.11)
Uganda	1177.977 (517.186–2629.639)	1355.53 (566.536–3201.735)	0.45 (0.41, 0.5)	65.8 (15.184–179.795)	80.272 (18.756–222.165)	0.65 (0.6, 0.7)
Ukraine	1202.615 (556.599–2703.058)	1183.881 (538.499–2635.649)	−0.05 (−0.06, −0.03)	64.24 (14.789–182.534)	62.768 (14.373–175.879)	−0.08 (−0.15, −0.01)
United Arab Emirates	1515.05 (665.864–3430.53)	1277.008 (564.975–2867.276)	−0.56 (−0.76, −0.37)	86.158 (20.39–233.685)	72.219 (16.831–198.775)	−0.59 (−0.87, −0.31)
United Kingdom	1223.101 (558.501–2717.324)	1166.239 (534.044–2540.528)	−0.17 (−0.21, −0.12)	70.764 (16.962–192.344)	67.541 (16.824–177.204)	−0.17 (−0.22, −0.12)

United Republic of Tanzania	1340.911 (571.523-3096.084)	1317.513 (549.937-3108.89)	-0.06 (-0.08, -0.03)	76.826 (18.025-211.697)	77.196 (18.391-214.063)	0.02 (-0.02, 0.06)
United States of America	1579.655 (725.588-3453.979)	1522.749 (693.496-3300.485)	-0.13 (-0.14, -0.12)	88.547 (21.969-233.328)	87.146 (22.041-224.471)	-0.05 (-0.09, -0.02)
United States Virgin Islands	1009.041 (465.886-2266.826)	903.845 (430.37-1938.701)	-0.35 (-0.37, -0.34)	52.085 (12.154-150.085)	45.42 (10.344-124.665)	-0.45 (-0.48, -0.41)
Uruguay	1138.52 (529.796-2535.434)	1134.521 (519.208-2477.047)	-0.03 (-0.03, -0.02)	61.849 (14.539-170.992)	63.23 (15.415-168.602)	0.05 (0.02, 0.09)
Uzbekistan	1130.753 (518.377-2518.722)	1096.553 (505.078-2457.833)	-0.11 (-0.13, -0.08)	60.592 (14.233-166.949)	57.706 (13.515-161.641)	-0.17 (-0.25, -0.09)
Vanuatu	1239.443 (539.019-2880.773)	1239.203 (539.118-2955.515)	-0.01 (-0.07, 0.06)	68.304 (14.845-197.32)	68.964 (15.496-200.861)	0.03 (-0.02, 0.08)
Venezuela (Bolivarian Republic of)	1242.358 (592.561-2689.469)	1222.276 (582.088-2585.571)	-0.06 (-0.12, 0.01)	63.322 (15.14-173.856)	61.909 (14.686-165.269)	-0.07 (-0.17, 0.04)
Viet Nam	1357.921 (610.833-3031.638)	1423.199 (614.039-3299.987)	0.16 (0.13, 0.18)	73.549 (16.732-201.575)	81.538 (19.235-226.429)	0.34 (0.31, 0.37)
Yemen	1557.486 (715.601-3556.885)	1452.451 (662.194-3359.674)	-0.22 (-0.25, -0.2)	81.883 (18.922-230.411)	77.936 (17.091-229.342)	-0.16 (-0.18, -0.13)
Zambia	1184.266 (521.201-2734.152)	1288.866 (551.514-2987.549)	0.28 (0.25, 0.3)	65.611 (14.828-185.624)	73.423 (16.509-204.048)	0.36 (0.27, 0.46)

Zimbabwe	1299.244 (561.409-2944.213)	1245.993 (535.548-2919.374)	-0.12 (-0.17, -0.07)	74.116 (17.395-202.361)	69.039 (15.488-195.309)	-0.2 (-0.27, -0.14)

ASDR : age-standardized disability-adjusted life year rate, ASMR: age-standardised mortality rate , DALYs: disability-adjusted life year rates , AAPC: average annual percentage change, SDI: socio-demographic index, CI: confidence interval, UI: uncertainty interval.
